# Genome-Wide Analysis Reveals Hypoxic Microenvironment Is Associated With Immunosuppression in Poor Survival of Stage II/III Colorectal Cancer Patients

**DOI:** 10.3389/fmed.2021.686885

**Published:** 2021-06-15

**Authors:** Yu-feng Chen, Zhao-liang Yu, Min-yi Lv, Bin Zheng, Ying-xin Tan, Jia Ke, Xuan-hui Liu, Ze-rong Cai, Yi-feng Zou, Ping Lan, Xiao-jian Wu, Feng Gao

**Affiliations:** ^1^Department of Colorectal Surgery, The Sixth Affiliated Hospital, Sun Yat-sen University, Guangzhou, China; ^2^Guangdong Provincial Key Laboratory of Colorectal and Pelvic Floor Diseases, The Sixth Affiliated Hospital, Sun Yat-sen University, Guangzhou, China; ^3^Guangdong Institute of Gastroenterology, Guangzhou, China; ^4^The First Hospital of Lanzhou University, Lanzhou, China

**Keywords:** hypoxia-related gene signature, immunosuppression, chemoresistance, colorectal cancer, biomarker

## Abstract

**Background:** Hypoxia is associated with a poorer clinical outcome and resistance to chemotherapy in solid tumors; identifying hypoxic-related colorectal cancer (CRC) and revealing its mechanism are important. The aim of this study was to assess hypoxia signature for predicting prognosis and analyze relevant mechanism.

**Methods:** Patients without chemotherapy were selected for the identification of hypoxia-related genes (HRGs). A total of six independent datasets that included 1,877 CRC patients were divided into a training cohort and two validation cohorts. Functional annotation and analysis were performed to reveal relevant mechanism.

**Results:** A 12-gene signature was derived, which was prognostic for stage II/III CRC patients in two validation cohorts [TCGA, *n* = 509, hazard ratio (HR) = 2.14, 95% confidence interval (CI) = 1.18 – 3.89, *P* = 0.01; metavalidation, *n* = 590, HR = 2.46, 95% CI = 1.59 – 3.81, *P* < 0.001]. High hypoxic risk was correlated with worse prognosis in CRC patients without adjuvant chemotherapy (HR = 5.1, 95% CI = 2.51 – 10.35, *P* < 0.001). After integration with clinical characteristics, hypoxia-related gene signature (HRGS) remained as an independent prognostic factor in multivariate analysis. Furthermore, enrichment analysis found that antitumor immune response was suppressed in the high hypoxic group.

**Conclusions:** HRGS is a promising system for estimating disease-free survival of stage II/III CRC patients. Hypoxia tumor microenvironment may be via inhibiting immune response to promote chemoresistance in stage II/III CRC patients.

## Introduction

Colorectal cancer (CRC) is the third most common cancer type in men (10.9% of all cancers) and the second in women (9.5% of all cancers) worldwide (1). Currently, the therapeutic treatment for individual patients is mainly based on the TNM staging (2). However, for stages II and III CRC patients, the current staging method cannot provide indications of how and when to use chemotherapy, neither is the possible effectiveness of chemotherapy. As a result, chemotherapy frequently leads to diverse unpredictable responses due to risk factors such as T stage, tumor differentiation, and microsatellite instability (MSI) status (3–5). A previous study has shown that some stage II CRC patients have worse prognosis, whereas some stage III patients are overtreated (6). Therefore, investigating new reliable biomarkers can help to choose the appropriate therapy at the right time for CRC patients.

Hypoxia is a common microenvironmental condition found in most solid tumors and is associated with poor prognosis (7–9). Hypoxia-inducible factors can be activated under the hypoxia condition in the cellular environment. Evidence has shown that these factors can promote the tumor invasion and metastasis (10–12). Furthermore, recent studies have found that the hypoxic tumor microenvironment could affect the chemotherapy efficiency to CRC patients (13, 14). Therefore, it is important to identify prognostic hypoxia-inducible factors that could help to choose the appropriate therapy against the CRC.

Currently, hypoxic gene signatures are found to have prognostic and predictive effects for diverse cancers including head and neck, bladder, soft tissue sarcoma, and cervical cancers, which allow clinicians to coordinate the therapeutic agent use for patients with most benefit (7, 8, 15–18). In this study, we analyzed hypoxia-related genes from large amounts of CRC transcriptional data and created a hypoxia-related gene signature (HRGS) for CRC prognosis. The prognostic prediction value of the HRGS was systematically validated in a metavalidation cohort. In addition, we demonstrated a relevant mechanism underlying the poor survival outcome of the high hypoxic risk CRC patients at stage II/III, and this finding may help to detect out new therapeutic target for CRC patients. This would help improve therapeutic strategy for CRC patients.

## Materials and Methods

### Patients (Data Source)

This meta-analysis study used the gene expression data of frozen CRC tumor tissue samples from six public cohorts. To be included in the study, patients needed to meet the following inclusion criterion: patients with pathologic diagnosed as CRC. The exclusion criterion was patients without survival information. In total, data of 1,877 patients, including 309 CRC patients without adjuvant chemotherapy, were used. The two largest individual datasets, CIT/GSE39582 and The Cancer Genome Atlas (TCGA) CRC cohort, were used for training and independent validation. The remaining four microarray datasets (GSE14333, GSE17536, GSE37892, and GSE33113) obtained from the Gene Expression Omnibus (GEO) database were merged as a metavalidation cohort. Data of the TCGA CRC cohort were downloaded from Broad GDAC Firehose (http://gdac.broadinstitute.org/), and transcripts per million of level 3 RNA-Seq data in log2 scale were used for the analysis. Other datasets were obtained in processed format from GEO database through R using the Bioconductor package “GEOquery.” The batch effects were corrected using “combat” algorithm implemented in the R package “sva,” and *z* scores of each gene were used for the following analyses. The staging classification in each dataset was based on pathologic stage. Data were collected from September 27 to December 26, 2018.

### Construction and Validation of HRGS

The Molecular Signatures Database (MSigDB) is one of the most widely used and comprehensive databases of gene sets for performing gene set enrichment analysis (GSEA) (19–21). We created a list of hypoxia-related genes (HRGs) including all gene sets found in MSigDB (version 6.2) with the keyword “hypoxia.” In order to construct a prognostic HRGS, we assessed the association between all HRGs found in this meta-analysis and the patients' disease-free survival (DFS) in GSE39582 dataset using the log-rank test with 1,000 times randomization (80% proportion of samples each time). HRGs, which have frequently been significantly associated with the patients' DFS, were selected as candidates for the construction of HRGS. To minimize the risk of overfitting, we applied a Cox proportional hazards regression model combined with the least absolute shrinkage and selection operator (LASSO) (glmnet, version 2.0-16). The penalty parameter was estimated by 10-fold cross-validation in the training dataset at 1 SE beyond the minimum partial likelihood deviance.

In order to separate patients into low- or high-risk groups, the optimal HRGS cutoff was determined by a time-dependent receiver operating characteristic (ROC) curve (survival ROC, version 1.0.3) at 5 years in the training dataset. The Kaplan–Meier estimation method was used to estimate the ROC curve. The predictive value of HRGS that corresponded to the shortest distance between the ROC curve and the point representing both the 100% true positive rate and 0% false-positive rate was selected as the cutoff value.

The prognostic value of the HRGS was assessed in stage II/III CRC patients and patients with all stages in the training and independent validation cohorts in univariate analyses, respectively. The prognostic value of HRGS was also examined with available clinical and pathologic variables in multivariate analyses.

### Functional Annotation and Analysis

To investigate the biological characteristic of the HRGS, we conducted enrichment analysis for differentially expressed genes between hypoxic risk groups in TCGA CRC dataset using the R package “gProfileR.” The biological pathways of interest were further analyzed by GSEA in R using the Bioconductor package “HTSanalyzeR.” (22) In addition, we estimated the proportion of infiltrated immune cells and stromal cells within the tumor tissues using the ESTIMATE (Estimation of STromal and Immune cells in MAlignant Tumor tissues using Expression data) (23). The proportion of different types of immune cells such as lymphocytes, monocytes, and neutrophils and necrosis percentage were calculated using CIBERSORT (24).

### Statistical Analysis

Statistical analysis was conducted using SPSS (version 22.0.0, IBM SPSS Statistics, IBM Corp., Armonk, NY) and R software (version 3.5.1; http://www.Rproject.org). Means with standard deviations or medians with interquartile ranges were calculated for continuous values, whereas frequencies were determined for categorical values. The significance between two different groups composed of continuous values was examined using the Student *t* test. Univariate analysis of the association of HRGS and other clinical pathologic factors with DFS was conducted using log-rank test. Multivariate analysis was conducted for the factors with a *p* < 0.01 in the univariate analysis, using the Cox proportional hazards regression model. The C-index was calculated with survcomp (version 1.32.0). A *P* < 0.05 was considered statistically significant.

## Results

### Discovery and Training of the HRGS

**Figure 1:**
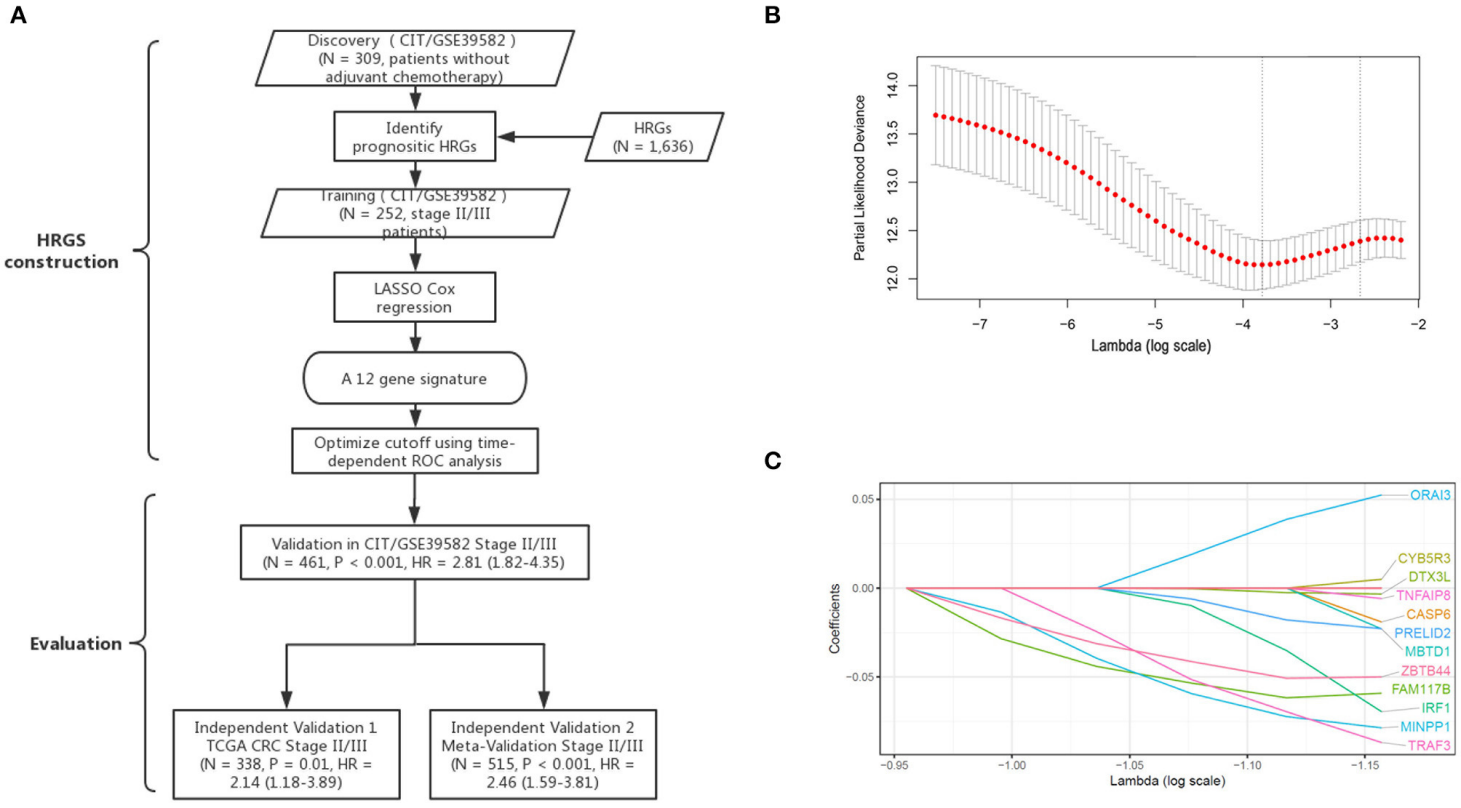
Establishment and verification of HRGS. A schematic flowchart of study design and analysis steps **(A)**. The optimal lambda in the LASSO model was chosen, and the lowest standard 5-fold crossvalidation was used. By using the minimum standard and the minimum standard of 1 SE (1 – SE standard), a vertical line was drawn at the optimal value **(B)**. Twelve hypoxia-related genes selected in LASSO COX regression **(C)**.

**Figure 2:**
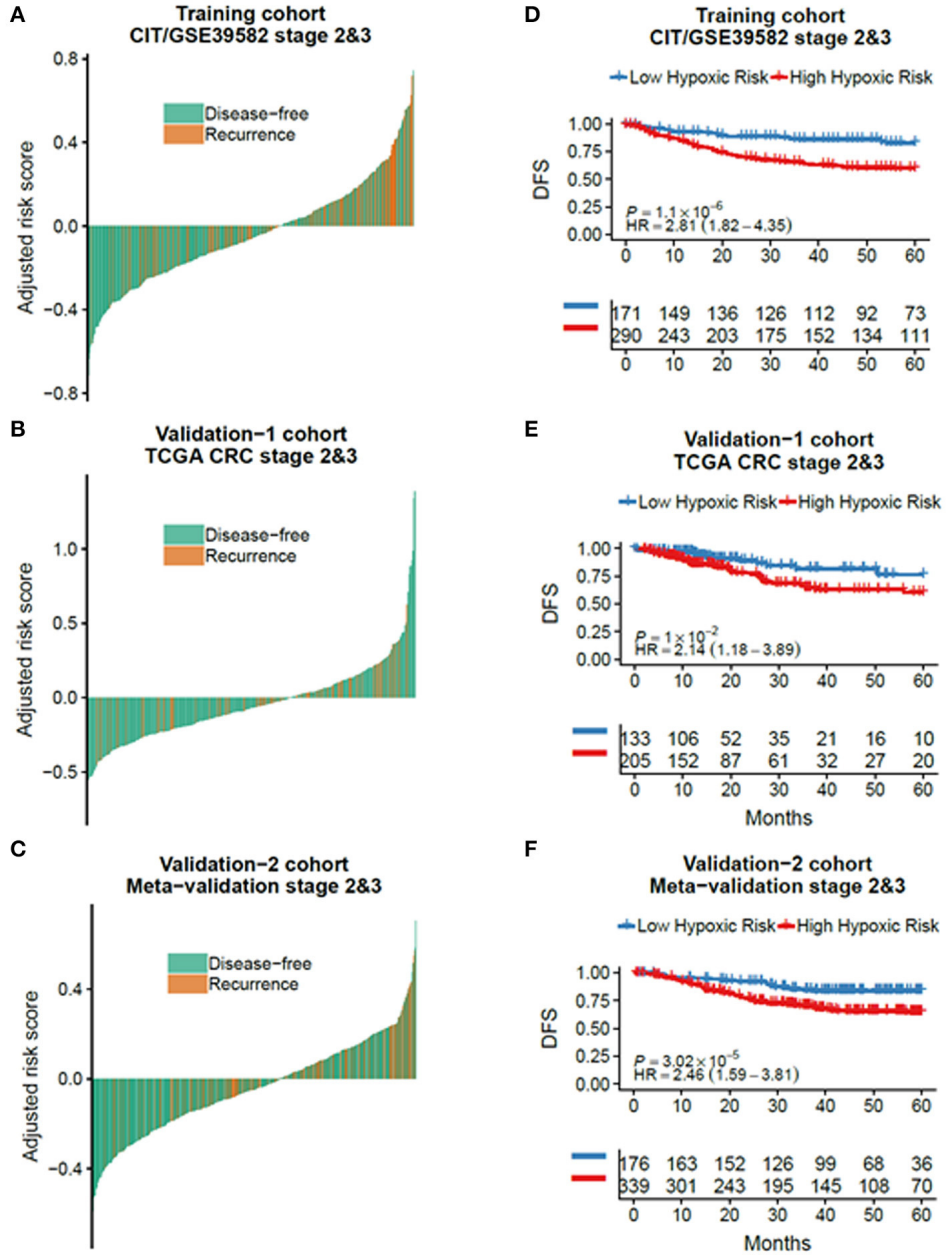
The outcome of low and high hypoxic risk in stage II/III CRC patients. The disease-free and recurrence patients in the different hypoxic risk groups of training cohort **(A)**, TCGA cohort **(B)**, and metavalidation cohort **(C)**. Kaplan–Meier curves comparing patients with low or high hypoxic risk in training cohort **(D)**, TCGA cohort, **(E)** and metavalidation cohort **(F)**. *P* values were calculated using log-rank tests. HR, hazard ratio.

According to the CIT gene microarray dataset (GSE39582), a total of 309 eligible CRC patients without chemotherapy were enrolled in the analysis as a discovery cohort. Among 3,444 HRGs selected from MSigDB, 3,184 HRGs were detected in the meta-analysis in this study. Filtering based on conditions that the median absolute deviation is >0.5 and expression level is greater than the total median expression level, 1,636 HRGs remained. Then, the HRGs were used as candidates to construct HRGS and stage II/III CRC patients in GSE39582 as training cohort. The association between these genes and patients' DFS were assessed using 1,000 times randomization of Cox univariate regression model, and choosing 95% repeatable genes, 40 HRGs were selected. Using LASSO Cox regression in stage II/III patients, 12 prognostic HRGs were selected to construct the HRGS (Figure 1). We used satisfactory RFS cutoff at 5 years in a time-dependent ROC curve analysis to train HRGS for stratifying high and low hypoxic risk groups (Supplementary Figure 1A). The prognosis correlation coefficient of each gene in HRGS was determined (Table 1), and the related risk score calculation model was defined (Supplementary Figures 1B,C). As expected, GSEA showed that the hypoxia pathways were enriched in the high hypoxic risk patients from GSE39582, confirming that the HRGS was hypoxic-related signature (Supplementary Figure 1D). Using stage II/III CRC patients in the CIT dataset (*n* = 469) as training dataset, more recurrence cases were arranged in the hypoxia high-risk group than in the low-risk group (Figures 2A,D; *P* < 0.001). Furthermore, this trend was confirmed in TCGA CRC cohort and Meta-validation cohort (Figures 2B,C,E,F).

**Table 1:** Twelve-Gene hypoxia signature.

**Gene**	**Function**	**Frequency in resampling**	**Average *P*-value**	**Coefficient**
TNFAIP8	TNF-α-induced protein	967	0.011	−0.006
ORAI3	Calcium release–activated modulator	999	0.003	0.052
MINPP1	Multiple inositol-polyphosphate phosphatase 1	1,000	0.001	−0.079
MBTD1	MBT domain containing	972	0.009	−0.023
TRAF3	TNF receptor–associated factor	958	0.012	−0.087
CYB5R3	Cytochrome b5 reductase	957	0.013	0.005
ZBTB44	Zinc finger and BTB domain containing	992	0.005	−0.050
CASP6	Caspase 6	998	0.003	−0.019
DTX3L	Deltex E3 ubiquitin ligase 3L	969	0.012	−0.003
FAM117B	Family with sequence similarity 117 member B	979	0.008	−0.059
PRELID2	PRELI domain containing	993	0.004	−0.023
IRF1	Interferon regulatory factor 1	992	0.007	−0.070

### Validating the HRGS Prognostic Capability in TCGA and Metavalidation Cohort

To validate the prognostic power of HRGS, the TCGA dataset (*n* = 624) and the metavalidation cohort (*n* = 687) composed of GSE17536, GSE33113, GSE37892, and GSE14333 were used. All datasets include the transcriptional data and prognostic information. No significant difference was observed among the three cohorts regarding the clinical and pathologic factors (Table 2, Supplementary Tables 1, 2). To evaluate the HRGS signature, we compared the nomogram with or without HRGS in R and analyzed the area under the curve (AUC) of the ROC curve. The AUC of nomogram with HRGS was 0.61, which was better than the nomogram without HRGS 0.56 (Supplementary Figures 2A,B). Decision curve analysis (DCA) was conducted to determine the clinical usefulness of the predicted nomogram by quantifying the net benefits at different threshold. As expected, addition of HRGS improved the DCA in patients of GSE39582, TCGA, and metavalidation cohorts (Supplementary Figure 2C).

**Table 2:** Univariate and multivariate analyses of HRGS and clinical and pathologic factors with DFS of stage II/III patients in training cohorts (CIT/GSE39582).

**Characteristic**	**Univariate**		**Multivariate**	
	**HR (95% CI)**	***P*-value**	**HR (95% CI)**	***P*-value**
HRGS	2.81(1.82–4.35)	<0.001	2.70(1.67–4.36)	<0.001
Age	1.01(1.00–1.02)	0.20		
Gender	1.25(0.88–1.77)	0.21		
TNM stage	1.95(1.38–2.75)	<0.001	1.48(1.03–2.14)	0.035
Tumor location	1.14(0.80–1.62)	0.46		
MMR status	1.98(1.04–3.77)	0.04		
CIMP status	0.67(0.38–1.19)	0.17		
CIN status	1.60(0.89–2.89)	0.11		
TP53 mutation	1.33(0.89–2.01)	0.17		
KRAS mutation	1.58(1.11–2.24)	0.01		
BRAF mutation	0.74(0.36–1.51)	0.40		

Using the same risk score calculation model, stage II/III CRC patients were divided into low and high hypoxic risk groups. Statistical differences of the DFS score were detected between these two risk groups in the training cohort [hazard ratio (HR) = 2.81, 95% confidence interval (CI) = 1.82–4.35, *P* < 0.001], validation (HR = 2.14, 95% CI = 1.18–3.89, *P* = 0.01), and metavalidation cohort (HR = 2.46, 95% CI = 1.59–3.81, *P* < 0.001) (Figures 2D–F). To further validate its clinical prognostic value, HRGS was compared with Oncotype DX, a commercially available diagnostic test that estimates the recurrence risk of the breast cancer (Supplementary Table 3). The result showed that HRGS has a better C-index in the training cohort (0.73 vs. 0.60), TCGA cohort (0.69 vs. 0.51), and the metavalidation cohort (0.72 vs. 0.67).

Furthermore, the HRGS test showed satisfactory prognostic value to evaluate patents' DFS in all-stage CRC cohort (CSE39582 cohort, HR = 2.30, 95% CI = 1.60–3.30, *P* < 0.001; TCGA cohort, HR = 2.10, 95% CI = 1.30–3.39, *P* = 0.01; metavalidation cohort, HR = 2.43, 95% CI = 1.59–3.70, *P* < 0.001) (Supplementary Figures 3D–F) and had a prognostic value to evaluate patents' overall survival (Supplementary Figures 3G–I).

### HRGS Predicts Benefit From Chemotherapy in Stage II/III CRC Patients

Univariate and multivariate analyses were applied to determine if the age, sex, tumor stage, tumor location, pathologic gene status, and HRGS were associated with CRC patients' outcomes. Univariate analysis data showed that high HRGS was significantly associated with worse prognosis in the training cohort (HR = 2.81, 95% CI = 1.82 – 4.35, *P* < 0.001, Table 2), TCGA cohort (HR = 2.11, 95% CI = 1.16–3.83, *P* = 0.01, Supplementary Table 2), and metavalidation cohort (HR = 2.46, 95% CI = 1.49 – 3.81, *P* < 0.001, Supplementary Table 2). In addition, multivariate analysis revealed that HRGS could be an independent prognostic factor in the (CSE39582 cohort, HR = 2.70, 95% CI = 1.67–4.36, *P* < 0.001, Table 2; TCGA cohort, HR = 2.02, 95% CI = 1.11–3.68, *P* = 0.02; metavalidation cohort, HR = 2.12, 95% CI = 1.36–3.29, *P* < 0.001, Supplementary Table 2).

**Figure 3:**
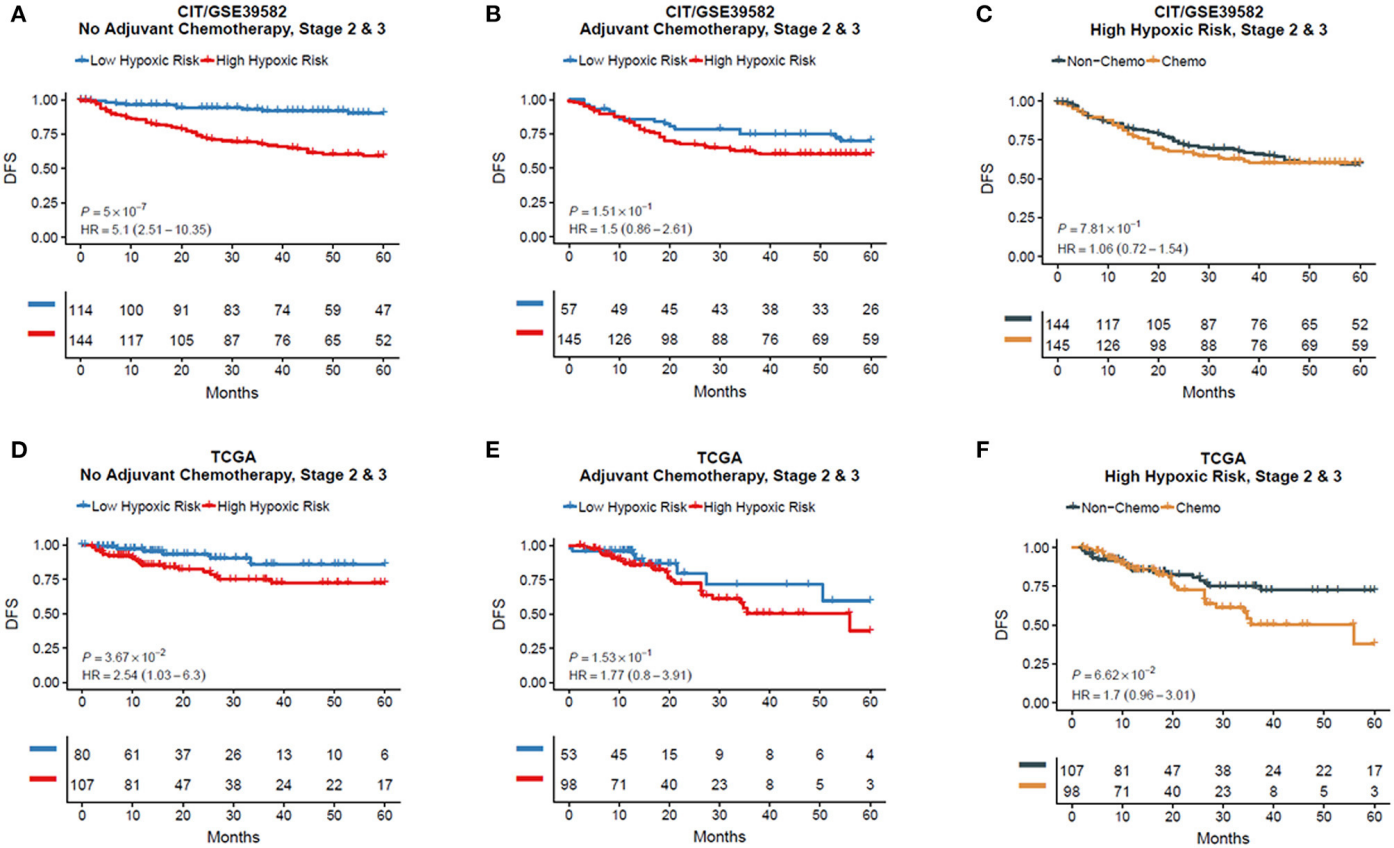
Kaplan–Meier plots for validations of the 12-gene hypoxia signature in chemotherapy and non-chemotherapy cohorts **(A–F)**.

To examine whether HRGS could predict the effect of adjuvant chemotherapy for CRC patients, we separated the non–chemotherapy-treated and the chemotherapy-treated group into high and low hypoxia groups using HRGS test and analyzed the prognosis data of these patient groups. In CRC patients without adjuvant chemotherapy, the DFS of the high hypoxia group was worse than that of the low hypoxia group in both the training (HR = 5.10, 95% CI = 2.51–10.35, *P* < 0.001, Figure 3A) and TCGA cohorts (HR = 2.54, 95% CI = 1.03–6.30, *P* = 0.037, Figure 3D). In patients who received adjuvant chemotherapy, no significant difference in DFS was found between the two groups (Figures 3B,E; *P* > 0.05). Moreover, the DFS of the high hypoxia group without chemotherapy treatment had a similar outcome as compared with the chemotherapy-treated patients (Figures 3C,F; *P* > 0.05).

### Functional Annotation of the HRGS

**Figure 4:**
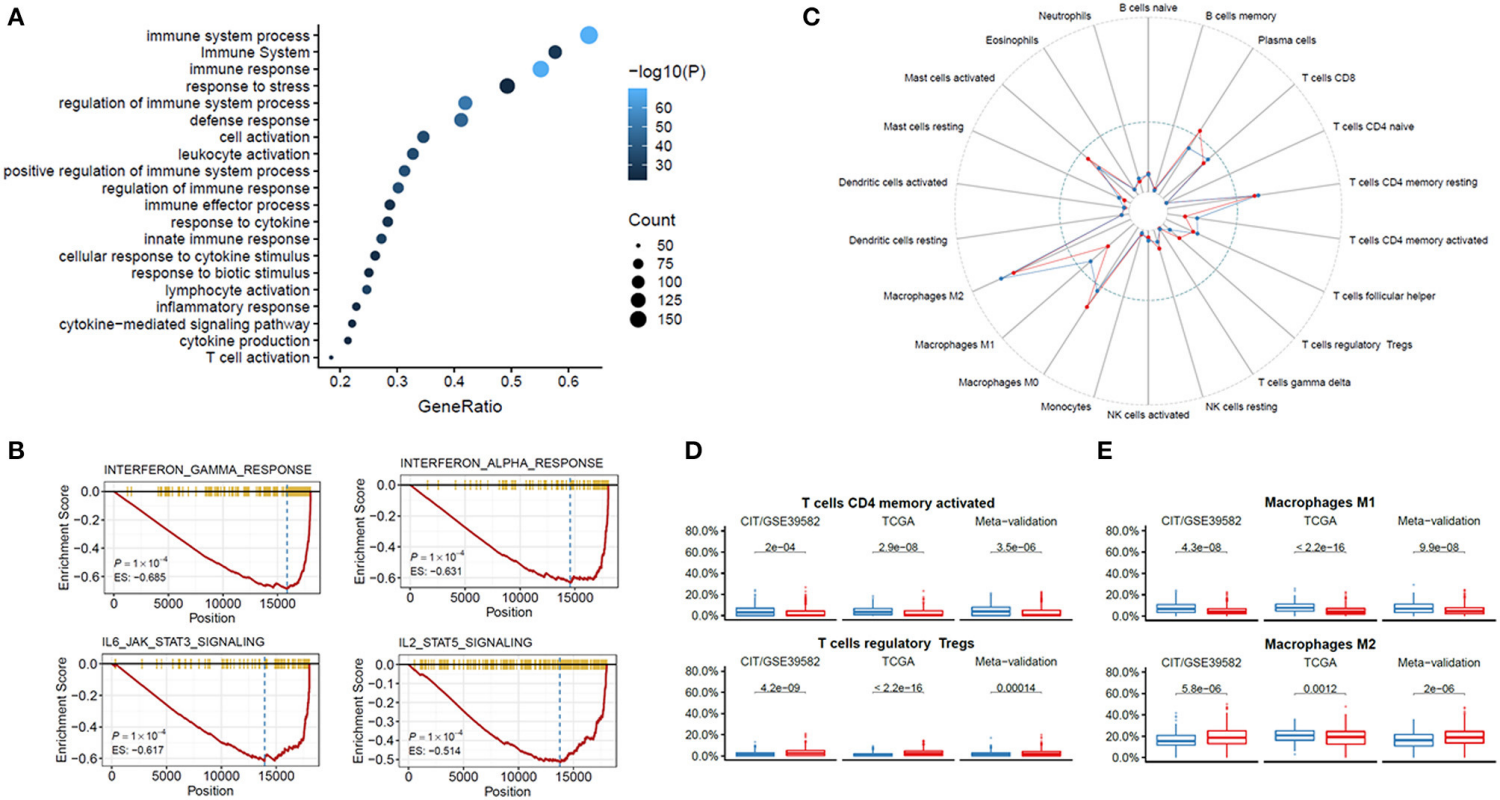
Functional annotation of the HRGS. Enrichment analysis of the differentially expressed genes between risk groups in GO and KEGG **(A)**. GSEA showed IFN-α, IFN-γ, IL-6, and IL-2 were depressed in the high hypoxic risk patients **(B)**. Immune cells were assessed based on data from TCGA **(C)**. CD4 T cells and M1 macrophages were depressed while T cells and M2 macrophages were enriched in the high hypoxia group **(D,E)**. *P* values comparing hypoxia high- and low-risk groups were calculated with *t* tests.

To explore the possible underlying mechanisms responsible for the worse outcome of DFS in the high hypoxic risk patients, enrichment analysis of differentially expressed genes was conducted by identifying several overrepresented biological processes in the Gene Ontology (GO) and Kyoto Encyclopedia of Genes and Genomes (KEGG). Interestingly, the most valuable biological processes were found to be associated with immune system response (Figure 4A). To further evaluate the role of genes corresponding to the HRGS, GSEA was performed in TCGA CRC cohort and found that the hypoxia environment was statistically related to interferon α (IFN-α), IFN-γ, interleukin 6 (IL-6), and IL-2 (Figure 4B), which are important components of the immune response network.

ESTIMATE algorithm was used to further validate the immune system response between different hypoxic risk patients. A lower stromal score (*P* < 0.001), immune score (*P* < 0.001), and ESTIMATE score (*P* < 0.001) were found in the high hypoxic risk group (Supplementary Figures 4A,B). Further analysis of the specific immune cell types were identified using CIBERSORT, lower percentages of CD4 T cells, and M1 macrophages, whereas higher percentages of regulatory T cells and M2 macrophages were detected in the high hypoxia group (Figures 4C,D). To investigate whether the HRGS can predict the prognosis of immunotherapy, we evaluated HRGS in a cohort of patients with programmed cell death (PD-1) blockade treatment (25). The results show that high hypoxic risk patients have a tendency for worse prognosis after immunotherapy but cannot reach statistical significance (Supplementary Figure 5A). In addition, high-risk group patients have a lower score of T-effector cell infiltration score (IMmotion150 Teff signature), immune infiltration (Javelin signature), and Merck18, which are consistent with our results (Supplementary Figure 5B).

To investigate the therapeutic strategy for high hypoxic risk patients, we analyzed the half maximal inhibitory concentration (IC_50_) of different drugs in the Pharmacogenomics database Genomics of Drug Sensitivity in Cancer (GDSC) with HRGS. Using the same risk score calculation model, all cell lines in the GDSC were divided into low and high hypoxic risk groups and analyzed the IC_50_ of different drugs. A total of five drugs have statistical significance: AZ6102, fulvestrant, irinotecan, temozolomide, and topotecan (Supplementary Figure 5C). As expected, high hypoxic risk group cells were more resistant to the traditional chemotherapeutic drugs, such as fulvestrant, irinotecan, temozolomide, and topotecan. Interestingly, high hypoxic risk group cells were more sensitive to AZ6102, a selective TNKS1/2 inhibitor.

## Discussion

Current cancer-related clinical trials have not included the hypoxia status as a variable factor despite large variability of tumor microenvironment due to the hypoxia status (26–29). Although hypoxia tumor microenvironment has been shown to affect the chemotherapy outcome in various cancer types (14, 30–32), there is no tool that could distinguish the high/low hypoxia risk and predict prognostic response to chemotherapy in CRC. In this study, we selected various HRGs to create an HRGS for CRC patients. The data suggested that the HRGS can stratify stage II/III CRC patients into subgroups with different DFS at a 5-year follow-up duration. Moreover, HRGS test showed a better C-index outcome as compared with the existing prognostic tool, Oncotype DX. These results indicate that the HRGS test could be an effective prognostic tool to distinguish the hypoxia status among CRC patients.

Current TNM stage classification could not efficiently separate the patient group for whom chemotherapy was effective within stage II CRC patients. Studies have shown that chemotherapy-treated patients have >5% improvement in the 5-year survival rate (2, 33), and the treatment did not reduce the recurrence risk (34–36). A more efficient selection method is required to determine the patient group for whom chemotherapy was effective within stage II CRC patients. A hypoxia gene signature–based test may be a potential candidate, as it is known that the hypoxia microenvironment could promote tumor invasion and metastasis (10, 11, 37), and HRGS could effectively select patients for individual-based treatment in other cancer types (7, 8). However, the predictive potential of HRGS for the disease prognosis and the chemotherapy outcome in CRC patients has not been examined yet. In this study, we identified high and low hypoxic risk groups within CRC patients using HRGS. The high hypoxic risk group within non–chemotherapy-treated CRC patients had a similar disease prognosis as compared to the chemotherapy-treated CRC patients and had a significantly worse prognosis as compared with low hypoxic risk group. These results implied that high hypoxic risk related to tumor recurrence and HRGS could help to determine the non–chemotherapy-treated patients who could benefit from chemotherapy or other adjuvant treatment.

To find a novel therapeutic strategy for high hypoxia risk patients, it is important to understand the intrinsic mechanism among hypoxia-related poor prognosis. Our previous study showed that the hypoxia tumor microenvironment was related to dysregulated cell cycle machinery and PI3K-AKT-mTOR pathways (38). To date, a genome-wide mechanistic analysis of how hypoxia tumor microenvironment induces poor chemotherapy response is still lacking. Analysis of an HRGS test identified that the hypoxia high-risk group has significantly lower scores of stromal and immune cell infiltrations than those observed in the hypoxia low-risk group. Previous studies have postulated that tumor hypoxia reduces the antitumor effect by suppressing the microenvironment immune response (37, 39). A large number of immunosuppressive cells, such as myeloid-derived suppressor cells, tumor-associated macrophages, and T-regulatory cells, were found in the hypoxic zones of the tumor (37, 40, 41). In line with these data, we also found that the enriched T-regulatory cells and M2 macrophages, but less amount of M1 macrophages in the high-hypoxic risk patients. These data suggest that hypoxia microenvironment was probably associated with immune suppression and enhanced tumor progression. As hypoxic microenvironment could provide tumor resistant to immunotherapy (42, 43), which has emerged as an effective therapy for CRC (44, 45), HRGS may have the potential to predict the outcome of the immunotherapy. Further studies will be required to verify this hypothesis.

Despite the exciting finding in this study, some limitations to the study should be noted. First, although we included as many datasets as possible for validation of our HRGS, the results of this study should be carefully evaluated because of the shortage of the retrospective design. Second, gene expression signature is subject to sampling bias caused by intratumor genetic heterogeneity (46). Although we have reduced as many as possible cross-study batch effects by constant ordering and excluding HRGs, their complex nature implies that not all batch effects can be addressed. To be more objective, further validation using prospective data from multiple centers would be ideal and necessary before its application in clinical practice.

In conclusion, the proposed prognostic HRGS is a promising test system to estimate the DFS of stage II/III CRC patients and to predict the possible beneficial effect from the chemotherapy. Hypoxia tumor microenvironment may promote chemoresistance in stage II/III CRC patients by inhibiting the local immune response.

## Data Availability Statement

The datasets generated for this study can be found in online repositories. The names of the repository/repositories and accession number(s) can be found in the article/Supplementary Material.

## Ethics Statement

The studies involving human participants were reviewed and approved by Institutional Review Board (IRB) of The Sixth Affiliated Hospital of Sun Yat-sen University. The patients/participants provided their written informed consent to participate in this study.

## Author Contributions

Y-fC, Z-lY, M-yL, PL, X-jW, and FG contributed to study concept and design, acquisition, analysis, interpretation of data, and drafting of the manuscript. Y-fC, Z-lY, BZ, X-hL, Z-rC, M-yL, and Y-xT contributed to data collections and manuscript review. Y-fC, Z-lY, Y-fZ, and JK contributed to study concept and design, analysis and interpretation of data, and critical revision of the manuscript for important intellectual content. PL, X-jW and FG supervised the study. All authors read and approved the final manuscript.

## Conflict of Interest

The authors declare that the research was conducted in the absence of any commercial or financial relationships that could be construed as a potential conflict of interest.

## Supplementary Material

Supplementary Figure 1 Obtain the optimal cutoff at 5 years in a time-dependent ROC curve analysis **(A)**. The risk score calculation model was defined: β is the prognosis correlation coefficient, and expr represents the expression value of the corresponding gene **(B)**. Heatmap of HRGs in the two groups **(C)**. GSEA showed hypoxia pathways were enriched in the high hypoxic risk patients **(D)**.

Supplementary Figure 2 Nomogram for evaluation of HRGS. Development of the nomogram based on the result of multivariate analysis **(A)**. The receiver operating characteristic (ROC) curve **(B)** and decision curve analysis (DCA) **(C)** of nomogram with or without HRGS.

Supplementary Figure 3 The clinical outcome of low and high hypoxic risk groups in all-stage CRC patients. The disease-free and recurrence patients in the different hypoxic risk groups of training cohort **(A)**, TCGA cohort **(B)**, and metavalidation cohort **(C)**. Kaplan–Meier curves comparing patients' DFS with low or high hypoxic risk in training cohort **(D)**, TCGA cohort **(E)**, and metavalidation cohort **(F)**. Kaplan–Meier curves comparing patients' OS with low or high hypoxic risk in training cohort **(G)**, TCGA cohort **(H)**, and metavalidation cohort **(I)**. *P* values were calculated using log-rank tests. HR, hazard ratio.

Supplementary Figure 4 Functional annotation of the HRGS. Heatmap of differentially expressed genes in the two groups **(A)**. Analysis of ESTIMATE algorithm to the TCGA dataset **(B)**.

Supplementary Figure 5 The overall survival of low and high hypoxic risk groups in advanced clear cell renal cell carcinoma patients with PD-1 blockade treatment **(A)**. The score of T-effector cell infiltration score (IMmotion150 Teff signature), immune infiltration (Javelin signature), and Merck18 in low and high hypoxic risk group patients. The IC_50_ of AZ6102, fulvestrant, irinotecan, temozolomide, and topotecan in low and high hypoxic risk group patients **(C)**.

Supplementary Table 1 Characteristics of II/III stage patients in training, validation and meta-validation cohorts.

Supplementary Table 2 Univariate and multivariate analysis of HRGs, clinical and pathologic factors of II/III stage patients in validation cohorts.

Supplementary Table 3 C-index for Hypoxic Risk compared with Oncotype DX in three cohorts.

Supplementary Table 4 Patients' hypoxic risk stratification.
