# Supplementary figures and images for: Genome-Wide Analysis Reveals Hypoxic Microenvironment Is Associated With Immunosuppression in Poor Survival of Stage II/III Colorectal Cancer Patients
Source: Front Med (Lausanne). 2021 Jun 15;8:686885. doi: 10.3389/fmed.2021.686885 (PMC8239145; doi:10.3389/fmed.2021.686885)

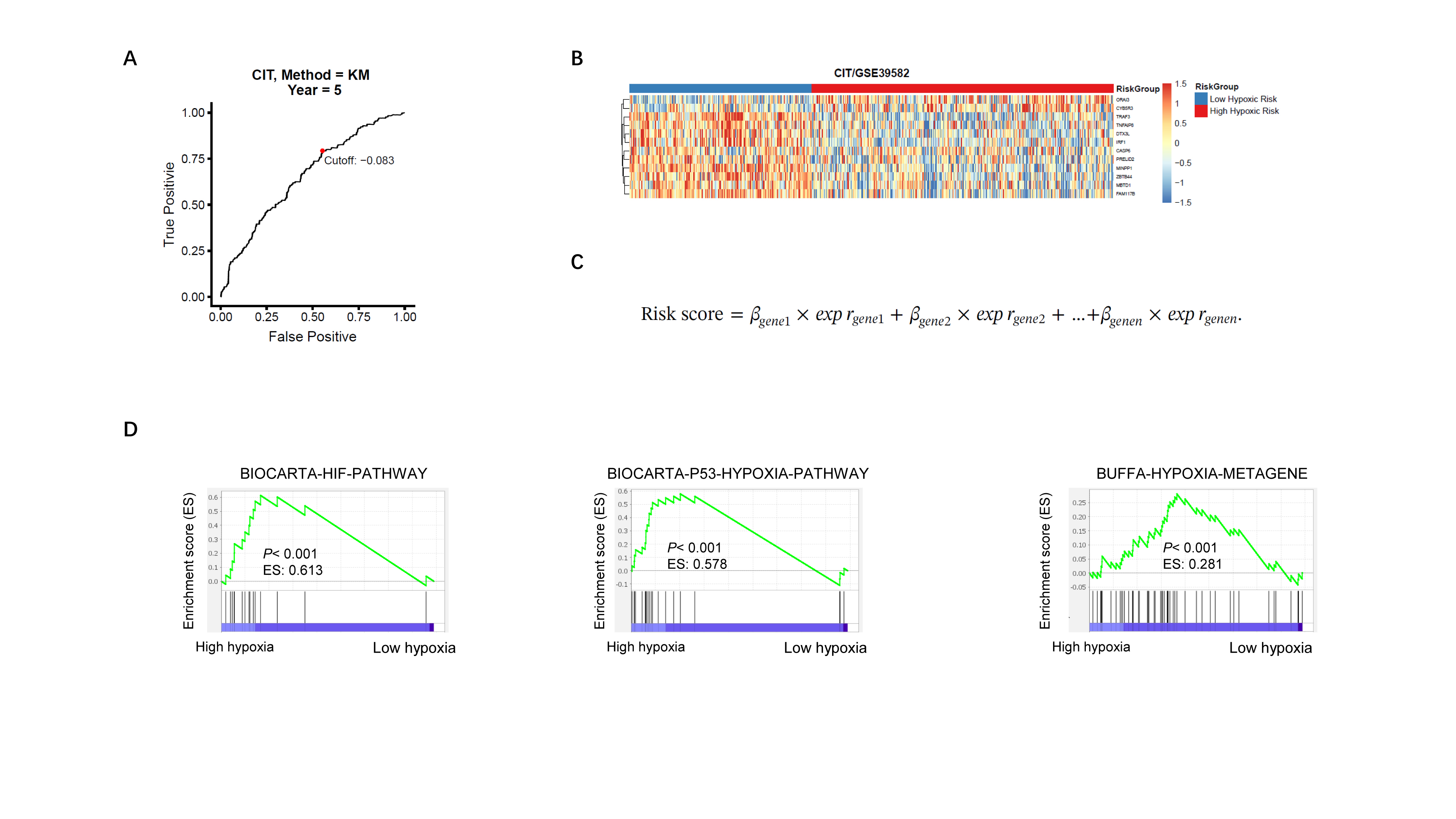

Supplement: Supplementary Figure 1 — Obtain the optimal cutoff at 5 years in a time-dependent ROC curve analysis (A). The risk score calculation model was defined: β is the prognosis correlation coefficient, and expr represents the expression value of the corresponding gene (B). Heatmap of HRGs in the two groups (C). GSEA showed hypoxia pathways were enriched in the high hypoxic risk patients (D). [file Image_1.JPEG]

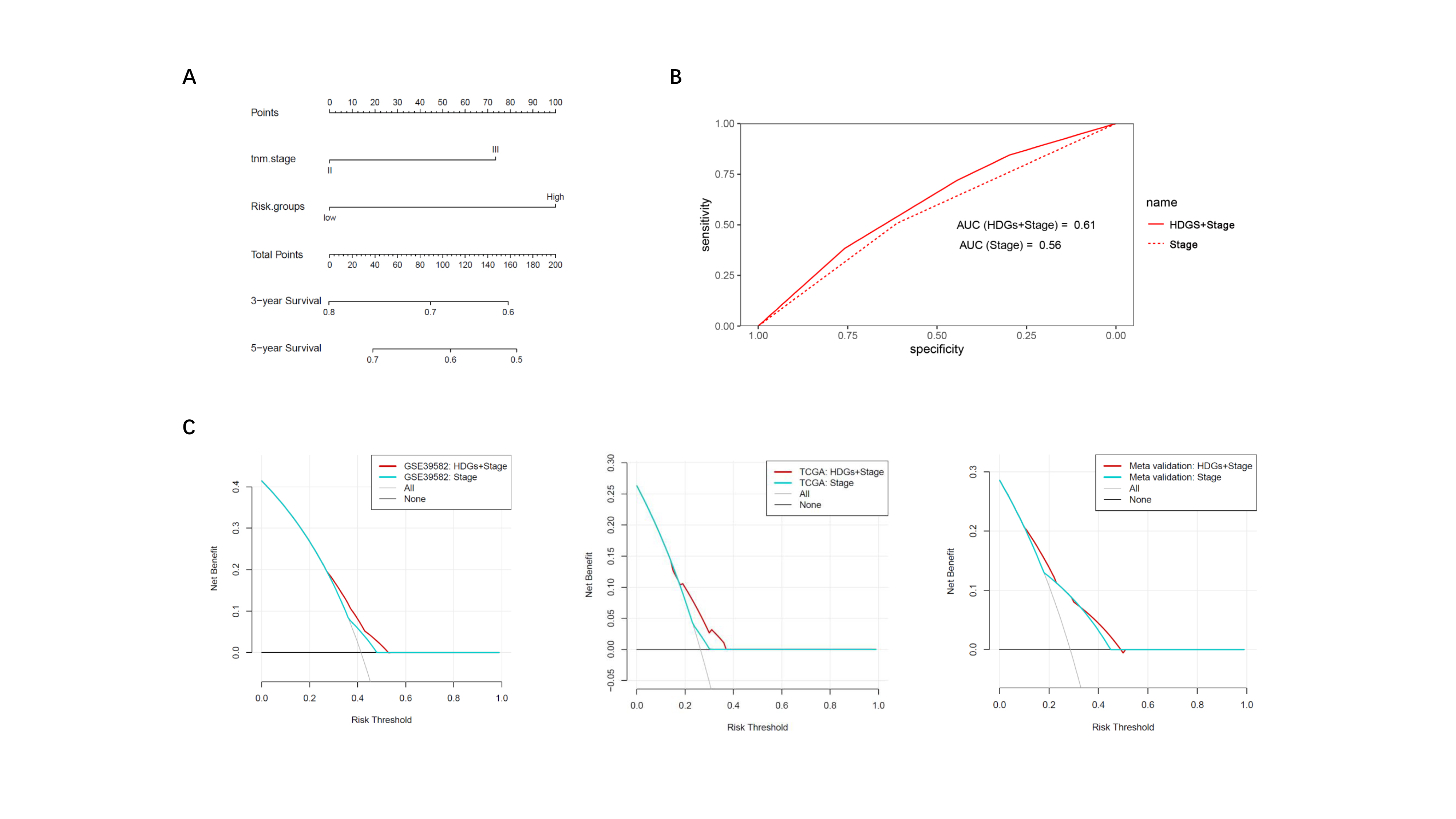

Supplement: Supplementary Figure 2 — Nomogram for evaluation of HRGS. Development of the nomogram based on the result of multivariate analysis (A). The receiver operating characteristic (ROC) curve (B) and decision curve analysis (DCA) (C) of nomogram with or without HRGS. [file Image_2.JPEG]

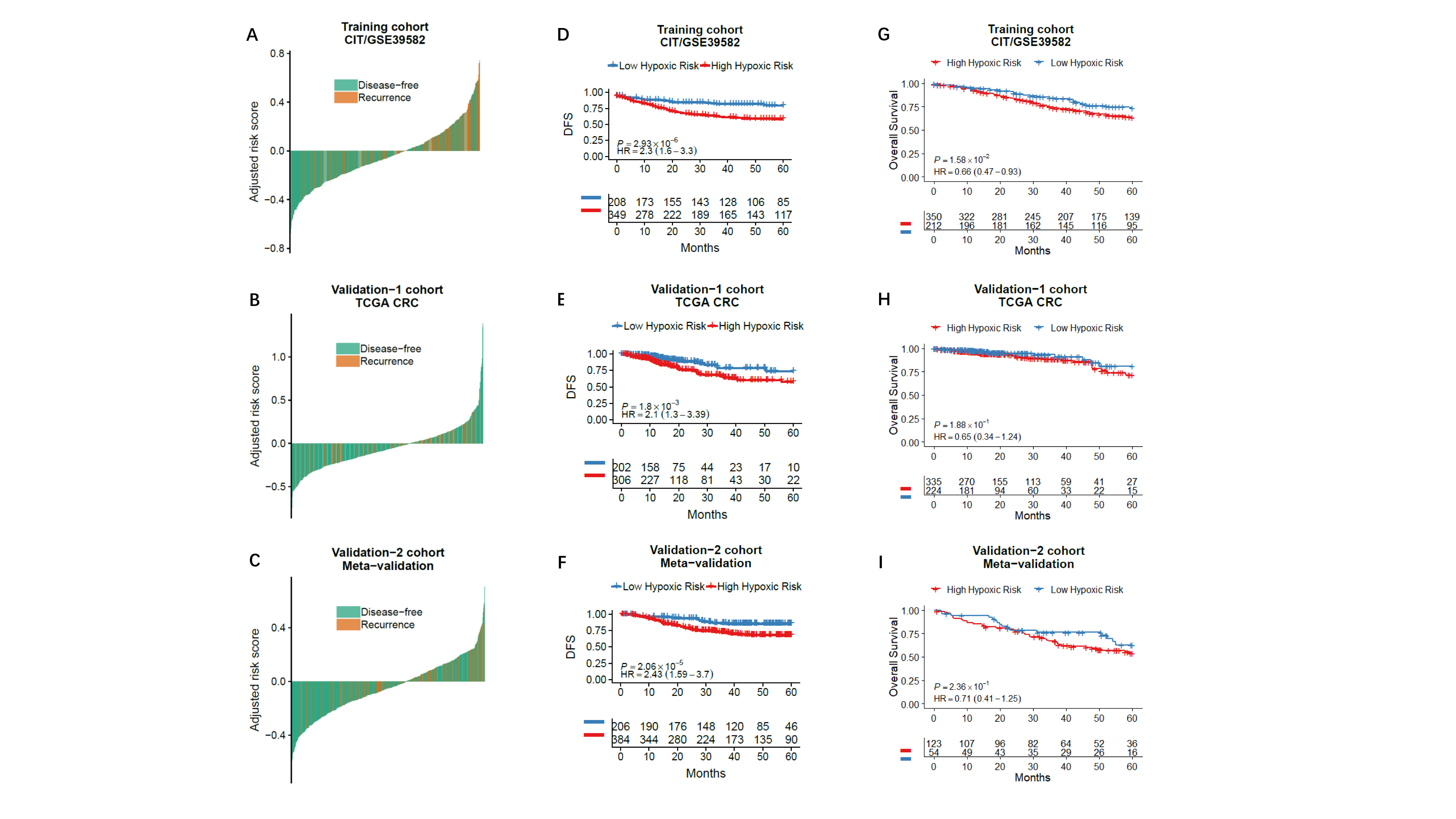

Supplement: Supplementary Figure 3 — The clinical outcome of low and high hypoxic risk groups in all-stage CRC patients. The disease-free and recurrence patients in the different hypoxic risk groups of training cohort (A), TCGA cohort (B), and metavalidation cohort (C). Kaplan–Meier curves comparing patients' DFS with low or high hypoxic risk in training cohort (D), TCGA cohort (E), and metavalidation cohort (F). Kaplan–Meier curves comparing patients' OS with low or high hypoxic risk in training cohort (G), TCGA cohort (H), and metavalidation cohort (I). P values were calculated using log-rank tests. HR, hazard ratio. [file Image_3.JPEG]

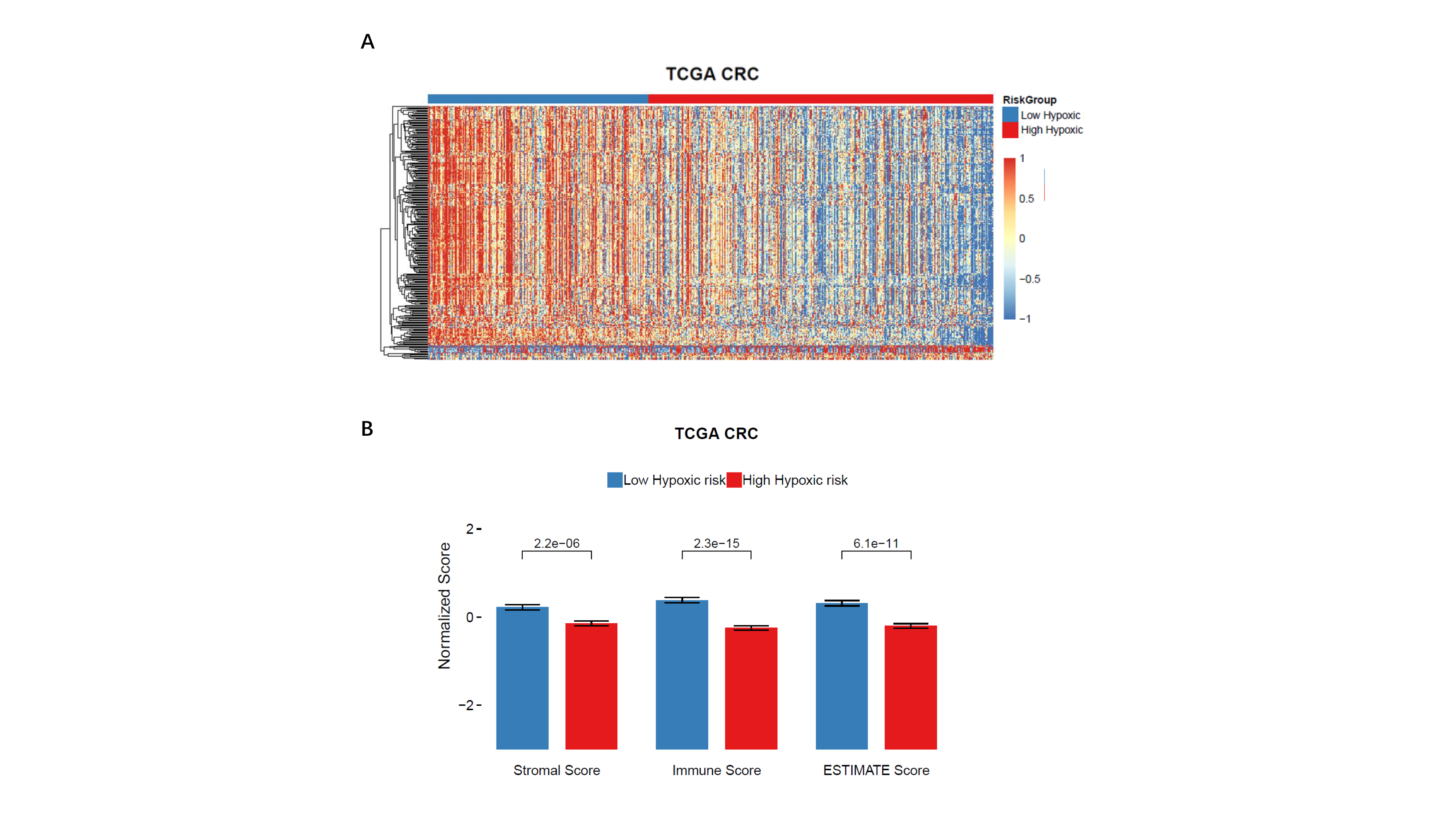

Supplement: Supplementary Figure 4 — Functional annotation of the HRGS. Heatmap of differentially expressed genes in the two groups (A). Analysis of ESTIMATE algorithm to the TCGA dataset (B). [file Image_4.JPEG]

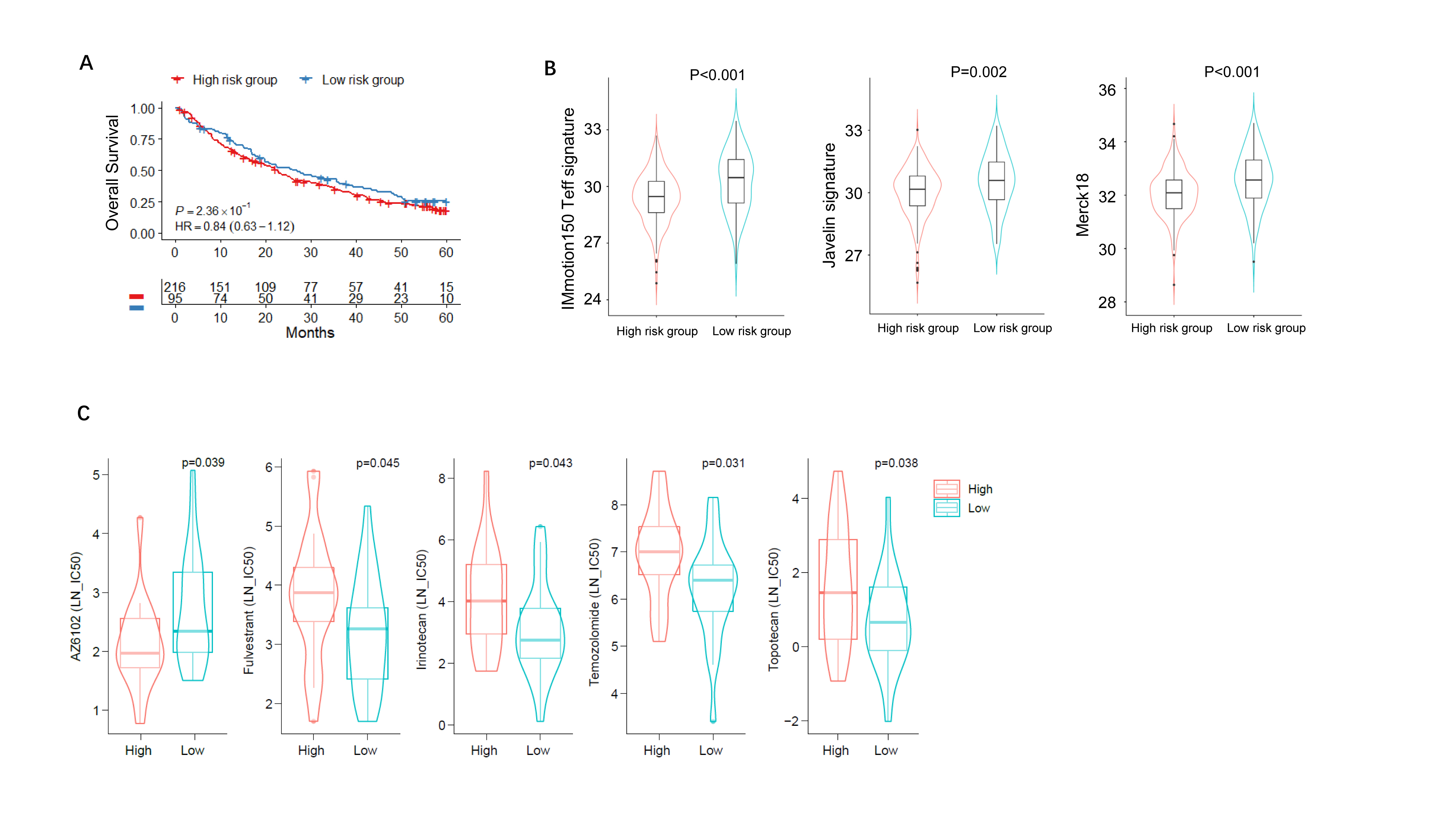

Supplement: Supplementary Figure 5 — The overall survival of low and high hypoxic risk groups in advanced clear cell renal cell carcinoma patients with PD-1 blockade treatment (A). The score of T-effector cell infiltration score (IMmotion150 Teff signature), immune infiltration (Javelin signature), and Merck18 in low and high hypoxic risk group patients. The IC50 of AZ6102, fulvestrant, irinotecan, temozolomide, and topotecan in low and high hypoxic risk group patients (C). [file Image_5.JPEG]
