# Supplementary material for: Genome-Wide Analysis Reveals Hypoxic Microenvironment Is Associated With Immunosuppression in Poor Survival of Stage II/III Colorectal Cancer Patients
Source: Front Med (Lausanne). 2021 Jun 15;8:686885. doi: 10.3389/fmed.2021.686885 (PMC8239145; doi:10.3389/fmed.2021.686885)
Supplement: Supplementary Table 1 — Characteristics of II/III stage patients in training, validation and meta-validation cohorts. [file Table_1.DOCX]

**Supplement Table 1.** Characteristics of II/III stage patients in training, validation and meta-validation cohorts.

| Characteristic | CIT/GSE39582 | TCGA | Meta-validation | |
| --- | --- | --- | --- | --- |
| Number of patients | 566 | 624 | | 687 |
| Patients with survival data | 557 | 509 | | 590 |
| Mean age, yrs | 66.85±13.29 | 66.27±12.76 | | 66.80±12.82 |
| Gender, n |  |  | |  |
| Male | 310 | 332 | | 371 |
| Femal | 256 | 292 | | 316 |
| T stage, n |  |  | |  |
| T1 | 12 | 21 | | NA |
| T2 | 45 | 105 | | NA |
| T3 | 367 | 425 | | 83 |
| T4 | 119 | 70 | | 7 |
| NA | 23 | 3 | | 597 |
| N stage, n |  |  | |  |
| N0 | 302 | 353 | | 90 |
| N1 | 134 | 150 | | NA |
| N2 | 98 | 116 | | NA |
| N3 | 6 | NA | | NA |
| NA | 26 | 5 | | 597 |
| M stage, n |  |  | |  |
| M0 | 482 | 353 | | 90 |
| M1 | 61 | 150 | | NA |
| NA | 23 | 121 | | 597 |
| TNM stage, n |  |  | |  |
| Stage Ⅰ | 33 | 105 | | 68 |
| Stage Ⅱ | 264 | 230 | | 314 |
| Stage Ⅲ | 205 | 180 | | 205 |
| Stage Ⅳ | 60 | 88 | | 100 |
| NA | 4 | 21 | | 0 |
| CMS system, n |  |  | |  |
| CMS1 | 91 | 68 | | 126 |
| CMS2 | 232 | 207 | | 252 |
| CMS3 | 69 | 64 | | 103 |
| CMS4 | 127 | 117 | | 155 |
| NA | 47 | 168 | | 51 |
| Tumor Location, n |  |  | |  |
| Left | 342 | 354 | | 233 |
| Right | 224 | 270 | | 185 |
| NA |  |  | | 269 |
| Adjuvant chemotherapy, n |  |  | |  |
| Yes | 233 | 231 | | 118 |
| No | 316 | 393 | | 171 |
| NA | 17 | 0 | | 398 |
| RFS event, n |  |  | |  |
| Yes | 177 | 100 | | 141 |
| No | 380 | 416 | | 449 |
| NA | 9 | 108 | | 97 |
| OS event, n |  |  | |  |
| Yes | 371 | 67 | | 73 |
| No | 191 | 557 | | 104 |
| NA | 4 | 0 | | 510 |
| DFS event, n |  |  | |  |
| Yes | 248 | 146 | | 188 |
| No | 314 | 386 | | 434 |
| NA | 4 | 92 | | 65 |
| MMR status, n |  |  | |  |
| MSI | 75 | 189 | | 25 |
| MSS | 444 | 431 | | 65 |
| NA | 47 | 4 | | 597 |
| CIMP status, n |  |  | |  |
| Positive | 91 | NA | | 26 |
| Negative | 405 | NA | | 64 |
| NA | 70 | 624 | | 597 |
| CIN status, n |  |  | |  |
| Positive | 353 | NA | | NA |
| Negative | 110 | NA | | NA |
| NA | 103 | 624 | | 687 |
| TP53 status, n |  |  | |  |
| wild type | 161 | NA | | NA |
| mutation | 190 | NA | | NA |
| NA | 215 | 624 | | 687 |
| KRAS status, n |  |  | |  |
| wild type | 328 | 34 | | 70 |
| mutation | 217 | 30 | | 20 |
| NA | 21 | 560 | | 597 |
| BRAF status, n |  |  | |  |
| wild type | 461 | 32 | | 73 |
| mutation | 51 | 3 | | 17 |
| NA | 54 | 589 | | 597 |
