# Supplementary material for: Genome-Wide Analysis Reveals Hypoxic Microenvironment Is Associated With Immunosuppression in Poor Survival of Stage II/III Colorectal Cancer Patients
Source: Front Med (Lausanne). 2021 Jun 15;8:686885. doi: 10.3389/fmed.2021.686885 (PMC8239145; doi:10.3389/fmed.2021.686885)
Supplement: Supplementary Table 2 — Univariate and multivariate analysis of HRGs, clinical and pathologic factors of II/III stage patients in validation cohorts. [file Table_2.DOCX]

**Supplement Table 2.** Univariate and multivariate analysis of HRGs, clinical and pathologic factors of II/III stage patients in validation cohorts.

| Characteristic | TCGA CRC | | | | |  | Meta-validation | | | | |
| --- | --- | --- | --- | --- | --- | --- | --- | --- | --- | --- | --- |
|  | Univariate | |  | Multivariate | |  | Univariate | |  | Multivariate | |
|  | HR (95%CI) | P-value |  | HR (95%CI) | P-value |  | HR (95%CI) | P-value |  | HR (95%CI) | P-value |
| HRGs | 2.11(1.16-3.83) | 0.01 |  | 2.02(1.11-3.68) | 0.02 |  | 2.46(1.59-3.81) | <0.001 |  | 2.12(1.36-3.29) | <0.001 |
| Age | 0.99(0.97-1.01) | 0.32 |  |  |  |  | 0.99(0.97-1.00) | 0.03 |  |  |  |
| Gender | 1.61(0.97-2.70) | 0.07 |  |  |  |  | 1.08(0.76-1.52) | 0.67 |  |  |  |
| TNM stage | 1.86(1.13-3.07) | 0.01 |  | 1.68(1.01-2.79) | 0.05 |  | 2.84(1.99-4.05) | <0.001 |  | 2.49(1.73-3.58) | <0.001 |
| Tumor Location | 0.81(0.49-1.32) | 0.39 |  |  |  |  | 1.11(0.72-1.73) | 0.63 |  |  |  |
| MMR status | 0.94(0.55-1.58) | 0.81 |  |  |  |  | 1.27(0.42-3.86) | 0.67 |  |  |  |
| CIMP status |  |  |  |  |  |  | 0.91(0.32-2.55) | 0.85 |  |  |  |
| CIN status |  |  |  |  |  |  |  |  |  |  |  |
| TP53 mutation |  |  |  |  |  |  |  |  |  |  |  |
| KRAS mutation | 0.66(0.21-2.03) | 0.16 |  |  |  |  | 1.40(0.50-3.94) | 0.52 |  |  |  |
| BRAF mutation |  |  |  |  |  |  | 1.72(0.61-4.81) | 0.30 |  |  |  |
