# Supplementary material for: Genome-Wide Analysis Reveals Hypoxic Microenvironment Is Associated With Immunosuppression in Poor Survival of Stage II/III Colorectal Cancer Patients
Source: Front Med (Lausanne). 2021 Jun 15;8:686885. doi: 10.3389/fmed.2021.686885 (PMC8239145; doi:10.3389/fmed.2021.686885)
Supplement: Supplementary Table 3 — C-index for Hypoxic Risk compared with Oncotype DX in three cohorts. [file Table_3.DOCX]

**Supplement Table 3.** C-index for Hypoxic Risk compared with Oncotype DX in three cohorts

| Cohorts | HRGS | |  | Oncotype DX | |
| --- | --- | --- | --- | --- | --- |
|  | C-index | 95%CI |  | C-index | 95%CI |
| CIT/GSE39582(training) | 0.73 | 0.65-0.82 |  | 0.60 | 0.52-0.68 |
| TCGA(validation) | 0.69 | 0.55-0.83 |  | 0.51 | 0.37-0.65 |
| Meta-validation | 0.72 | 0.63-0.81 |  | 0.67 | 0.60-0.73 |
