# Supplementary material for: Genome-Wide Analysis Reveals Hypoxic Microenvironment Is Associated With Immunosuppression in Poor Survival of Stage II/III Colorectal Cancer Patients
Source: Front Med (Lausanne). 2021 Jun 15;8:686885. doi: 10.3389/fmed.2021.686885 (PMC8239145; doi:10.3389/fmed.2021.686885)
Supplement: Supplementary Table 4 — Patients' hypoxic risk stratification. [file Table_4.DOCX]

**Supplement Table 4.** Patients’ hypoxic risk stratification

CIT/GSE39582 (n=566) TCGA (n=624) Meta-validation (n=687)

| ID | Risk score | Risk groups |  | ID | Risk score | Risk groups |  | ID | Risk score | Risk groups |
| --- | --- | --- | --- | --- | --- | --- | --- | --- | --- | --- |
| GSM971957 | -0.259302753 | Low Hypoxic Risk |  | TCGA-3L-AA1B-01 | -0.122900945 | Low Hypoxic Risk |  | GSM358341 | -0.106994699 | Low Hypoxic Risk |
| GSM971958 | 0.052522808 | High Hypoxic Risk |  | TCGA-4N-A93T-01 | 0.215136031 | High Hypoxic Risk |  | GSM358342 | 0.141355485 | High Hypoxic Risk |
| GSM971959 | 0.254412655 | High Hypoxic Risk |  | TCGA-4T-AA8H-01 | 0.189640536 | High Hypoxic Risk |  | GSM358343 | -0.313217391 | Low Hypoxic Risk |
| GSM971960 | -0.143387596 | Low Hypoxic Risk |  | TCGA-5M-AAT4-01 | -0.061493536 | High Hypoxic Risk |  | GSM358344 | 0.203070818 | High Hypoxic Risk |
| GSM971961 | 0.33803075 | High Hypoxic Risk |  | TCGA-5M-AAT6-01 | -0.19149951 | Low Hypoxic Risk |  | GSM358345 | 0.12533714 | High Hypoxic Risk |
| GSM971962 | -0.035701625 | High Hypoxic Risk |  | TCGA-5M-AATE-01 | 0.042803065 | High Hypoxic Risk |  | GSM358346 | 0.001861185 | High Hypoxic Risk |
| GSM971963 | -0.316512806 | Low Hypoxic Risk |  | TCGA-A6-2675-01 | -0.24932262 | Low Hypoxic Risk |  | GSM358347 | -0.106255432 | Low Hypoxic Risk |
| GSM971964 | -0.133778339 | Low Hypoxic Risk |  | TCGA-A6-2682-01 | 0.172690015 | High Hypoxic Risk |  | GSM358348 | 0.253575888 | High Hypoxic Risk |
| GSM971965 | 0.125582616 | High Hypoxic Risk |  | TCGA-A6-2684-01 | -0.01178546 | High Hypoxic Risk |  | GSM358349 | -0.070155506 | High Hypoxic Risk |
| GSM971966 | 0.105449152 | High Hypoxic Risk |  | TCGA-A6-2685-01 | 0.149996243 | High Hypoxic Risk |  | GSM358350 | -0.060757598 | High Hypoxic Risk |
| GSM971967 | -0.429993522 | Low Hypoxic Risk |  | TCGA-A6-2686-01 | 0.168412799 | High Hypoxic Risk |  | GSM358351 | -0.043422487 | High Hypoxic Risk |
| GSM971968 | 0.309036202 | High Hypoxic Risk |  | TCGA-A6-4105-01 | -0.186039705 | Low Hypoxic Risk |  | GSM358352 | 0.021397182 | High Hypoxic Risk |
| GSM971969 | -0.433310739 | Low Hypoxic Risk |  | TCGA-A6-5656-01 | 0.246782306 | High Hypoxic Risk |  | GSM358353 | -0.404693292 | Low Hypoxic Risk |
| GSM971970 | -0.129743999 | Low Hypoxic Risk |  | TCGA-A6-5657-01 | -0.127569965 | Low Hypoxic Risk |  | GSM358354 | -0.19996791 | Low Hypoxic Risk |
| GSM971971 | -0.30703544 | Low Hypoxic Risk |  | TCGA-A6-5659-01 | -0.098278144 | Low Hypoxic Risk |  | GSM358355 | -0.353862206 | Low Hypoxic Risk |
| GSM971972 | -0.093661973 | Low Hypoxic Risk |  | TCGA-A6-5660-01 | -0.010404724 | High Hypoxic Risk |  | GSM358356 | 0.015335487 | High Hypoxic Risk |
| GSM971973 | 0.168960252 | High Hypoxic Risk |  | TCGA-A6-5661-01 | -0.334931437 | Low Hypoxic Risk |  | GSM358357 | 0.159167215 | High Hypoxic Risk |
| GSM971974 | -0.165828959 | Low Hypoxic Risk |  | TCGA-A6-5662-01 | 0.045236693 | High Hypoxic Risk |  | GSM358358 | -0.1496904 | Low Hypoxic Risk |
| GSM971975 | -0.200859479 | Low Hypoxic Risk |  | TCGA-A6-5664-01 | -0.06941344 | High Hypoxic Risk |  | GSM358359 | 0.166631031 | High Hypoxic Risk |
| GSM971976 | -0.086409088 | Low Hypoxic Risk |  | TCGA-A6-5665-01 | -0.089609141 | Low Hypoxic Risk |  | GSM358360 | -0.089341526 | Low Hypoxic Risk |
| GSM971977 | -0.236530075 | Low Hypoxic Risk |  | TCGA-A6-5666-01 | 0.094500453 | High Hypoxic Risk |  | GSM358361 | -0.052122166 | High Hypoxic Risk |
| GSM971978 | -0.416336787 | Low Hypoxic Risk |  | TCGA-A6-5667-01 | -0.199372251 | Low Hypoxic Risk |  | GSM358362 | -0.491785213 | Low Hypoxic Risk |
| GSM971979 | 0.078731056 | High Hypoxic Risk |  | TCGA-A6-6137-01 | -0.378831349 | Low Hypoxic Risk |  | GSM358363 | -0.036698222 | High Hypoxic Risk |
| GSM971980 | 0.010130394 | High Hypoxic Risk |  | TCGA-A6-6138-01 | -0.250049083 | Low Hypoxic Risk |  | GSM358364 | 0.342200247 | High Hypoxic Risk |
| GSM971981 | -0.311225621 | Low Hypoxic Risk |  | TCGA-A6-6140-01 | -0.209775773 | Low Hypoxic Risk |  | GSM358365 | 0.16442286 | High Hypoxic Risk |
| GSM971982 | -0.36614383 | Low Hypoxic Risk |  | TCGA-A6-6141-01 | -0.176370757 | Low Hypoxic Risk |  | GSM358366 | 0.153935586 | High Hypoxic Risk |
| GSM971983 | -0.317900822 | Low Hypoxic Risk |  | TCGA-A6-6142-01 | 0.152811716 | High Hypoxic Risk |  | GSM358367 | 0.05312751 | High Hypoxic Risk |
| GSM971984 | -0.128894277 | Low Hypoxic Risk |  | TCGA-A6-6648-01 | -0.572463829 | Low Hypoxic Risk |  | GSM358368 | -0.069881858 | High Hypoxic Risk |
| GSM971985 | -0.248795504 | Low Hypoxic Risk |  | TCGA-A6-6649-01 | -0.152400182 | Low Hypoxic Risk |  | GSM358369 | -0.145018084 | Low Hypoxic Risk |
| GSM971986 | 0.616141024 | High Hypoxic Risk |  | TCGA-A6-6650-01 | -0.065356954 | High Hypoxic Risk |  | GSM358370 | 0.002206623 | High Hypoxic Risk |
| GSM971987 | -0.006102883 | High Hypoxic Risk |  | TCGA-A6-6651-01 | -0.044481957 | High Hypoxic Risk |  | GSM358371 | -0.095472121 | Low Hypoxic Risk |
| GSM971988 | 0.217752107 | High Hypoxic Risk |  | TCGA-A6-6652-01 | 0.008878939 | High Hypoxic Risk |  | GSM358372 | -0.304087147 | Low Hypoxic Risk |
| GSM971989 | -0.367670747 | Low Hypoxic Risk |  | TCGA-A6-6653-01 | 0.069279007 | High Hypoxic Risk |  | GSM358373 | -0.188313656 | Low Hypoxic Risk |
| GSM971990 | -0.368197454 | Low Hypoxic Risk |  | TCGA-A6-6654-01 | -0.047549429 | High Hypoxic Risk |  | GSM358374 | -0.347041989 | Low Hypoxic Risk |
| GSM971991 | -0.003964667 | High Hypoxic Risk |  | TCGA-A6-6780-01 | -0.130032832 | Low Hypoxic Risk |  | GSM358375 | -0.29945814 | Low Hypoxic Risk |
| GSM971992 | 0.071742537 | High Hypoxic Risk |  | TCGA-A6-6781-01 | 0.051163963 | High Hypoxic Risk |  | GSM358376 | -0.11971456 | Low Hypoxic Risk |
| GSM971993 | -0.588159257 | Low Hypoxic Risk |  | TCGA-A6-6782-01 | 0.16490046 | High Hypoxic Risk |  | GSM358377 | -0.354122506 | Low Hypoxic Risk |
| GSM971994 | 0.034851555 | High Hypoxic Risk |  | TCGA-A6-A565-01 | -0.164708634 | Low Hypoxic Risk |  | GSM358378 | -0.128942956 | Low Hypoxic Risk |
| GSM971995 | 0.085203284 | High Hypoxic Risk |  | TCGA-A6-A566-01 | -0.007230575 | High Hypoxic Risk |  | GSM358379 | -0.440659084 | Low Hypoxic Risk |
| GSM971996 | 0.342984262 | High Hypoxic Risk |  | TCGA-A6-A567-01 | 0.037914094 | High Hypoxic Risk |  | GSM358380 | -0.146420887 | Low Hypoxic Risk |
| GSM971997 | -0.218491169 | Low Hypoxic Risk |  | TCGA-A6-A56B-01 | 0.020508671 | High Hypoxic Risk |  | GSM358381 | -0.551883404 | Low Hypoxic Risk |
| GSM971998 | -0.01533649 | High Hypoxic Risk |  | TCGA-A6-A5ZU-01 | 0.301844491 | High Hypoxic Risk |  | GSM358382 | 0.066570518 | High Hypoxic Risk |
| GSM971999 | 0.159483752 | High Hypoxic Risk |  | TCGA-AA-3489-01 | -0.326956179 | Low Hypoxic Risk |  | GSM358383 | -0.238117411 | Low Hypoxic Risk |
| GSM972000 | 0.181259013 | High Hypoxic Risk |  | TCGA-AA-3492-01 | -0.208453588 | Low Hypoxic Risk |  | GSM358384 | 0.060706345 | High Hypoxic Risk |
| GSM972001 | -0.18650441 | Low Hypoxic Risk |  | TCGA-AA-3495-01 | -0.085932734 | Low Hypoxic Risk |  | GSM358385 | -0.171726051 | Low Hypoxic Risk |
| GSM972002 | 0.198301504 | High Hypoxic Risk |  | TCGA-AA-3496-01 | -0.288841559 | Low Hypoxic Risk |  | GSM358386 | 0.32050476 | High Hypoxic Risk |
| GSM972003 | -0.088179522 | Low Hypoxic Risk |  | TCGA-AA-3502-01 | 0.044836819 | High Hypoxic Risk |  | GSM358387 | 0.279729081 | High Hypoxic Risk |
| GSM972004 | 0.293982462 | High Hypoxic Risk |  | TCGA-AA-3506-01 | -0.045508656 | High Hypoxic Risk |  | GSM358388 | -0.029144325 | High Hypoxic Risk |
| GSM972005 | 0.153879662 | High Hypoxic Risk |  | TCGA-AA-3509-01 | -0.098957103 | Low Hypoxic Risk |  | GSM358389 | 0.12868509 | High Hypoxic Risk |
| GSM972006 | 0.04419157 | High Hypoxic Risk |  | TCGA-AA-3511-01 | 0.121768734 | High Hypoxic Risk |  | GSM358390 | 0.260690143 | High Hypoxic Risk |
| GSM972007 | 0.273464628 | High Hypoxic Risk |  | TCGA-AA-3526-01 | -0.207828469 | Low Hypoxic Risk |  | GSM358391 | 0.081419263 | High Hypoxic Risk |
| GSM972008 | 0.266153431 | High Hypoxic Risk |  | TCGA-AA-3655-01 | -0.257027679 | Low Hypoxic Risk |  | GSM358392 | 0.13735755 | High Hypoxic Risk |
| GSM972009 | 0.174270421 | High Hypoxic Risk |  | TCGA-AA-3660-01 | -0.047209194 | High Hypoxic Risk |  | GSM358393 | -0.073119496 | High Hypoxic Risk |
| GSM972010 | -0.182977574 | Low Hypoxic Risk |  | TCGA-AA-3662-01 | 0.125708686 | High Hypoxic Risk |  | GSM358394 | -0.034639827 | High Hypoxic Risk |
| GSM972011 | 0.075050719 | High Hypoxic Risk |  | TCGA-AA-3663-01 | -0.021060832 | High Hypoxic Risk |  | GSM358395 | 0.038600464 | High Hypoxic Risk |
| GSM972012 | -0.142892391 | Low Hypoxic Risk |  | TCGA-AA-3675-01 | 0.036294123 | High Hypoxic Risk |  | GSM358396 | 0.183358474 | High Hypoxic Risk |
| GSM972013 | -0.212006203 | Low Hypoxic Risk |  | TCGA-AA-3685-01 | -0.047911209 | High Hypoxic Risk |  | GSM358397 | 0.075252021 | High Hypoxic Risk |
| GSM972014 | -0.325158885 | Low Hypoxic Risk |  | TCGA-AA-3697-01 | -0.102151647 | Low Hypoxic Risk |  | GSM358398 | -0.155336914 | Low Hypoxic Risk |
| GSM972015 | 0.098036623 | High Hypoxic Risk |  | TCGA-AA-3712-01 | -0.176257275 | Low Hypoxic Risk |  | GSM358399 | 0.458155598 | High Hypoxic Risk |
| GSM972016 | -0.017163035 | High Hypoxic Risk |  | TCGA-AA-3713-01 | -0.707123132 | Low Hypoxic Risk |  | GSM358400 | 0.18330507 | High Hypoxic Risk |
| GSM972017 | 0.304386893 | High Hypoxic Risk |  | TCGA-AA-A01P-01 | -0.180433628 | Low Hypoxic Risk |  | GSM358401 | -0.171628766 | Low Hypoxic Risk |
| GSM972018 | -0.123637432 | Low Hypoxic Risk |  | TCGA-AA-A01X-01 | 0.148245231 | High Hypoxic Risk |  | GSM358402 | -0.051868837 | High Hypoxic Risk |
| GSM972019 | -0.197603699 | Low Hypoxic Risk |  | TCGA-AA-A01Z-01 | 0.157729437 | High Hypoxic Risk |  | GSM358403 | -0.137729226 | Low Hypoxic Risk |
| GSM972020 | -0.294841168 | Low Hypoxic Risk |  | TCGA-AA-A02K-01 | 0.209811138 | High Hypoxic Risk |  | GSM358404 | 0.054938204 | High Hypoxic Risk |
| GSM972021 | 0.169574689 | High Hypoxic Risk |  | TCGA-AA-A02Y-01 | 0.096607724 | High Hypoxic Risk |  | GSM358405 | 0.064449139 | High Hypoxic Risk |
| GSM972022 | 0.035487891 | High Hypoxic Risk |  | TCGA-AD-5900-01 | -0.106455646 | Low Hypoxic Risk |  | GSM358406 | -0.140963321 | Low Hypoxic Risk |
| GSM972023 | -0.033637663 | High Hypoxic Risk |  | TCGA-AD-6548-01 | -0.151325055 | Low Hypoxic Risk |  | GSM358407 | 0.178522314 | High Hypoxic Risk |
| GSM972024 | 0.098488753 | High Hypoxic Risk |  | TCGA-AD-6888-01 | 0.097324136 | High Hypoxic Risk |  | GSM358408 | -0.146222085 | Low Hypoxic Risk |
| GSM972025 | -0.072380414 | High Hypoxic Risk |  | TCGA-AD-6889-01 | -0.160401292 | Low Hypoxic Risk |  | GSM358409 | 0.299061575 | High Hypoxic Risk |
| GSM972026 | -0.015277097 | High Hypoxic Risk |  | TCGA-AD-6890-01 | 0.024746641 | High Hypoxic Risk |  | GSM358410 | -0.272988275 | Low Hypoxic Risk |
| GSM972027 | 0.266162889 | High Hypoxic Risk |  | TCGA-AD-6895-01 | -0.3298406 | Low Hypoxic Risk |  | GSM358411 | -0.076953747 | High Hypoxic Risk |
| GSM972028 | 0.17270489 | High Hypoxic Risk |  | TCGA-AD-6899-01 | -0.158045401 | Low Hypoxic Risk |  | GSM358412 | -0.171466856 | Low Hypoxic Risk |
| GSM972029 | 0.178450322 | High Hypoxic Risk |  | TCGA-AD-6901-01 | 0.088325238 | High Hypoxic Risk |  | GSM358413 | 0.025706624 | High Hypoxic Risk |
| GSM972030 | 0.248778628 | High Hypoxic Risk |  | TCGA-AD-6963-01 | -0.123590578 | Low Hypoxic Risk |  | GSM358414 | 0.291428384 | High Hypoxic Risk |
| GSM972031 | 0.153448294 | High Hypoxic Risk |  | TCGA-AD-6964-01 | -0.17728836 | Low Hypoxic Risk |  | GSM358415 | -0.100353152 | Low Hypoxic Risk |
| GSM972032 | -0.251872903 | Low Hypoxic Risk |  | TCGA-AD-6965-01 | -0.271559842 | Low Hypoxic Risk |  | GSM358416 | 0.100868924 | High Hypoxic Risk |
| GSM972033 | 0.0070504 | High Hypoxic Risk |  | TCGA-AD-A5EJ-01 | -0.061894337 | High Hypoxic Risk |  | GSM358417 | 0.208295034 | High Hypoxic Risk |
| GSM972034 | 0.086248182 | High Hypoxic Risk |  | TCGA-AD-A5EK-01 | -0.130538401 | Low Hypoxic Risk |  | GSM358418 | -0.301256687 | Low Hypoxic Risk |
| GSM972035 | -0.314861348 | Low Hypoxic Risk |  | TCGA-AM-5820-01 | 0.10658231 | High Hypoxic Risk |  | GSM358419 | -0.052582351 | High Hypoxic Risk |
| GSM972036 | 0.06696693 | High Hypoxic Risk |  | TCGA-AM-5821-01 | -0.121122576 | Low Hypoxic Risk |  | GSM358420 | 0.137795673 | High Hypoxic Risk |
| GSM972037 | 0.020841844 | High Hypoxic Risk |  | TCGA-AU-3779-01 | -0.001867856 | High Hypoxic Risk |  | GSM358421 | -0.006201707 | High Hypoxic Risk |
| GSM972038 | -0.257816746 | Low Hypoxic Risk |  | TCGA-AU-6004-01 | -0.298714562 | Low Hypoxic Risk |  | GSM358422 | -0.091724396 | Low Hypoxic Risk |
| GSM972039 | 0.224555821 | High Hypoxic Risk |  | TCGA-AY-5543-01 | -0.127784757 | Low Hypoxic Risk |  | GSM358423 | -0.329962074 | Low Hypoxic Risk |
| GSM972040 | -0.099368677 | Low Hypoxic Risk |  | TCGA-AY-6196-01 | -0.105882242 | Low Hypoxic Risk |  | GSM358424 | -0.089118686 | Low Hypoxic Risk |
| GSM972041 | 0.014753174 | High Hypoxic Risk |  | TCGA-AY-6197-01 | -0.340080721 | Low Hypoxic Risk |  | GSM358425 | 0.26537899 | High Hypoxic Risk |
| GSM972042 | -0.371893553 | Low Hypoxic Risk |  | TCGA-AY-6386-01 | -0.076069486 | High Hypoxic Risk |  | GSM358426 | 0.388608956 | High Hypoxic Risk |
| GSM972043 | -0.214453301 | Low Hypoxic Risk |  | TCGA-AY-A54L-01 | 0.145427326 | High Hypoxic Risk |  | GSM358427 | 0.049510919 | High Hypoxic Risk |
| GSM972044 | -0.072139299 | High Hypoxic Risk |  | TCGA-AY-A69D-01 | -0.277697903 | Low Hypoxic Risk |  | GSM358428 | 0.414837598 | High Hypoxic Risk |
| GSM972045 | -0.140169352 | Low Hypoxic Risk |  | TCGA-AY-A71X-01 | 0.094234463 | High Hypoxic Risk |  | GSM358429 | 0.006871081 | High Hypoxic Risk |
| GSM972046 | 0.144698651 | High Hypoxic Risk |  | TCGA-AY-A8YK-01 | -0.02169505 | High Hypoxic Risk |  | GSM358430 | -0.446467319 | Low Hypoxic Risk |
| GSM972047 | -0.096641889 | Low Hypoxic Risk |  | TCGA-AZ-4313-01 | 0.164727526 | High Hypoxic Risk |  | GSM358431 | 0.186529855 | High Hypoxic Risk |
| GSM972048 | -0.045179845 | High Hypoxic Risk |  | TCGA-AZ-4315-01 | -0.015540845 | High Hypoxic Risk |  | GSM358432 | -0.203744531 | Low Hypoxic Risk |
| GSM972049 | -0.095538355 | Low Hypoxic Risk |  | TCGA-AZ-4323-01 | -0.187740196 | Low Hypoxic Risk |  | GSM358433 | 0.278225641 | High Hypoxic Risk |
| GSM972050 | -0.149754805 | Low Hypoxic Risk |  | TCGA-AZ-4614-01 | -0.160215941 | Low Hypoxic Risk |  | GSM358434 | 0.132403089 | High Hypoxic Risk |
| GSM972051 | 0.025785774 | High Hypoxic Risk |  | TCGA-AZ-4615-01 | -0.255255112 | Low Hypoxic Risk |  | GSM358435 | 0.079759274 | High Hypoxic Risk |
| GSM972052 | -0.293479935 | Low Hypoxic Risk |  | TCGA-AZ-4616-01 | 0.262889312 | High Hypoxic Risk |  | GSM358436 | 0.407932673 | High Hypoxic Risk |
| GSM972053 | -0.330666617 | Low Hypoxic Risk |  | TCGA-AZ-4682-01 | 0.257245789 | High Hypoxic Risk |  | GSM358437 | -0.163023026 | Low Hypoxic Risk |
| GSM972054 | -0.057216078 | High Hypoxic Risk |  | TCGA-AZ-4684-01 | 0.006924075 | High Hypoxic Risk |  | GSM358438 | 0.429303014 | High Hypoxic Risk |
| GSM972055 | 0.074320194 | High Hypoxic Risk |  | TCGA-AZ-5403-01 | 0.107143041 | High Hypoxic Risk |  | GSM358439 | -0.059532787 | High Hypoxic Risk |
| GSM972056 | 0.334886253 | High Hypoxic Risk |  | TCGA-AZ-5407-01 | 0.077730121 | High Hypoxic Risk |  | GSM358440 | -0.09086011 | Low Hypoxic Risk |
| GSM972057 | 0.354298687 | High Hypoxic Risk |  | TCGA-AZ-6598-01 | -0.262198293 | Low Hypoxic Risk |  | GSM358441 | 0.050257673 | High Hypoxic Risk |
| GSM972058 | -0.075261035 | High Hypoxic Risk |  | TCGA-AZ-6599-01 | -0.033962776 | High Hypoxic Risk |  | GSM358442 | 0.145570072 | High Hypoxic Risk |
| GSM972059 | 0.621105122 | High Hypoxic Risk |  | TCGA-AZ-6600-01 | 0.022874336 | High Hypoxic Risk |  | GSM358443 | -0.248807042 | Low Hypoxic Risk |
| GSM972060 | -0.157872123 | Low Hypoxic Risk |  | TCGA-AZ-6601-01 | -0.400627151 | Low Hypoxic Risk |  | GSM358444 | -0.124858871 | Low Hypoxic Risk |
| GSM972061 | 0.604350331 | High Hypoxic Risk |  | TCGA-AZ-6603-01 | -0.208170344 | Low Hypoxic Risk |  | GSM358445 | -0.392631684 | Low Hypoxic Risk |
| GSM972062 | -0.058069517 | High Hypoxic Risk |  | TCGA-AZ-6605-01 | -0.045741196 | High Hypoxic Risk |  | GSM358446 | 0.181869073 | High Hypoxic Risk |
| GSM972063 | -0.254890477 | Low Hypoxic Risk |  | TCGA-AZ-6606-01 | -0.154807 | Low Hypoxic Risk |  | GSM358447 | -0.22503984 | Low Hypoxic Risk |
| GSM972064 | -0.025251155 | High Hypoxic Risk |  | TCGA-AZ-6607-01 | -0.163307551 | Low Hypoxic Risk |  | GSM358448 | 0.110448696 | High Hypoxic Risk |
| GSM972065 | -0.127087729 | Low Hypoxic Risk |  | TCGA-AZ-6608-01 | 0.207606242 | High Hypoxic Risk |  | GSM358449 | -0.044989196 | High Hypoxic Risk |
| GSM972066 | -0.105567425 | Low Hypoxic Risk |  | TCGA-CA-5254-01 | 0.055347 | High Hypoxic Risk |  | GSM358450 | -0.335221261 | Low Hypoxic Risk |
| GSM972067 | 0.046090037 | High Hypoxic Risk |  | TCGA-CA-5255-01 | 0.482471224 | High Hypoxic Risk |  | GSM358451 | -0.079096232 | High Hypoxic Risk |
| GSM972068 | 0.115936106 | High Hypoxic Risk |  | TCGA-CA-5256-01 | 0.177942547 | High Hypoxic Risk |  | GSM358452 | -0.301138721 | Low Hypoxic Risk |
| GSM972069 | -0.129384063 | Low Hypoxic Risk |  | TCGA-CA-5796-01 | -0.182222252 | Low Hypoxic Risk |  | GSM358453 | -0.43556723 | Low Hypoxic Risk |
| GSM972070 | -0.175287978 | Low Hypoxic Risk |  | TCGA-CA-5797-01 | -0.370096171 | Low Hypoxic Risk |  | GSM358454 | 0.216028902 | High Hypoxic Risk |
| GSM972071 | -0.254877488 | Low Hypoxic Risk |  | TCGA-CA-6715-01 | -0.0656137 | High Hypoxic Risk |  | GSM358455 | -0.164087749 | Low Hypoxic Risk |
| GSM972072 | -0.163842476 | Low Hypoxic Risk |  | TCGA-CA-6716-01 | -0.023342134 | High Hypoxic Risk |  | GSM358456 | -0.146445372 | Low Hypoxic Risk |
| GSM972073 | -0.089197374 | Low Hypoxic Risk |  | TCGA-CA-6717-01 | -0.279058149 | Low Hypoxic Risk |  | GSM358457 | -0.088317486 | Low Hypoxic Risk |
| GSM972074 | -0.055387762 | High Hypoxic Risk |  | TCGA-CA-6718-01 | -0.374361262 | Low Hypoxic Risk |  | GSM358458 | -0.088203789 | Low Hypoxic Risk |
| GSM972075 | -0.331064328 | Low Hypoxic Risk |  | TCGA-CA-6719-01 | -0.03970547 | High Hypoxic Risk |  | GSM358459 | 0.127303434 | High Hypoxic Risk |
| GSM972076 | -0.242892393 | Low Hypoxic Risk |  | TCGA-CK-4947-01 | -0.336229727 | Low Hypoxic Risk |  | GSM358460 | -0.054453517 | High Hypoxic Risk |
| GSM972077 | 0.763504566 | High Hypoxic Risk |  | TCGA-CK-4948-01 | -0.078250004 | High Hypoxic Risk |  | GSM358461 | -0.181054118 | Low Hypoxic Risk |
| GSM972078 | -0.241571 | Low Hypoxic Risk |  | TCGA-CK-4950-01 | -0.073854745 | High Hypoxic Risk |  | GSM358462 | -0.177945616 | Low Hypoxic Risk |
| GSM972079 | 0.619941523 | High Hypoxic Risk |  | TCGA-CK-4951-01 | -0.314822589 | Low Hypoxic Risk |  | GSM358463 | -0.209056289 | Low Hypoxic Risk |
| GSM972080 | -0.259166046 | Low Hypoxic Risk |  | TCGA-CK-4952-01 | -0.20244553 | Low Hypoxic Risk |  | GSM358464 | 0.010787999 | High Hypoxic Risk |
| GSM972081 | -0.06557062 | High Hypoxic Risk |  | TCGA-CK-5912-01 | -0.179720959 | Low Hypoxic Risk |  | GSM358465 | -0.206839213 | Low Hypoxic Risk |
| GSM972082 | 0.090845667 | High Hypoxic Risk |  | TCGA-CK-5913-01 | -0.236332503 | Low Hypoxic Risk |  | GSM358466 | -0.04442815 | High Hypoxic Risk |
| GSM972083 | -0.065711981 | High Hypoxic Risk |  | TCGA-CK-5914-01 | -0.321120349 | Low Hypoxic Risk |  | GSM358467 | 0.390521819 | High Hypoxic Risk |
| GSM972084 | -0.159474968 | Low Hypoxic Risk |  | TCGA-CK-5915-01 | -0.5638452 | Low Hypoxic Risk |  | GSM358468 | -0.249411893 | Low Hypoxic Risk |
| GSM972085 | -0.062490248 | High Hypoxic Risk |  | TCGA-CK-5916-01 | -0.467708512 | Low Hypoxic Risk |  | GSM358469 | 0.277493247 | High Hypoxic Risk |
| GSM972086 | -0.475201747 | Low Hypoxic Risk |  | TCGA-CK-6746-01 | -0.092063338 | Low Hypoxic Risk |  | GSM358470 | -0.066604374 | High Hypoxic Risk |
| GSM972087 | -0.04898929 | High Hypoxic Risk |  | TCGA-CK-6747-01 | -0.285015963 | Low Hypoxic Risk |  | GSM358471 | -0.118977038 | Low Hypoxic Risk |
| GSM972088 | -0.082944914 | Low Hypoxic Risk |  | TCGA-CK-6748-01 | 0.221408426 | High Hypoxic Risk |  | GSM358472 | -0.00972353 | High Hypoxic Risk |
| GSM972089 | 0.090713714 | High Hypoxic Risk |  | TCGA-CK-6751-01 | -0.057988206 | High Hypoxic Risk |  | GSM358473 | -0.021364072 | High Hypoxic Risk |
| GSM972090 | -0.02917974 | High Hypoxic Risk |  | TCGA-CM-4743-01 | 0.142785612 | High Hypoxic Risk |  | GSM358474 | 0.104861496 | High Hypoxic Risk |
| GSM972091 | -0.07213975 | High Hypoxic Risk |  | TCGA-CM-4744-01 | 0.138073468 | High Hypoxic Risk |  | GSM358475 | -0.113761404 | Low Hypoxic Risk |
| GSM972092 | -0.280823233 | Low Hypoxic Risk |  | TCGA-CM-4747-01 | 0.124199633 | High Hypoxic Risk |  | GSM358476 | -0.082978348 | Low Hypoxic Risk |
| GSM972093 | -0.0668229 | High Hypoxic Risk |  | TCGA-CM-4751-01 | -0.300666538 | Low Hypoxic Risk |  | GSM358477 | -0.234450657 | Low Hypoxic Risk |
| GSM972094 | -0.096180475 | Low Hypoxic Risk |  | TCGA-CM-5344-01 | 0.224189256 | High Hypoxic Risk |  | GSM358478 | 0.130799226 | High Hypoxic Risk |
| GSM972095 | 0.013925363 | High Hypoxic Risk |  | TCGA-CM-5348-01 | 0.005368172 | High Hypoxic Risk |  | GSM358479 | 0.196784406 | High Hypoxic Risk |
| GSM972096 | 0.250876149 | High Hypoxic Risk |  | TCGA-CM-5349-01 | 0.287791222 | High Hypoxic Risk |  | GSM358480 | -0.034272024 | High Hypoxic Risk |
| GSM972097 | 0.767554656 | High Hypoxic Risk |  | TCGA-CM-5860-01 | -0.109086674 | Low Hypoxic Risk |  | GSM358481 | 0.231663473 | High Hypoxic Risk |
| GSM972098 | 0.024054822 | High Hypoxic Risk |  | TCGA-CM-5861-01 | 0.090879216 | High Hypoxic Risk |  | GSM358482 | 0.061325667 | High Hypoxic Risk |
| GSM972099 | 0.087028412 | High Hypoxic Risk |  | TCGA-CM-5862-01 | 0.12422376 | High Hypoxic Risk |  | GSM358483 | -0.004947097 | High Hypoxic Risk |
| GSM972100 | 0.196518104 | High Hypoxic Risk |  | TCGA-CM-5863-01 | 0.121699388 | High Hypoxic Risk |  | GSM358484 | -0.043873359 | High Hypoxic Risk |
| GSM972101 | 0.379413523 | High Hypoxic Risk |  | TCGA-CM-5864-01 | 0.10926726 | High Hypoxic Risk |  | GSM358485 | -0.067479227 | High Hypoxic Risk |
| GSM972102 | 0.152979123 | High Hypoxic Risk |  | TCGA-CM-5868-01 | 0.117113602 | High Hypoxic Risk |  | GSM358486 | 0.474981764 | High Hypoxic Risk |
| GSM972103 | -0.07866771 | High Hypoxic Risk |  | TCGA-CM-6161-01 | -0.334298125 | Low Hypoxic Risk |  | GSM358487 | -0.12977896 | Low Hypoxic Risk |
| GSM972104 | 0.058772348 | High Hypoxic Risk |  | TCGA-CM-6162-01 | -0.355115949 | Low Hypoxic Risk |  | GSM358488 | 0.282453764 | High Hypoxic Risk |
| GSM972105 | 0.5289077 | High Hypoxic Risk |  | TCGA-CM-6163-01 | -0.124081933 | Low Hypoxic Risk |  | GSM358489 | -0.057659664 | High Hypoxic Risk |
| GSM972106 | -0.032209592 | High Hypoxic Risk |  | TCGA-CM-6164-01 | -0.224690139 | Low Hypoxic Risk |  | GSM358490 | 0.009846081 | High Hypoxic Risk |
| GSM972107 | 0.220423563 | High Hypoxic Risk |  | TCGA-CM-6165-01 | -0.489689787 | Low Hypoxic Risk |  | GSM358491 | 0.178454038 | High Hypoxic Risk |
| GSM972108 | -0.029755811 | High Hypoxic Risk |  | TCGA-CM-6166-01 | 0.034540138 | High Hypoxic Risk |  | GSM358492 | -0.116736718 | Low Hypoxic Risk |
| GSM972109 | 0.084414737 | High Hypoxic Risk |  | TCGA-CM-6167-01 | -0.029665347 | High Hypoxic Risk |  | GSM358493 | 0.244563286 | High Hypoxic Risk |
| GSM972110 | 0.034524077 | High Hypoxic Risk |  | TCGA-CM-6168-01 | -0.169353337 | Low Hypoxic Risk |  | GSM358494 | 0.082471067 | High Hypoxic Risk |
| GSM972111 | 0.43723526 | High Hypoxic Risk |  | TCGA-CM-6169-01 | -0.41814698 | Low Hypoxic Risk |  | GSM358495 | -0.186010775 | Low Hypoxic Risk |
| GSM972112 | 0.541361586 | High Hypoxic Risk |  | TCGA-CM-6170-01 | -0.135775609 | Low Hypoxic Risk |  | GSM358496 | -0.052801756 | High Hypoxic Risk |
| GSM972113 | 0.510487272 | High Hypoxic Risk |  | TCGA-CM-6171-01 | -0.453802363 | Low Hypoxic Risk |  | GSM358497 | 0.116525117 | High Hypoxic Risk |
| GSM972114 | 0.165172533 | High Hypoxic Risk |  | TCGA-CM-6172-01 | -0.254066802 | Low Hypoxic Risk |  | GSM358498 | -0.473155763 | Low Hypoxic Risk |
| GSM972115 | 0.131413251 | High Hypoxic Risk |  | TCGA-CM-6674-01 | -0.177839953 | Low Hypoxic Risk |  | GSM358499 | 0.129107744 | High Hypoxic Risk |
| GSM972116 | -0.06659171 | High Hypoxic Risk |  | TCGA-CM-6675-01 | -0.028217364 | High Hypoxic Risk |  | GSM358500 | 0.170888426 | High Hypoxic Risk |
| GSM972117 | -0.049051864 | High Hypoxic Risk |  | TCGA-CM-6676-01 | -0.075766915 | High Hypoxic Risk |  | GSM358501 | 0.178221854 | High Hypoxic Risk |
| GSM972118 | 0.298584474 | High Hypoxic Risk |  | TCGA-CM-6677-01 | -0.109645403 | Low Hypoxic Risk |  | GSM358502 | -0.039742955 | High Hypoxic Risk |
| GSM972119 | 0.12486409 | High Hypoxic Risk |  | TCGA-CM-6678-01 | 0.283020119 | High Hypoxic Risk |  | GSM358503 | 0.023334792 | High Hypoxic Risk |
| GSM972120 | -0.19839591 | Low Hypoxic Risk |  | TCGA-CM-6679-01 | -0.007246771 | High Hypoxic Risk |  | GSM358504 | 0.123607572 | High Hypoxic Risk |
| GSM972121 | 0.260798525 | High Hypoxic Risk |  | TCGA-CM-6680-01 | -0.300464294 | Low Hypoxic Risk |  | GSM358505 | -0.002553881 | High Hypoxic Risk |
| GSM972122 | 0.121220586 | High Hypoxic Risk |  | TCGA-D5-5537-01 | 0.029846902 | High Hypoxic Risk |  | GSM358506 | -0.170747444 | Low Hypoxic Risk |
| GSM972123 | -0.066351684 | High Hypoxic Risk |  | TCGA-D5-5538-01 | -0.09323844 | Low Hypoxic Risk |  | GSM358507 | -0.311675278 | Low Hypoxic Risk |
| GSM972124 | -0.047234875 | High Hypoxic Risk |  | TCGA-D5-5539-01 | 0.203899325 | High Hypoxic Risk |  | GSM358508 | 0.25134439 | High Hypoxic Risk |
| GSM972125 | 0.175370725 | High Hypoxic Risk |  | TCGA-D5-5540-01 | 0.417951086 | High Hypoxic Risk |  | GSM358509 | 0.078064258 | High Hypoxic Risk |
| GSM972126 | -0.043453139 | High Hypoxic Risk |  | TCGA-D5-5541-01 | -0.295345727 | Low Hypoxic Risk |  | GSM358510 | 0.327466834 | High Hypoxic Risk |
| GSM972127 | 0.07983532 | High Hypoxic Risk |  | TCGA-D5-6529-01 | -0.121168649 | Low Hypoxic Risk |  | GSM358511 | -0.260235514 | Low Hypoxic Risk |
| GSM972128 | 0.009588618 | High Hypoxic Risk |  | TCGA-D5-6530-01 | -0.150646779 | Low Hypoxic Risk |  | GSM358512 | 0.255362069 | High Hypoxic Risk |
| GSM972129 | -0.145948799 | Low Hypoxic Risk |  | TCGA-D5-6531-01 | -0.145757625 | Low Hypoxic Risk |  | GSM358513 | 0.247525085 | High Hypoxic Risk |
| GSM972130 | -0.211775124 | Low Hypoxic Risk |  | TCGA-D5-6532-01 | 0.090625123 | High Hypoxic Risk |  | GSM358514 | 0.076554685 | High Hypoxic Risk |
| GSM972131 | 0.074075014 | High Hypoxic Risk |  | TCGA-D5-6533-01 | -0.262492977 | Low Hypoxic Risk |  | GSM358515 | 0.199483049 | High Hypoxic Risk |
| GSM972132 | -0.030381007 | High Hypoxic Risk |  | TCGA-D5-6534-01 | -0.10725637 | Low Hypoxic Risk |  | GSM358516 | 0.059503071 | High Hypoxic Risk |
| GSM972133 | 0.035601502 | High Hypoxic Risk |  | TCGA-D5-6535-01 | -0.190419968 | Low Hypoxic Risk |  | GSM358517 | 0.268970474 | High Hypoxic Risk |
| GSM972134 | 0.087777262 | High Hypoxic Risk |  | TCGA-D5-6536-01 | 0.405086777 | High Hypoxic Risk |  | GSM358518 | 0.009906861 | High Hypoxic Risk |
| GSM972135 | 0.10045792 | High Hypoxic Risk |  | TCGA-D5-6537-01 | -0.081934101 | High Hypoxic Risk |  | GSM358519 | -0.174953181 | Low Hypoxic Risk |
| GSM972136 | -0.23857419 | Low Hypoxic Risk |  | TCGA-D5-6538-01 | 0.137949761 | High Hypoxic Risk |  | GSM358520 | -0.215673532 | Low Hypoxic Risk |
| GSM972137 | -0.189851174 | Low Hypoxic Risk |  | TCGA-D5-6539-01 | -0.194250105 | Low Hypoxic Risk |  | GSM358521 | -0.021679356 | High Hypoxic Risk |
| GSM972138 | -0.021246505 | High Hypoxic Risk |  | TCGA-D5-6540-01 | -0.104975861 | Low Hypoxic Risk |  | GSM358522 | -0.047184634 | High Hypoxic Risk |
| GSM972139 | 0.009058122 | High Hypoxic Risk |  | TCGA-D5-6541-01 | -0.11540455 | Low Hypoxic Risk |  | GSM358523 | 0.237170812 | High Hypoxic Risk |
| GSM972140 | 0.076976704 | High Hypoxic Risk |  | TCGA-D5-6898-01 | -0.506587155 | Low Hypoxic Risk |  | GSM358524 | 0.244058656 | High Hypoxic Risk |
| GSM972141 | 0.024844871 | High Hypoxic Risk |  | TCGA-D5-6920-01 | -0.378311387 | Low Hypoxic Risk |  | GSM358525 | 0.104655325 | High Hypoxic Risk |
| GSM972142 | -0.195623328 | Low Hypoxic Risk |  | TCGA-D5-6922-01 | -0.44349673 | Low Hypoxic Risk |  | GSM358526 | -0.011695959 | High Hypoxic Risk |
| GSM972143 | -0.113629544 | Low Hypoxic Risk |  | TCGA-D5-6923-01 | -0.337305328 | Low Hypoxic Risk |  | GSM358527 | 0.191589941 | High Hypoxic Risk |
| GSM972144 | 0.069512506 | High Hypoxic Risk |  | TCGA-D5-6924-01 | -0.200972337 | Low Hypoxic Risk |  | GSM358528 | 0.25549507 | High Hypoxic Risk |
| GSM972145 | -0.18491099 | Low Hypoxic Risk |  | TCGA-D5-6926-01 | -0.225503177 | Low Hypoxic Risk |  | GSM358529 | -0.03007835 | High Hypoxic Risk |
| GSM972146 | -0.249558001 | Low Hypoxic Risk |  | TCGA-D5-6927-01 | -0.295771015 | Low Hypoxic Risk |  | GSM358530 | 0.176531116 | High Hypoxic Risk |
| GSM972147 | -0.434301857 | Low Hypoxic Risk |  | TCGA-D5-6928-01 | -0.698827186 | Low Hypoxic Risk |  | GSM358531 | 0.197141631 | High Hypoxic Risk |
| GSM972148 | -0.251450231 | Low Hypoxic Risk |  | TCGA-D5-6929-01 | 0.214833325 | High Hypoxic Risk |  | GSM358532 | 0.065255608 | High Hypoxic Risk |
| GSM972149 | -0.032547679 | High Hypoxic Risk |  | TCGA-D5-6930-01 | -0.183252886 | Low Hypoxic Risk |  | GSM358533 | 0.156569587 | High Hypoxic Risk |
| GSM972150 | 0.012061625 | High Hypoxic Risk |  | TCGA-D5-6931-01 | -0.380096574 | Low Hypoxic Risk |  | GSM358534 | 0.053696979 | High Hypoxic Risk |
| GSM972151 | -0.225327138 | Low Hypoxic Risk |  | TCGA-D5-6932-01 | 0.248063194 | High Hypoxic Risk |  | GSM358535 | 0.270069085 | High Hypoxic Risk |
| GSM972152 | -0.273526701 | Low Hypoxic Risk |  | TCGA-D5-7000-01 | -0.317407947 | Low Hypoxic Risk |  | GSM358536 | -0.186921692 | Low Hypoxic Risk |
| GSM972153 | -0.309050142 | Low Hypoxic Risk |  | TCGA-DM-A0X9-01 | -0.044469817 | High Hypoxic Risk |  | GSM358537 | 0.13085682 | High Hypoxic Risk |
| GSM972154 | -0.194059845 | Low Hypoxic Risk |  | TCGA-DM-A0XD-01 | 0.430583774 | High Hypoxic Risk |  | GSM358538 | -0.042408102 | High Hypoxic Risk |
| GSM972155 | -0.077323119 | High Hypoxic Risk |  | TCGA-DM-A0XF-01 | 0.022260188 | High Hypoxic Risk |  | GSM358539 | -0.055060224 | High Hypoxic Risk |
| GSM972156 | -0.053425357 | High Hypoxic Risk |  | TCGA-DM-A1D0-01 | 0.877435555 | High Hypoxic Risk |  | GSM358540 | 0.050686433 | High Hypoxic Risk |
| GSM972157 | 0.094818757 | High Hypoxic Risk |  | TCGA-DM-A1D4-01 | 0.006760441 | High Hypoxic Risk |  | GSM358541 | 0.63154418 | High Hypoxic Risk |
| GSM972158 | -0.253710599 | Low Hypoxic Risk |  | TCGA-DM-A1D6-01 | 0.165972536 | High Hypoxic Risk |  | GSM358542 | -0.141171562 | Low Hypoxic Risk |
| GSM972159 | -0.190174695 | Low Hypoxic Risk |  | TCGA-DM-A1D7-01 | 0.081051725 | High Hypoxic Risk |  | GSM358543 | 0.137156203 | High Hypoxic Risk |
| GSM972160 | 0.117818229 | High Hypoxic Risk |  | TCGA-DM-A1D8-01 | 0.059966203 | High Hypoxic Risk |  | GSM358544 | 0.153341216 | High Hypoxic Risk |
| GSM972161 | 0.145168745 | High Hypoxic Risk |  | TCGA-DM-A1D9-01 | 0.11024391 | High Hypoxic Risk |  | GSM358545 | -0.247894106 | Low Hypoxic Risk |
| GSM972162 | -0.049462223 | High Hypoxic Risk |  | TCGA-DM-A1DA-01 | 0.161107642 | High Hypoxic Risk |  | GSM358546 | -0.225243772 | Low Hypoxic Risk |
| GSM972163 | -0.096886363 | Low Hypoxic Risk |  | TCGA-DM-A1DB-01 | -0.102008261 | Low Hypoxic Risk |  | GSM358547 | -0.164574661 | Low Hypoxic Risk |
| GSM972164 | -0.207139142 | Low Hypoxic Risk |  | TCGA-DM-A1HA-01 | 0.242374868 | High Hypoxic Risk |  | GSM358548 | 0.127459816 | High Hypoxic Risk |
| GSM972165 | 0.039203587 | High Hypoxic Risk |  | TCGA-DM-A1HB-01 | 0.230457605 | High Hypoxic Risk |  | GSM358549 | -0.224877231 | Low Hypoxic Risk |
| GSM972166 | 0.002988716 | High Hypoxic Risk |  | TCGA-DM-A280-01 | 0.330025609 | High Hypoxic Risk |  | GSM358550 | -0.10254929 | Low Hypoxic Risk |
| GSM972167 | -0.143492355 | Low Hypoxic Risk |  | TCGA-DM-A282-01 | 0.184607431 | High Hypoxic Risk |  | GSM358551 | -0.097215515 | Low Hypoxic Risk |
| GSM972168 | 0.167394699 | High Hypoxic Risk |  | TCGA-DM-A285-01 | 0.079998347 | High Hypoxic Risk |  | GSM358552 | -0.19023564 | Low Hypoxic Risk |
| GSM972169 | -0.092620211 | Low Hypoxic Risk |  | TCGA-DM-A288-01 | 0.3145831 | High Hypoxic Risk |  | GSM358553 | -0.001000017 | High Hypoxic Risk |
| GSM972170 | -0.079114282 | High Hypoxic Risk |  | TCGA-DM-A28A-01 | -0.034642399 | High Hypoxic Risk |  | GSM358554 | -0.302223991 | Low Hypoxic Risk |
| GSM972171 | 0.118659382 | High Hypoxic Risk |  | TCGA-DM-A28C-01 | 0.079191347 | High Hypoxic Risk |  | GSM358555 | -0.023671704 | High Hypoxic Risk |
| GSM972172 | 0.619535305 | High Hypoxic Risk |  | TCGA-DM-A28E-01 | -0.034585285 | High Hypoxic Risk |  | GSM358556 | -0.209524603 | Low Hypoxic Risk |
| GSM972173 | 0.353552879 | High Hypoxic Risk |  | TCGA-DM-A28F-01 | -0.14353255 | Low Hypoxic Risk |  | GSM358557 | -0.199742237 | Low Hypoxic Risk |
| GSM972174 | 0.338877617 | High Hypoxic Risk |  | TCGA-DM-A28G-01 | 0.095161491 | High Hypoxic Risk |  | GSM358558 | 0.171618784 | High Hypoxic Risk |
| GSM972175 | 0.433859671 | High Hypoxic Risk |  | TCGA-DM-A28H-01 | 0.2913472 | High Hypoxic Risk |  | GSM358559 | -0.214918348 | Low Hypoxic Risk |
| GSM972176 | 0.363855322 | High Hypoxic Risk |  | TCGA-DM-A28K-01 | 0.370837967 | High Hypoxic Risk |  | GSM358560 | 0.027995096 | High Hypoxic Risk |
| GSM972177 | 0.63297748 | High Hypoxic Risk |  | TCGA-DM-A28M-01 | 0.084343326 | High Hypoxic Risk |  | GSM358561 | -0.253294591 | Low Hypoxic Risk |
| GSM972178 | -0.076358708 | High Hypoxic Risk |  | TCGA-F4-6459-01 | 0.012986914 | High Hypoxic Risk |  | GSM358562 | -0.119150138 | Low Hypoxic Risk |
| GSM972179 | 0.384606748 | High Hypoxic Risk |  | TCGA-F4-6460-01 | 0.146374255 | High Hypoxic Risk |  | GSM358563 | -0.11691602 | Low Hypoxic Risk |
| GSM972180 | -0.09528038 | Low Hypoxic Risk |  | TCGA-F4-6461-01 | 0.084199456 | High Hypoxic Risk |  | GSM358564 | -0.104542407 | Low Hypoxic Risk |
| GSM972181 | 0.672153446 | High Hypoxic Risk |  | TCGA-F4-6463-01 | -0.154838828 | Low Hypoxic Risk |  | GSM358565 | -0.012057467 | High Hypoxic Risk |
| GSM972182 | 0.358436814 | High Hypoxic Risk |  | TCGA-F4-6569-01 | -0.188608061 | Low Hypoxic Risk |  | GSM358566 | -0.074621156 | High Hypoxic Risk |
| GSM972183 | 0.459619693 | High Hypoxic Risk |  | TCGA-F4-6570-01 | -0.180728745 | Low Hypoxic Risk |  | GSM358567 | 0.108954834 | High Hypoxic Risk |
| GSM972184 | 0.433875888 | High Hypoxic Risk |  | TCGA-F4-6703-01 | -0.045647146 | High Hypoxic Risk |  | GSM358568 | -0.042848168 | High Hypoxic Risk |
| GSM972185 | 0.463540589 | High Hypoxic Risk |  | TCGA-F4-6704-01 | -0.113789692 | Low Hypoxic Risk |  | GSM358569 | -0.12854018 | Low Hypoxic Risk |
| GSM972186 | 0.362677196 | High Hypoxic Risk |  | TCGA-F4-6805-01 | -0.178658157 | Low Hypoxic Risk |  | GSM358570 | 0.050162921 | High Hypoxic Risk |
| GSM972187 | -0.144675883 | Low Hypoxic Risk |  | TCGA-F4-6806-01 | -0.047366645 | High Hypoxic Risk |  | GSM358571 | 0.531392691 | High Hypoxic Risk |
| GSM972188 | 0.133920054 | High Hypoxic Risk |  | TCGA-F4-6807-01 | -0.140681636 | Low Hypoxic Risk |  | GSM358572 | 0.013775681 | High Hypoxic Risk |
| GSM972189 | -0.41109131 | Low Hypoxic Risk |  | TCGA-F4-6808-01 | 0.232048918 | High Hypoxic Risk |  | GSM358573 | -0.19545132 | Low Hypoxic Risk |
| GSM972190 | 0.18486576 | High Hypoxic Risk |  | TCGA-F4-6809-01 | 0.228318067 | High Hypoxic Risk |  | GSM358574 | -0.005968272 | High Hypoxic Risk |
| GSM972191 | -0.119613737 | Low Hypoxic Risk |  | TCGA-F4-6854-01 | -0.276578303 | Low Hypoxic Risk |  | GSM358575 | 0.206183952 | High Hypoxic Risk |
| GSM972192 | 0.131728575 | High Hypoxic Risk |  | TCGA-F4-6855-01 | -0.000516064 | High Hypoxic Risk |  | GSM358576 | -0.113227171 | Low Hypoxic Risk |
| GSM972193 | 0.324486183 | High Hypoxic Risk |  | TCGA-F4-6856-01 | -0.284732786 | Low Hypoxic Risk |  | GSM358577 | 0.10972844 | High Hypoxic Risk |
| GSM972194 | 0.46652724 | High Hypoxic Risk |  | TCGA-G4-6293-01 | -0.263462396 | Low Hypoxic Risk |  | GSM358578 | -0.148637862 | Low Hypoxic Risk |
| GSM972195 | 0.135292374 | High Hypoxic Risk |  | TCGA-G4-6294-01 | 0.07576654 | High Hypoxic Risk |  | GSM358579 | -0.026127642 | High Hypoxic Risk |
| GSM972196 | -0.3533738 | Low Hypoxic Risk |  | TCGA-G4-6295-01 | -0.482122007 | Low Hypoxic Risk |  | GSM358580 | 0.178243617 | High Hypoxic Risk |
| GSM972197 | -0.046303928 | High Hypoxic Risk |  | TCGA-G4-6297-01 | -0.10031195 | Low Hypoxic Risk |  | GSM358581 | 0.090418408 | High Hypoxic Risk |
| GSM972198 | -0.019814608 | High Hypoxic Risk |  | TCGA-G4-6298-01 | 0.411209611 | High Hypoxic Risk |  | GSM358582 | -0.272532538 | Low Hypoxic Risk |
| GSM972199 | -0.029427847 | High Hypoxic Risk |  | TCGA-G4-6299-01 | -0.08308522 | Low Hypoxic Risk |  | GSM358583 | 0.221919554 | High Hypoxic Risk |
| GSM972200 | -0.121458112 | Low Hypoxic Risk |  | TCGA-G4-6302-01 | 0.083302898 | High Hypoxic Risk |  | GSM358584 | 0.139491124 | High Hypoxic Risk |
| GSM972201 | 0.147497943 | High Hypoxic Risk |  | TCGA-G4-6303-01 | -0.218734634 | Low Hypoxic Risk |  | GSM358585 | -0.024765635 | High Hypoxic Risk |
| GSM972202 | -0.308671463 | Low Hypoxic Risk |  | TCGA-G4-6304-01 | 0.011844392 | High Hypoxic Risk |  | GSM358586 | 0.094644058 | High Hypoxic Risk |
| GSM972203 | 0.083876091 | High Hypoxic Risk |  | TCGA-G4-6306-01 | 0.054483947 | High Hypoxic Risk |  | GSM358587 | 0.504785074 | High Hypoxic Risk |
| GSM972204 | 0.021274142 | High Hypoxic Risk |  | TCGA-G4-6307-01 | 0.162024793 | High Hypoxic Risk |  | GSM358588 | 0.167652349 | High Hypoxic Risk |
| GSM972205 | -0.344965944 | Low Hypoxic Risk |  | TCGA-G4-6309-01 | -0.092302076 | Low Hypoxic Risk |  | GSM358589 | -0.296641125 | Low Hypoxic Risk |
| GSM972206 | 0.084938729 | High Hypoxic Risk |  | TCGA-G4-6310-01 | 0.070955124 | High Hypoxic Risk |  | GSM358590 | 0.027493114 | High Hypoxic Risk |
| GSM972207 | -0.284983051 | Low Hypoxic Risk |  | TCGA-G4-6311-01 | -0.06976163 | High Hypoxic Risk |  | GSM358591 | 0.186162805 | High Hypoxic Risk |
| GSM972208 | -0.002212754 | High Hypoxic Risk |  | TCGA-G4-6314-01 | -0.064332745 | High Hypoxic Risk |  | GSM358592 | -0.137864892 | Low Hypoxic Risk |
| GSM972209 | 0.042868403 | High Hypoxic Risk |  | TCGA-G4-6315-01 | 0.012548 | High Hypoxic Risk |  | GSM358593 | 0.458743805 | High Hypoxic Risk |
| GSM972210 | 0.035683671 | High Hypoxic Risk |  | TCGA-G4-6317-01 | 0.197019399 | High Hypoxic Risk |  | GSM358594 | -0.01270435 | High Hypoxic Risk |
| GSM972211 | 0.0989874 | High Hypoxic Risk |  | TCGA-G4-6317-02 | 0.270275234 | High Hypoxic Risk |  | GSM358595 | 0.01063053 | High Hypoxic Risk |
| GSM972212 | -0.239685141 | Low Hypoxic Risk |  | TCGA-G4-6320-01 | 0.276780024 | High Hypoxic Risk |  | GSM358596 | -0.041010425 | High Hypoxic Risk |
| GSM972213 | -0.101066979 | Low Hypoxic Risk |  | TCGA-G4-6321-01 | 0.063105445 | High Hypoxic Risk |  | GSM358597 | 0.264604726 | High Hypoxic Risk |
| GSM972214 | -0.069128828 | High Hypoxic Risk |  | TCGA-G4-6322-01 | -0.0625239 | High Hypoxic Risk |  | GSM358598 | -0.185999826 | Low Hypoxic Risk |
| GSM972215 | -0.099526927 | Low Hypoxic Risk |  | TCGA-G4-6323-01 | -0.035417883 | High Hypoxic Risk |  | GSM358599 | -0.103960277 | Low Hypoxic Risk |
| GSM972216 | -0.132119492 | Low Hypoxic Risk |  | TCGA-G4-6586-01 | -0.071112766 | High Hypoxic Risk |  | GSM358600 | 0.181975195 | High Hypoxic Risk |
| GSM972217 | 0.159961274 | High Hypoxic Risk |  | TCGA-G4-6588-01 | -0.269931801 | Low Hypoxic Risk |  | GSM358601 | 0.154329105 | High Hypoxic Risk |
| GSM972218 | 0.331014928 | High Hypoxic Risk |  | TCGA-G4-6625-01 | -0.216457488 | Low Hypoxic Risk |  | GSM358602 | -0.014056928 | High Hypoxic Risk |
| GSM972219 | -0.254965302 | Low Hypoxic Risk |  | TCGA-G4-6626-01 | -0.197196763 | Low Hypoxic Risk |  | GSM358603 | 0.421829687 | High Hypoxic Risk |
| GSM972220 | -0.492138004 | Low Hypoxic Risk |  | TCGA-G4-6627-01 | -0.220831669 | Low Hypoxic Risk |  | GSM358604 | 0.135991869 | High Hypoxic Risk |
| GSM972221 | -0.532505479 | Low Hypoxic Risk |  | TCGA-G4-6628-01 | -0.498844628 | Low Hypoxic Risk |  | GSM358605 | 0.112141019 | High Hypoxic Risk |
| GSM972222 | -0.347591318 | Low Hypoxic Risk |  | TCGA-NH-A50T-01 | 0.054616616 | High Hypoxic Risk |  | GSM358606 | 0.106540232 | High Hypoxic Risk |
| GSM972223 | -0.0404902 | High Hypoxic Risk |  | TCGA-NH-A50U-01 | -0.121281903 | Low Hypoxic Risk |  | GSM358607 | -0.040650758 | High Hypoxic Risk |
| GSM972224 | -0.110462254 | Low Hypoxic Risk |  | TCGA-NH-A50V-01 | 0.054296834 | High Hypoxic Risk |  | GSM358608 | -0.123003414 | Low Hypoxic Risk |
| GSM972225 | -0.138351235 | Low Hypoxic Risk |  | TCGA-NH-A5IV-01 | -0.291819216 | Low Hypoxic Risk |  | GSM358609 | 0.15441933 | High Hypoxic Risk |
| GSM972226 | -0.124771054 | Low Hypoxic Risk |  | TCGA-NH-A6GA-01 | -0.168357947 | Low Hypoxic Risk |  | GSM358610 | -0.130238422 | Low Hypoxic Risk |
| GSM972227 | 0.04180982 | High Hypoxic Risk |  | TCGA-NH-A6GB-01 | -0.058254326 | High Hypoxic Risk |  | GSM358611 | 0.116558326 | High Hypoxic Risk |
| GSM972228 | -0.10818502 | Low Hypoxic Risk |  | TCGA-NH-A6GC-01 | 0.155687711 | High Hypoxic Risk |  | GSM358612 | 0.01046902 | High Hypoxic Risk |
| GSM972229 | -0.667881982 | Low Hypoxic Risk |  | TCGA-NH-A8F7-06 | 0.066424585 | High Hypoxic Risk |  | GSM358613 | -0.212074717 | Low Hypoxic Risk |
| GSM972230 | 0.05945509 | High Hypoxic Risk |  | TCGA-NH-A8F7-01 | -0.108483491 | Low Hypoxic Risk |  | GSM358614 | 0.007583779 | High Hypoxic Risk |
| GSM972231 | 0.256390362 | High Hypoxic Risk |  | TCGA-NH-A8F8-01 | 0.082075738 | High Hypoxic Risk |  | GSM358615 | -0.197449896 | Low Hypoxic Risk |
| GSM972232 | 0.01293305 | High Hypoxic Risk |  | TCGA-QG-A5YV-01 | -0.116762672 | Low Hypoxic Risk |  | GSM358616 | -0.230615857 | Low Hypoxic Risk |
| GSM972233 | 0.201579068 | High Hypoxic Risk |  | TCGA-QG-A5YW-01 | 0.028648773 | High Hypoxic Risk |  | GSM358617 | 0.081634052 | High Hypoxic Risk |
| GSM972234 | 0.095900087 | High Hypoxic Risk |  | TCGA-QG-A5YX-01 | -0.202791608 | Low Hypoxic Risk |  | GSM358618 | -0.045767469 | High Hypoxic Risk |
| GSM972235 | -0.175138898 | Low Hypoxic Risk |  | TCGA-QG-A5Z1-01 | -0.043953477 | High Hypoxic Risk |  | GSM358619 | -0.024806593 | High Hypoxic Risk |
| GSM972236 | 0.193436264 | High Hypoxic Risk |  | TCGA-QG-A5Z2-01 | -0.501965994 | Low Hypoxic Risk |  | GSM358620 | 0.048877606 | High Hypoxic Risk |
| GSM972237 | 0.052823268 | High Hypoxic Risk |  | TCGA-QL-A97D-01 | -0.261278781 | Low Hypoxic Risk |  | GSM358621 | 0.508096276 | High Hypoxic Risk |
| GSM972238 | -0.109873649 | Low Hypoxic Risk |  | TCGA-RU-A8FL-01 | 0.135621517 | High Hypoxic Risk |  | GSM358622 | -0.178419309 | Low Hypoxic Risk |
| GSM972239 | -0.191804552 | Low Hypoxic Risk |  | TCGA-SS-A7HO-01 | 0.08056527 | High Hypoxic Risk |  | GSM358623 | 0.103838206 | High Hypoxic Risk |
| GSM972240 | -0.048302681 | High Hypoxic Risk |  | TCGA-T9-A92H-01 | 0.00110822 | High Hypoxic Risk |  | GSM358624 | -0.111207197 | Low Hypoxic Risk |
| GSM972241 | -0.257216442 | Low Hypoxic Risk |  | TCGA-WS-AB45-01 | -0.071437715 | High Hypoxic Risk |  | GSM358625 | -0.07383696 | High Hypoxic Risk |
| GSM972242 | -0.196390088 | Low Hypoxic Risk |  | TCGA-AF-2687-01 | -0.160599371 | Low Hypoxic Risk |  | GSM358626 | 0.039780812 | High Hypoxic Risk |
| GSM972243 | 0.208211374 | High Hypoxic Risk |  | TCGA-AF-2690-01 | 0.027472447 | High Hypoxic Risk |  | GSM358627 | 0.059683946 | High Hypoxic Risk |
| GSM972244 | 0.517073853 | High Hypoxic Risk |  | TCGA-AF-2693-01 | -0.141370932 | Low Hypoxic Risk |  | GSM358628 | -0.238019815 | Low Hypoxic Risk |
| GSM972245 | 0.259556306 | High Hypoxic Risk |  | TCGA-AF-3911-01 | 0.024198827 | High Hypoxic Risk |  | GSM358629 | 0.134000432 | High Hypoxic Risk |
| GSM972246 | 0.062558598 | High Hypoxic Risk |  | TCGA-AF-4110-01 | -0.004375358 | High Hypoxic Risk |  | GSM358630 | -0.187023976 | Low Hypoxic Risk |
| GSM972247 | 0.360709993 | High Hypoxic Risk |  | TCGA-AF-5654-01 | -0.068090043 | High Hypoxic Risk |  | GSM437093 | -0.095561307 | Low Hypoxic Risk |
| GSM972248 | 0.379145263 | High Hypoxic Risk |  | TCGA-AF-6136-01 | 0.061472434 | High Hypoxic Risk |  | GSM437094 | 0.18391135 | High Hypoxic Risk |
| GSM972249 | 0.09662302 | High Hypoxic Risk |  | TCGA-AF-6655-01 | -0.137441 | Low Hypoxic Risk |  | GSM437095 | 0.263041666 | High Hypoxic Risk |
| GSM972250 | -0.01050149 | High Hypoxic Risk |  | TCGA-AF-6672-01 | 0.030309512 | High Hypoxic Risk |  | GSM437096 | 0.030050429 | High Hypoxic Risk |
| GSM972251 | -0.106039015 | Low Hypoxic Risk |  | TCGA-AF-A56K-01 | -0.108551501 | Low Hypoxic Risk |  | GSM437097 | -0.453579187 | Low Hypoxic Risk |
| GSM972252 | -0.004725042 | High Hypoxic Risk |  | TCGA-AF-A56L-01 | 0.021420613 | High Hypoxic Risk |  | GSM437098 | -0.169719572 | Low Hypoxic Risk |
| GSM972253 | 0.101279925 | High Hypoxic Risk |  | TCGA-AF-A56N-01 | -0.100870763 | Low Hypoxic Risk |  | GSM437099 | -0.073832994 | High Hypoxic Risk |
| GSM972254 | 0.389285305 | High Hypoxic Risk |  | TCGA-AG-3591-01 | 0.239858336 | High Hypoxic Risk |  | GSM437100 | 0.107669563 | High Hypoxic Risk |
| GSM972255 | 0.071485263 | High Hypoxic Risk |  | TCGA-AG-3592-01 | -0.020216971 | High Hypoxic Risk |  | GSM437101 | -0.028967373 | High Hypoxic Risk |
| GSM972256 | 0.357724292 | High Hypoxic Risk |  | TCGA-AG-3725-01 | -0.058467828 | High Hypoxic Risk |  | GSM437102 | 0.226616107 | High Hypoxic Risk |
| GSM972257 | 0.453334292 | High Hypoxic Risk |  | TCGA-AG-3731-01 | -0.430054637 | Low Hypoxic Risk |  | GSM437103 | -0.265739697 | Low Hypoxic Risk |
| GSM972258 | 0.32302928 | High Hypoxic Risk |  | TCGA-AG-3732-01 | -0.411239475 | Low Hypoxic Risk |  | GSM437104 | -0.527527162 | Low Hypoxic Risk |
| GSM972259 | 0.303551117 | High Hypoxic Risk |  | TCGA-AG-3742-01 | -0.197801688 | Low Hypoxic Risk |  | GSM437105 | -0.04684317 | High Hypoxic Risk |
| GSM972260 | 0.303162996 | High Hypoxic Risk |  | TCGA-AG-3902-01 | -0.352756788 | Low Hypoxic Risk |  | GSM437106 | -0.36646731 | Low Hypoxic Risk |
| GSM972261 | -0.375915236 | Low Hypoxic Risk |  | TCGA-AG-4021-01 | 0.107519326 | High Hypoxic Risk |  | GSM437107 | -0.138659447 | Low Hypoxic Risk |
| GSM972262 | 0.064162402 | High Hypoxic Risk |  | TCGA-AG-4022-01 | 0.03281566 | High Hypoxic Risk |  | GSM437108 | -0.340918494 | Low Hypoxic Risk |
| GSM972263 | -0.434207617 | Low Hypoxic Risk |  | TCGA-AH-6544-01 | 0.18648248 | High Hypoxic Risk |  | GSM437109 | -0.000640381 | High Hypoxic Risk |
| GSM972264 | -0.515629586 | Low Hypoxic Risk |  | TCGA-AH-6547-01 | -0.240499512 | Low Hypoxic Risk |  | GSM437110 | 0.078213017 | High Hypoxic Risk |
| GSM972265 | 0.084800563 | High Hypoxic Risk |  | TCGA-AH-6549-01 | -0.194294554 | Low Hypoxic Risk |  | GSM437111 | 0.249865243 | High Hypoxic Risk |
| GSM972266 | -0.330557956 | Low Hypoxic Risk |  | TCGA-AH-6643-01 | 0.113216408 | High Hypoxic Risk |  | GSM437112 | -0.189329281 | Low Hypoxic Risk |
| GSM972267 | 0.100004279 | High Hypoxic Risk |  | TCGA-AH-6644-01 | -0.042487805 | High Hypoxic Risk |  | GSM437113 | 0.164259637 | High Hypoxic Risk |
| GSM972268 | 0.05302122 | High Hypoxic Risk |  | TCGA-AH-6897-01 | -0.385322688 | Low Hypoxic Risk |  | GSM437114 | -0.198060612 | Low Hypoxic Risk |
| GSM972269 | 0.503904067 | High Hypoxic Risk |  | TCGA-AH-6903-01 | -0.232172109 | Low Hypoxic Risk |  | GSM437115 | 0.031520636 | High Hypoxic Risk |
| GSM972270 | -0.320826957 | Low Hypoxic Risk |  | TCGA-BM-6198-01 | 0.181095699 | High Hypoxic Risk |  | GSM437116 | 0.053666168 | High Hypoxic Risk |
| GSM972271 | 0.211588812 | High Hypoxic Risk |  | TCGA-CI-6619-01 | -0.153532007 | Low Hypoxic Risk |  | GSM437117 | 0.078514638 | High Hypoxic Risk |
| GSM972272 | -0.03087488 | High Hypoxic Risk |  | TCGA-CI-6620-01 | -0.035195303 | High Hypoxic Risk |  | GSM437118 | 0.10024819 | High Hypoxic Risk |
| GSM972273 | 0.082205684 | High Hypoxic Risk |  | TCGA-CI-6621-01 | -0.346164618 | Low Hypoxic Risk |  | GSM437119 | 0.289239065 | High Hypoxic Risk |
| GSM972274 | -0.436644109 | Low Hypoxic Risk |  | TCGA-CI-6622-01 | -0.127786644 | Low Hypoxic Risk |  | GSM437120 | -0.257332957 | Low Hypoxic Risk |
| GSM972275 | -0.031991944 | High Hypoxic Risk |  | TCGA-CI-6623-01 | -0.149642651 | Low Hypoxic Risk |  | GSM437121 | -0.098910839 | Low Hypoxic Risk |
| GSM972276 | -0.468066161 | Low Hypoxic Risk |  | TCGA-CI-6624-01 | -0.220955702 | Low Hypoxic Risk |  | GSM437122 | -0.130358185 | Low Hypoxic Risk |
| GSM972277 | -0.248033683 | Low Hypoxic Risk |  | TCGA-CL-4957-01 | 0.035728077 | High Hypoxic Risk |  | GSM437123 | -0.074810277 | High Hypoxic Risk |
| GSM972278 | -0.380321476 | Low Hypoxic Risk |  | TCGA-CL-5917-01 | -0.153594317 | Low Hypoxic Risk |  | GSM437124 | 0.046805706 | High Hypoxic Risk |
| GSM972279 | -0.382017228 | Low Hypoxic Risk |  | TCGA-CL-5918-01 | 0.113618833 | High Hypoxic Risk |  | GSM437125 | -0.0515617 | High Hypoxic Risk |
| GSM972280 | -0.199939231 | Low Hypoxic Risk |  | TCGA-DC-4745-01 | 0.173583238 | High Hypoxic Risk |  | GSM437126 | -0.341837322 | Low Hypoxic Risk |
| GSM972281 | -0.023821972 | High Hypoxic Risk |  | TCGA-DC-4749-01 | 0.010283751 | High Hypoxic Risk |  | GSM437127 | 0.036610252 | High Hypoxic Risk |
| GSM972282 | -0.282645113 | Low Hypoxic Risk |  | TCGA-DC-5337-01 | 0.062759803 | High Hypoxic Risk |  | GSM437128 | -0.157003361 | Low Hypoxic Risk |
| GSM972283 | -0.199383714 | Low Hypoxic Risk |  | TCGA-DC-5869-01 | -0.194512792 | Low Hypoxic Risk |  | GSM437129 | -0.482629405 | Low Hypoxic Risk |
| GSM972284 | -0.004228887 | High Hypoxic Risk |  | TCGA-DC-6154-01 | 0.127397617 | High Hypoxic Risk |  | GSM437130 | -0.030487955 | High Hypoxic Risk |
| GSM972285 | -0.512962479 | Low Hypoxic Risk |  | TCGA-DC-6155-01 | 1.386247299 | High Hypoxic Risk |  | GSM437131 | 0.158073002 | High Hypoxic Risk |
| GSM972286 | -0.43355193 | Low Hypoxic Risk |  | TCGA-DC-6156-01 | -0.317182376 | Low Hypoxic Risk |  | GSM437132 | -0.114465525 | Low Hypoxic Risk |
| GSM972287 | 0.149406572 | High Hypoxic Risk |  | TCGA-DC-6157-01 | -0.452840289 | Low Hypoxic Risk |  | GSM437133 | -0.045843184 | High Hypoxic Risk |
| GSM972288 | 0.489454748 | High Hypoxic Risk |  | TCGA-DC-6158-01 | -0.372374559 | Low Hypoxic Risk |  | GSM437134 | 0.560034334 | High Hypoxic Risk |
| GSM972289 | -0.016237818 | High Hypoxic Risk |  | TCGA-DC-6160-01 | -0.38694939 | Low Hypoxic Risk |  | GSM437135 | 0.28686311 | High Hypoxic Risk |
| GSM972290 | -0.443836642 | Low Hypoxic Risk |  | TCGA-DC-6681-01 | -0.070754849 | High Hypoxic Risk |  | GSM437136 | 0.187308201 | High Hypoxic Risk |
| GSM972291 | -0.518722517 | Low Hypoxic Risk |  | TCGA-DC-6682-01 | -0.304398609 | Low Hypoxic Risk |  | GSM437137 | -0.282384225 | Low Hypoxic Risk |
| GSM972292 | -0.250265047 | Low Hypoxic Risk |  | TCGA-DC-6683-01 | -0.015784573 | High Hypoxic Risk |  | GSM437138 | 0.158981923 | High Hypoxic Risk |
| GSM972293 | 0.013991106 | High Hypoxic Risk |  | TCGA-DT-5265-01 | 0.265787236 | High Hypoxic Risk |  | GSM437139 | 0.10100527 | High Hypoxic Risk |
| GSM972294 | -0.41508723 | Low Hypoxic Risk |  | TCGA-DY-A0XA-01 | -0.180793644 | Low Hypoxic Risk |  | GSM437140 | 0.227800004 | High Hypoxic Risk |
| GSM972295 | 0.122687474 | High Hypoxic Risk |  | TCGA-DY-A1DC-01 | 0.15851668 | High Hypoxic Risk |  | GSM437141 | -0.250252749 | Low Hypoxic Risk |
| GSM972296 | -0.62768989 | Low Hypoxic Risk |  | TCGA-DY-A1DD-01 | 0.482002257 | High Hypoxic Risk |  | GSM437142 | 0.407786596 | High Hypoxic Risk |
| GSM972297 | 0.025995013 | High Hypoxic Risk |  | TCGA-DY-A1DE-01 | -0.001157783 | High Hypoxic Risk |  | GSM437143 | -0.352869808 | Low Hypoxic Risk |
| GSM972298 | -0.367284924 | Low Hypoxic Risk |  | TCGA-DY-A1DF-01 | 0.78376627 | High Hypoxic Risk |  | GSM437144 | 0.078439268 | High Hypoxic Risk |
| GSM972299 | -0.016251412 | High Hypoxic Risk |  | TCGA-DY-A1DG-01 | 0.106936328 | High Hypoxic Risk |  | GSM437145 | -0.036408831 | High Hypoxic Risk |
| GSM972300 | -0.080612431 | High Hypoxic Risk |  | TCGA-DY-A1H8-01 | 0.41453599 | High Hypoxic Risk |  | GSM437146 | 0.03151772 | High Hypoxic Risk |
| GSM972301 | 0.138009199 | High Hypoxic Risk |  | TCGA-EF-5830-01 | -0.046429342 | High Hypoxic Risk |  | GSM437147 | -0.298171461 | Low Hypoxic Risk |
| GSM972302 | -0.159007021 | Low Hypoxic Risk |  | TCGA-EF-5831-01 | -0.150307615 | Low Hypoxic Risk |  | GSM437148 | -0.28045374 | Low Hypoxic Risk |
| GSM972303 | -0.033036564 | High Hypoxic Risk |  | TCGA-EI-6506-01 | -0.199457487 | Low Hypoxic Risk |  | GSM437149 | -0.089959899 | Low Hypoxic Risk |
| GSM972304 | -0.202502248 | Low Hypoxic Risk |  | TCGA-EI-6507-01 | -0.155276928 | Low Hypoxic Risk |  | GSM437150 | 0.752055673 | High Hypoxic Risk |
| GSM972305 | 0.135241265 | High Hypoxic Risk |  | TCGA-EI-6508-01 | -0.30858613 | Low Hypoxic Risk |  | GSM437151 | -0.003651551 | High Hypoxic Risk |
| GSM972306 | 0.030284362 | High Hypoxic Risk |  | TCGA-EI-6509-01 | 0.059719628 | High Hypoxic Risk |  | GSM437152 | 0.084150555 | High Hypoxic Risk |
| GSM972307 | 0.060651563 | High Hypoxic Risk |  | TCGA-EI-6510-01 | -0.25405293 | Low Hypoxic Risk |  | GSM437153 | 0.28789995 | High Hypoxic Risk |
| GSM972308 | -0.350107901 | Low Hypoxic Risk |  | TCGA-EI-6511-01 | -0.250054201 | Low Hypoxic Risk |  | GSM437154 | -0.000923854 | High Hypoxic Risk |
| GSM972309 | 0.04713997 | High Hypoxic Risk |  | TCGA-EI-6512-01 | -0.180149637 | Low Hypoxic Risk |  | GSM437155 | 0.127753671 | High Hypoxic Risk |
| GSM972310 | 0.203668538 | High Hypoxic Risk |  | TCGA-EI-6513-01 | -0.009308663 | High Hypoxic Risk |  | GSM437156 | 0.056062945 | High Hypoxic Risk |
| GSM972311 | 0.79054692 | High Hypoxic Risk |  | TCGA-EI-6514-01 | 0.002796391 | High Hypoxic Risk |  | GSM437157 | 0.106917869 | High Hypoxic Risk |
| GSM972312 | -0.008421237 | High Hypoxic Risk |  | TCGA-EI-6881-01 | -0.080017935 | High Hypoxic Risk |  | GSM437158 | 0.20438825 | High Hypoxic Risk |
| GSM972313 | -0.06736179 | High Hypoxic Risk |  | TCGA-EI-6882-01 | -0.480780718 | Low Hypoxic Risk |  | GSM437159 | 0.016169956 | High Hypoxic Risk |
| GSM972314 | -0.176150559 | Low Hypoxic Risk |  | TCGA-EI-6883-01 | -0.144996737 | Low Hypoxic Risk |  | GSM437160 | -0.328775306 | Low Hypoxic Risk |
| GSM972315 | -0.147119772 | Low Hypoxic Risk |  | TCGA-EI-6884-01 | -0.14213545 | Low Hypoxic Risk |  | GSM437161 | -0.165591459 | Low Hypoxic Risk |
| GSM972316 | -0.113048546 | Low Hypoxic Risk |  | TCGA-EI-6885-01 | -0.449415677 | Low Hypoxic Risk |  | GSM437162 | -0.168146795 | Low Hypoxic Risk |
| GSM972317 | 0.0254032 | High Hypoxic Risk |  | TCGA-EI-6917-01 | -0.33723751 | Low Hypoxic Risk |  | GSM437163 | 0.322102032 | High Hypoxic Risk |
| GSM972318 | 0.186487825 | High Hypoxic Risk |  | TCGA-EI-7002-01 | -0.056252775 | High Hypoxic Risk |  | GSM437164 | 0.114794333 | High Hypoxic Risk |
| GSM972319 | 0.211179906 | High Hypoxic Risk |  | TCGA-EI-7004-01 | -0.021492516 | High Hypoxic Risk |  | GSM437165 | -0.090981189 | Low Hypoxic Risk |
| GSM972320 | 0.27601046 | High Hypoxic Risk |  | TCGA-F5-6464-01 | -0.074716219 | High Hypoxic Risk |  | GSM437166 | -0.179992303 | Low Hypoxic Risk |
| GSM972321 | 0.226307144 | High Hypoxic Risk |  | TCGA-F5-6465-01 | -0.239911588 | Low Hypoxic Risk |  | GSM437167 | -0.270885549 | Low Hypoxic Risk |
| GSM972322 | -0.220041903 | Low Hypoxic Risk |  | TCGA-F5-6571-01 | -0.009085278 | High Hypoxic Risk |  | GSM437168 | -0.127893601 | Low Hypoxic Risk |
| GSM972323 | 0.08781025 | High Hypoxic Risk |  | TCGA-F5-6702-01 | 0.057626944 | High Hypoxic Risk |  | GSM437169 | 0.270663162 | High Hypoxic Risk |
| GSM972324 | -0.115073137 | Low Hypoxic Risk |  | TCGA-F5-6810-01 | 0.005581214 | High Hypoxic Risk |  | GSM437170 | 0.231432601 | High Hypoxic Risk |
| GSM972325 | -0.125114875 | Low Hypoxic Risk |  | TCGA-F5-6811-01 | -0.157191724 | Low Hypoxic Risk |  | GSM437171 | -0.359704202 | Low Hypoxic Risk |
| GSM972326 | 0.101968009 | High Hypoxic Risk |  | TCGA-F5-6812-01 | 0.048491075 | High Hypoxic Risk |  | GSM437172 | -0.174427707 | Low Hypoxic Risk |
| GSM972327 | 0.045244981 | High Hypoxic Risk |  | TCGA-F5-6813-01 | -0.014680554 | High Hypoxic Risk |  | GSM437173 | -0.007374272 | High Hypoxic Risk |
| GSM972328 | -0.204888561 | Low Hypoxic Risk |  | TCGA-F5-6814-01 | -0.463805018 | Low Hypoxic Risk |  | GSM437174 | -0.02444621 | High Hypoxic Risk |
| GSM972329 | -0.279464241 | Low Hypoxic Risk |  | TCGA-F5-6861-01 | -0.151188085 | Low Hypoxic Risk |  | GSM437175 | -0.069799117 | High Hypoxic Risk |
| GSM972330 | -0.392139035 | Low Hypoxic Risk |  | TCGA-F5-6863-01 | 0.215508905 | High Hypoxic Risk |  | GSM437176 | -0.078425214 | High Hypoxic Risk |
| GSM972331 | -0.124658019 | Low Hypoxic Risk |  | TCGA-F5-6864-01 | -0.144340161 | Low Hypoxic Risk |  | GSM437177 | -0.141996142 | Low Hypoxic Risk |
| GSM972332 | -0.02080191 | High Hypoxic Risk |  | TCGA-G5-6233-01 | 0.02405389 | High Hypoxic Risk |  | GSM437178 | -0.240781901 | Low Hypoxic Risk |
| GSM972333 | -0.000447861 | High Hypoxic Risk |  | TCGA-G5-6235-01 | -0.077801514 | High Hypoxic Risk |  | GSM437179 | 0.011251136 | High Hypoxic Risk |
| GSM972334 | -0.286224178 | Low Hypoxic Risk |  | TCGA-G5-6572-01 | 0.04677851 | High Hypoxic Risk |  | GSM437180 | 0.031574197 | High Hypoxic Risk |
| GSM972335 | 0.017688033 | High Hypoxic Risk |  | TCGA-G5-6572-02 | 0.48124606 | High Hypoxic Risk |  | GSM437181 | 0.101930363 | High Hypoxic Risk |
| GSM972336 | 0.065832216 | High Hypoxic Risk |  | TCGA-G5-6641-01 | 0.030784388 | High Hypoxic Risk |  | GSM437182 | -0.092695863 | Low Hypoxic Risk |
| GSM972337 | -0.176232764 | Low Hypoxic Risk |  | TCGA-A6-2671-01 | 0.207086379 | High Hypoxic Risk |  | GSM437183 | -0.067216682 | High Hypoxic Risk |
| GSM972338 | 0.053180737 | High Hypoxic Risk |  | TCGA-A6-2672-01 | -0.268425388 | Low Hypoxic Risk |  | GSM437184 | -0.081659962 | High Hypoxic Risk |
| GSM972339 | 0.119905239 | High Hypoxic Risk |  | TCGA-A6-2674-01 | 0.293547635 | High Hypoxic Risk |  | GSM437185 | 0.274586968 | High Hypoxic Risk |
| GSM972340 | 0.018301346 | High Hypoxic Risk |  | TCGA-A6-2676-01 | -0.504772888 | Low Hypoxic Risk |  | GSM437186 | -0.273878975 | Low Hypoxic Risk |
| GSM972341 | -0.153476753 | Low Hypoxic Risk |  | TCGA-A6-2677-01 | -0.196903295 | Low Hypoxic Risk |  | GSM437187 | 0.179966816 | High Hypoxic Risk |
| GSM972342 | -0.078899605 | High Hypoxic Risk |  | TCGA-A6-2678-01 | -0.477872021 | Low Hypoxic Risk |  | GSM437188 | 0.267356422 | High Hypoxic Risk |
| GSM972343 | -0.315830448 | Low Hypoxic Risk |  | TCGA-A6-2679-01 | 1.432622579 | High Hypoxic Risk |  | GSM437189 | 0.153117275 | High Hypoxic Risk |
| GSM972344 | -0.081004621 | High Hypoxic Risk |  | TCGA-A6-2680-01 | 0.145624638 | High Hypoxic Risk |  | GSM437190 | 0.194514989 | High Hypoxic Risk |
| GSM972345 | -0.017968091 | High Hypoxic Risk |  | TCGA-A6-2681-01 | 0.223448959 | High Hypoxic Risk |  | GSM437191 | 0.161277914 | High Hypoxic Risk |
| GSM972346 | -0.076284465 | High Hypoxic Risk |  | TCGA-A6-2683-01 | 0.683162639 | High Hypoxic Risk |  | GSM437192 | -0.120223815 | Low Hypoxic Risk |
| GSM972347 | -0.183917099 | Low Hypoxic Risk |  | TCGA-A6-3807-01 | -0.101177324 | Low Hypoxic Risk |  | GSM437193 | -0.637964934 | Low Hypoxic Risk |
| GSM972348 | -0.397708867 | Low Hypoxic Risk |  | TCGA-A6-3808-01 | -0.045843704 | High Hypoxic Risk |  | GSM437194 | 0.182469112 | High Hypoxic Risk |
| GSM972349 | 0.326663355 | High Hypoxic Risk |  | TCGA-A6-3809-01 | -0.286699989 | Low Hypoxic Risk |  | GSM437195 | 0.176160015 | High Hypoxic Risk |
| GSM972350 | -0.445048093 | Low Hypoxic Risk |  | TCGA-A6-3810-01 | 0.077348485 | High Hypoxic Risk |  | GSM437196 | 0.151984488 | High Hypoxic Risk |
| GSM972351 | -0.14606105 | Low Hypoxic Risk |  | TCGA-A6-4107-01 | 0.246036092 | High Hypoxic Risk |  | GSM437197 | 0.255153524 | High Hypoxic Risk |
| GSM972352 | 0.232066121 | High Hypoxic Risk |  | TCGA-AA-3488-01 | 0.416745179 | High Hypoxic Risk |  | GSM437198 | -0.130833591 | Low Hypoxic Risk |
| GSM972353 | -0.438983124 | Low Hypoxic Risk |  | TCGA-AA-3494-01 | -0.093071865 | Low Hypoxic Risk |  | GSM437199 | 0.027136671 | High Hypoxic Risk |
| GSM972354 | -0.082672669 | High Hypoxic Risk |  | TCGA-AA-3510-01 | 0.100512624 | High Hypoxic Risk |  | GSM437200 | 0.083813367 | High Hypoxic Risk |
| GSM972355 | 0.011799438 | High Hypoxic Risk |  | TCGA-AA-3514-01 | 0.548927436 | High Hypoxic Risk |  | GSM437201 | 0.377217888 | High Hypoxic Risk |
| GSM972356 | -0.20724426 | Low Hypoxic Risk |  | TCGA-AA-3516-01 | 0.133057417 | High Hypoxic Risk |  | GSM437202 | -0.008659017 | High Hypoxic Risk |
| GSM972357 | -0.095154168 | Low Hypoxic Risk |  | TCGA-AA-3517-01 | -0.098271998 | Low Hypoxic Risk |  | GSM437203 | -0.218574269 | Low Hypoxic Risk |
| GSM972358 | -0.316443812 | Low Hypoxic Risk |  | TCGA-AA-3518-01 | -0.079730567 | High Hypoxic Risk |  | GSM437204 | 0.407713808 | High Hypoxic Risk |
| GSM972359 | 0.060037608 | High Hypoxic Risk |  | TCGA-AA-3519-01 | 0.110165779 | High Hypoxic Risk |  | GSM437205 | 0.225113021 | High Hypoxic Risk |
| GSM972360 | -0.060396282 | High Hypoxic Risk |  | TCGA-AA-3520-01 | -0.086827177 | Low Hypoxic Risk |  | GSM437206 | -0.097033166 | Low Hypoxic Risk |
| GSM972361 | 0.069029382 | High Hypoxic Risk |  | TCGA-AA-3521-01 | 0.118578947 | High Hypoxic Risk |  | GSM437207 | 0.148749059 | High Hypoxic Risk |
| GSM972362 | -0.185690118 | Low Hypoxic Risk |  | TCGA-AA-3522-01 | -0.13452136 | Low Hypoxic Risk |  | GSM437208 | 0.259197962 | High Hypoxic Risk |
| GSM972363 | -0.223479266 | Low Hypoxic Risk |  | TCGA-AA-3524-01 | -0.136557559 | Low Hypoxic Risk |  | GSM437209 | -0.000433711 | High Hypoxic Risk |
| GSM972364 | 0.246222478 | High Hypoxic Risk |  | TCGA-AA-3525-01 | 0.034766195 | High Hypoxic Risk |  | GSM437210 | -0.045533535 | High Hypoxic Risk |
| GSM972365 | 0.288641331 | High Hypoxic Risk |  | TCGA-AA-3527-01 | 0.585187235 | High Hypoxic Risk |  | GSM437211 | -0.213768545 | Low Hypoxic Risk |
| GSM972366 | 0.363654973 | High Hypoxic Risk |  | TCGA-AA-3529-01 | -0.038411504 | High Hypoxic Risk |  | GSM437212 | 0.065206906 | High Hypoxic Risk |
| GSM972367 | 0.600752579 | High Hypoxic Risk |  | TCGA-AA-3530-01 | -0.265654219 | Low Hypoxic Risk |  | GSM437213 | -0.043058493 | High Hypoxic Risk |
| GSM972368 | -0.403442428 | Low Hypoxic Risk |  | TCGA-AA-3531-01 | 0.055804779 | High Hypoxic Risk |  | GSM437214 | -0.004655859 | High Hypoxic Risk |
| GSM972369 | 0.408307358 | High Hypoxic Risk |  | TCGA-AA-3532-01 | -0.182492251 | Low Hypoxic Risk |  | GSM437215 | 0.28427284 | High Hypoxic Risk |
| GSM972370 | 0.177083836 | High Hypoxic Risk |  | TCGA-AA-3534-01 | -0.022602141 | High Hypoxic Risk |  | GSM437216 | -0.37838479 | Low Hypoxic Risk |
| GSM972371 | 0.227219606 | High Hypoxic Risk |  | TCGA-AA-3538-01 | -0.077499399 | High Hypoxic Risk |  | GSM437217 | -0.363370994 | Low Hypoxic Risk |
| GSM972372 | 0.330060594 | High Hypoxic Risk |  | TCGA-AA-3542-01 | 1.030391871 | High Hypoxic Risk |  | GSM437218 | 0.071057254 | High Hypoxic Risk |
| GSM972373 | 0.042161799 | High Hypoxic Risk |  | TCGA-AA-3543-01 | -0.055642388 | High Hypoxic Risk |  | GSM437219 | -0.081588351 | High Hypoxic Risk |
| GSM972374 | 0.005073071 | High Hypoxic Risk |  | TCGA-AA-3544-01 | 0.215122222 | High Hypoxic Risk |  | GSM437220 | 0.247345567 | High Hypoxic Risk |
| GSM972375 | 0.227277929 | High Hypoxic Risk |  | TCGA-AA-3548-01 | 0.551460192 | High Hypoxic Risk |  | GSM437221 | 0.025156895 | High Hypoxic Risk |
| GSM972376 | -0.04315987 | High Hypoxic Risk |  | TCGA-AA-3549-01 | 0.123115628 | High Hypoxic Risk |  | GSM437222 | -0.032946812 | High Hypoxic Risk |
| GSM972377 | 0.193557835 | High Hypoxic Risk |  | TCGA-AA-3552-01 | 0.01453939 | High Hypoxic Risk |  | GSM437223 | -0.013730645 | High Hypoxic Risk |
| GSM972378 | 0.479575859 | High Hypoxic Risk |  | TCGA-AA-3553-01 | -0.099861429 | Low Hypoxic Risk |  | GSM437224 | 0.037359706 | High Hypoxic Risk |
| GSM972379 | 0.228189927 | High Hypoxic Risk |  | TCGA-AA-3554-01 | -0.029566429 | High Hypoxic Risk |  | GSM437225 | -0.400436331 | Low Hypoxic Risk |
| GSM972380 | 0.138975612 | High Hypoxic Risk |  | TCGA-AA-3555-01 | -0.28859089 | Low Hypoxic Risk |  | GSM437226 | 0.131227343 | High Hypoxic Risk |
| GSM972381 | 0.599760147 | High Hypoxic Risk |  | TCGA-AA-3556-01 | -0.333011378 | Low Hypoxic Risk |  | GSM437227 | 0.119838421 | High Hypoxic Risk |
| GSM972382 | -0.059110978 | High Hypoxic Risk |  | TCGA-AA-3560-01 | 0.3067136 | High Hypoxic Risk |  | GSM437228 | 0.240068399 | High Hypoxic Risk |
| GSM972383 | -0.121871353 | Low Hypoxic Risk |  | TCGA-AA-3561-01 | 0.428366964 | High Hypoxic Risk |  | GSM437229 | -0.001724573 | High Hypoxic Risk |
| GSM972384 | -0.01551626 | High Hypoxic Risk |  | TCGA-AA-3562-01 | 0.132935748 | High Hypoxic Risk |  | GSM437230 | 0.021763516 | High Hypoxic Risk |
| GSM972385 | 0.264210689 | High Hypoxic Risk |  | TCGA-AA-3664-01 | -0.153318605 | Low Hypoxic Risk |  | GSM437231 | -0.099085695 | Low Hypoxic Risk |
| GSM972386 | 0.238162717 | High Hypoxic Risk |  | TCGA-AA-3666-01 | -0.060024681 | High Hypoxic Risk |  | GSM437232 | 0.285249849 | High Hypoxic Risk |
| GSM972387 | 0.046120486 | High Hypoxic Risk |  | TCGA-AA-3667-01 | 0.439904866 | High Hypoxic Risk |  | GSM437233 | -0.184510137 | Low Hypoxic Risk |
| GSM972388 | 0.002468795 | High Hypoxic Risk |  | TCGA-AA-3672-01 | -0.046259409 | High Hypoxic Risk |  | GSM437234 | 0.143140484 | High Hypoxic Risk |
| GSM972389 | -0.315260438 | Low Hypoxic Risk |  | TCGA-AA-3673-01 | -0.273914851 | Low Hypoxic Risk |  | GSM437235 | -0.089751867 | Low Hypoxic Risk |
| GSM972390 | 0.240852985 | High Hypoxic Risk |  | TCGA-AA-3678-01 | -0.288372867 | Low Hypoxic Risk |  | GSM437236 | 0.468110372 | High Hypoxic Risk |
| GSM972391 | 0.160528133 | High Hypoxic Risk |  | TCGA-AA-3679-01 | 0.478486824 | High Hypoxic Risk |  | GSM437237 | -0.064276727 | High Hypoxic Risk |
| GSM972392 | 0.307266302 | High Hypoxic Risk |  | TCGA-AA-3680-01 | -0.14772814 | Low Hypoxic Risk |  | GSM437238 | -0.04338789 | High Hypoxic Risk |
| GSM972393 | 0.183378978 | High Hypoxic Risk |  | TCGA-AA-3681-01 | -0.199551 | Low Hypoxic Risk |  | GSM437239 | 0.135948446 | High Hypoxic Risk |
| GSM972394 | 0.154576643 | High Hypoxic Risk |  | TCGA-AA-3684-01 | 0.555331181 | High Hypoxic Risk |  | GSM437240 | -0.05574007 | High Hypoxic Risk |
| GSM972395 | -0.032690475 | High Hypoxic Risk |  | TCGA-AA-3688-01 | -0.006210084 | High Hypoxic Risk |  | GSM437241 | 0.146113734 | High Hypoxic Risk |
| GSM972396 | -0.308336999 | Low Hypoxic Risk |  | TCGA-AA-3692-01 | 0.015595138 | High Hypoxic Risk |  | GSM437242 | -0.042880308 | High Hypoxic Risk |
| GSM972397 | 0.173106367 | High Hypoxic Risk |  | TCGA-AA-3693-01 | -0.004904914 | High Hypoxic Risk |  | GSM437243 | 0.031853613 | High Hypoxic Risk |
| GSM972398 | -0.273472752 | Low Hypoxic Risk |  | TCGA-AA-3696-01 | -0.002052288 | High Hypoxic Risk |  | GSM437244 | 0.056534181 | High Hypoxic Risk |
| GSM972399 | -0.042631918 | High Hypoxic Risk |  | TCGA-AA-3710-01 | -0.10491217 | Low Hypoxic Risk |  | GSM437245 | 0.074673839 | High Hypoxic Risk |
| GSM972400 | 0.056978298 | High Hypoxic Risk |  | TCGA-AA-3715-01 | 0.261710651 | High Hypoxic Risk |  | GSM437246 | -0.115116267 | Low Hypoxic Risk |
| GSM972401 | 0.161265378 | High Hypoxic Risk |  | TCGA-AA-3811-01 | 0.134313345 | High Hypoxic Risk |  | GSM437247 | -0.078289123 | High Hypoxic Risk |
| GSM972402 | 0.041284605 | High Hypoxic Risk |  | TCGA-AA-3812-01 | 0.671596623 | High Hypoxic Risk |  | GSM437248 | -0.020273904 | High Hypoxic Risk |
| GSM972403 | -0.142430385 | Low Hypoxic Risk |  | TCGA-AA-3814-01 | 0.189251364 | High Hypoxic Risk |  | GSM437249 | -0.296575405 | Low Hypoxic Risk |
| GSM972404 | -0.137049902 | Low Hypoxic Risk |  | TCGA-AA-3815-01 | -0.439317482 | Low Hypoxic Risk |  | GSM437250 | -0.015617915 | High Hypoxic Risk |
| GSM972405 | 0.196187905 | High Hypoxic Risk |  | TCGA-AA-3818-01 | 0.083411645 | High Hypoxic Risk |  | GSM437251 | -0.051821723 | High Hypoxic Risk |
| GSM972406 | -0.297130685 | Low Hypoxic Risk |  | TCGA-AA-3819-01 | -0.004328397 | High Hypoxic Risk |  | GSM437252 | 0.016035068 | High Hypoxic Risk |
| GSM972407 | -0.055387364 | High Hypoxic Risk |  | TCGA-AA-3821-01 | -0.183766455 | Low Hypoxic Risk |  | GSM437253 | 0.253096675 | High Hypoxic Risk |
| GSM972408 | -0.003268497 | High Hypoxic Risk |  | TCGA-AA-3831-01 | -0.017756615 | High Hypoxic Risk |  | GSM437254 | 0.02550024 | High Hypoxic Risk |
| GSM972409 | 0.000726222 | High Hypoxic Risk |  | TCGA-AA-3833-01 | 0.046985046 | High Hypoxic Risk |  | GSM437255 | 0.049470065 | High Hypoxic Risk |
| GSM972410 | 0.553250157 | High Hypoxic Risk |  | TCGA-AA-3837-01 | 0.019960208 | High Hypoxic Risk |  | GSM437256 | 0.244359011 | High Hypoxic Risk |
| GSM972411 | -0.097009345 | Low Hypoxic Risk |  | TCGA-AA-3841-01 | 0.451932597 | High Hypoxic Risk |  | GSM437257 | -0.441076268 | Low Hypoxic Risk |
| GSM972412 | 0.155496955 | High Hypoxic Risk |  | TCGA-AA-3842-01 | -0.191571342 | Low Hypoxic Risk |  | GSM437258 | -0.232975202 | Low Hypoxic Risk |
| GSM972413 | 0.268215548 | High Hypoxic Risk |  | TCGA-AA-3844-01 | -0.007769303 | High Hypoxic Risk |  | GSM437259 | 0.151329756 | High Hypoxic Risk |
| GSM972414 | -0.134010084 | Low Hypoxic Risk |  | TCGA-AA-3845-01 | -0.187645721 | Low Hypoxic Risk |  | GSM437260 | 0.074342652 | High Hypoxic Risk |
| GSM972415 | -0.187661533 | Low Hypoxic Risk |  | TCGA-AA-3846-01 | -0.167161673 | Low Hypoxic Risk |  | GSM437261 | -0.052645561 | High Hypoxic Risk |
| GSM972416 | 0.175604149 | High Hypoxic Risk |  | TCGA-AA-3848-01 | 0.314600858 | High Hypoxic Risk |  | GSM437262 | 0.232362605 | High Hypoxic Risk |
| GSM972417 | 0.058138671 | High Hypoxic Risk |  | TCGA-AA-3850-01 | 0.084989415 | High Hypoxic Risk |  | GSM437263 | 0.057860464 | High Hypoxic Risk |
| GSM972418 | -0.264305018 | Low Hypoxic Risk |  | TCGA-AA-3851-01 | 0.148747737 | High Hypoxic Risk |  | GSM437264 | 0.199851884 | High Hypoxic Risk |
| GSM972419 | 0.499260366 | High Hypoxic Risk |  | TCGA-AA-3852-01 | 0.075201102 | High Hypoxic Risk |  | GSM437265 | 0.147861083 | High Hypoxic Risk |
| GSM972420 | 0.351684629 | High Hypoxic Risk |  | TCGA-AA-3854-01 | -0.049721927 | High Hypoxic Risk |  | GSM437266 | -0.030320564 | High Hypoxic Risk |
| GSM972421 | 0.229090784 | High Hypoxic Risk |  | TCGA-AA-3855-01 | 0.134525091 | High Hypoxic Risk |  | GSM437267 | -0.16289669 | Low Hypoxic Risk |
| GSM972422 | 0.606228719 | High Hypoxic Risk |  | TCGA-AA-3856-01 | -0.024140497 | High Hypoxic Risk |  | GSM437268 | 9.07E-05 | High Hypoxic Risk |
| GSM972423 | -0.044774488 | High Hypoxic Risk |  | TCGA-AA-3858-01 | 0.104133268 | High Hypoxic Risk |  | GSM437269 | -0.328800602 | Low Hypoxic Risk |
| GSM972424 | 0.355364007 | High Hypoxic Risk |  | TCGA-AA-3860-01 | 0.267600265 | High Hypoxic Risk |  | GSM820048 | -0.229420028 | Low Hypoxic Risk |
| GSM972425 | 0.148128499 | High Hypoxic Risk |  | TCGA-AA-3861-01 | 0.009486715 | High Hypoxic Risk |  | GSM820049 | 0.134828543 | High Hypoxic Risk |
| GSM972426 | 0.556214438 | High Hypoxic Risk |  | TCGA-AA-3862-01 | -0.158780536 | Low Hypoxic Risk |  | GSM820050 | -0.542643178 | Low Hypoxic Risk |
| GSM972427 | 0.072045416 | High Hypoxic Risk |  | TCGA-AA-3864-01 | -0.278397018 | Low Hypoxic Risk |  | GSM820051 | -0.306414095 | Low Hypoxic Risk |
| GSM972428 | 0.508814294 | High Hypoxic Risk |  | TCGA-AA-3866-01 | -0.33871893 | Low Hypoxic Risk |  | GSM820052 | -0.092755123 | Low Hypoxic Risk |
| GSM972429 | 0.138973342 | High Hypoxic Risk |  | TCGA-AA-3867-01 | 0.020539805 | High Hypoxic Risk |  | GSM820053 | -0.341405084 | Low Hypoxic Risk |
| GSM972430 | -0.062798708 | High Hypoxic Risk |  | TCGA-AA-3869-01 | 0.039119455 | High Hypoxic Risk |  | GSM820054 | -0.295755387 | Low Hypoxic Risk |
| GSM972431 | -0.136938281 | Low Hypoxic Risk |  | TCGA-AA-3870-01 | 0.390192025 | High Hypoxic Risk |  | GSM820055 | -0.101910116 | Low Hypoxic Risk |
| GSM972432 | 0.2253981 | High Hypoxic Risk |  | TCGA-AA-3872-01 | 0.256110163 | High Hypoxic Risk |  | GSM820056 | -0.32901319 | Low Hypoxic Risk |
| GSM972433 | 0.165052723 | High Hypoxic Risk |  | TCGA-AA-3875-01 | -0.171836723 | Low Hypoxic Risk |  | GSM820057 | -0.233461063 | Low Hypoxic Risk |
| GSM972434 | -0.17861545 | Low Hypoxic Risk |  | TCGA-AA-3877-01 | -0.09869166 | Low Hypoxic Risk |  | GSM820058 | -0.281277196 | Low Hypoxic Risk |
| GSM972435 | -0.070554845 | High Hypoxic Risk |  | TCGA-AA-3930-01 | -0.107622692 | Low Hypoxic Risk |  | GSM820059 | -0.151074216 | Low Hypoxic Risk |
| GSM972436 | 0.196892505 | High Hypoxic Risk |  | TCGA-AA-3939-01 | 0.291206926 | High Hypoxic Risk |  | GSM820060 | 0.175519617 | High Hypoxic Risk |
| GSM972437 | 0.102985415 | High Hypoxic Risk |  | TCGA-AA-3941-01 | 0.248592734 | High Hypoxic Risk |  | GSM820061 | 0.243436788 | High Hypoxic Risk |
| GSM972438 | 0.019083197 | High Hypoxic Risk |  | TCGA-AA-3947-01 | -0.278160673 | Low Hypoxic Risk |  | GSM820062 | -0.224073632 | Low Hypoxic Risk |
| GSM972439 | -0.060803238 | High Hypoxic Risk |  | TCGA-AA-3949-01 | -0.144230279 | Low Hypoxic Risk |  | GSM820063 | -0.390949885 | Low Hypoxic Risk |
| GSM972440 | -0.261257721 | Low Hypoxic Risk |  | TCGA-AA-3950-01 | -0.348390183 | Low Hypoxic Risk |  | GSM820064 | -0.344978944 | Low Hypoxic Risk |
| GSM972441 | 0.223841003 | High Hypoxic Risk |  | TCGA-AA-3952-01 | 0.854593288 | High Hypoxic Risk |  | GSM820065 | -0.186774819 | Low Hypoxic Risk |
| GSM972442 | -0.142122761 | Low Hypoxic Risk |  | TCGA-AA-3955-01 | -0.118254034 | Low Hypoxic Risk |  | GSM820066 | -0.248719768 | Low Hypoxic Risk |
| GSM972443 | -0.353656416 | Low Hypoxic Risk |  | TCGA-AA-3956-01 | -0.132705973 | Low Hypoxic Risk |  | GSM820067 | -0.168814798 | Low Hypoxic Risk |
| GSM972444 | -0.268540143 | Low Hypoxic Risk |  | TCGA-AA-3966-01 | -0.110293336 | Low Hypoxic Risk |  | GSM820068 | 0.031389697 | High Hypoxic Risk |
| GSM972445 | -0.713259692 | Low Hypoxic Risk |  | TCGA-AA-3968-01 | -0.237281321 | Low Hypoxic Risk |  | GSM820069 | -0.098423254 | Low Hypoxic Risk |
| GSM972446 | 0.186243946 | High Hypoxic Risk |  | TCGA-AA-3970-01 | -0.210603656 | Low Hypoxic Risk |  | GSM820070 | 0.108584712 | High Hypoxic Risk |
| GSM972447 | -0.300150234 | Low Hypoxic Risk |  | TCGA-AA-3971-01 | -0.06019 | High Hypoxic Risk |  | GSM820071 | -0.225006827 | Low Hypoxic Risk |
| GSM972448 | 0.301525207 | High Hypoxic Risk |  | TCGA-AA-3972-01 | 0.156519898 | High Hypoxic Risk |  | GSM820072 | 0.12305676 | High Hypoxic Risk |
| GSM972449 | 0.466041484 | High Hypoxic Risk |  | TCGA-AA-3973-01 | 0.029068702 | High Hypoxic Risk |  | GSM820073 | -0.314981444 | Low Hypoxic Risk |
| GSM972450 | -0.081974299 | High Hypoxic Risk |  | TCGA-AA-3975-01 | 0.215617526 | High Hypoxic Risk |  | GSM820074 | -0.1831099 | Low Hypoxic Risk |
| GSM972451 | 0.020962738 | High Hypoxic Risk |  | TCGA-AA-3976-01 | 0.074885683 | High Hypoxic Risk |  | GSM820075 | 0.161854831 | High Hypoxic Risk |
| GSM972452 | 0.043544871 | High Hypoxic Risk |  | TCGA-AA-3977-01 | -0.416229075 | Low Hypoxic Risk |  | GSM820076 | -0.014979925 | High Hypoxic Risk |
| GSM972453 | -0.105408262 | Low Hypoxic Risk |  | TCGA-AA-3979-01 | -0.084149994 | Low Hypoxic Risk |  | GSM820077 | 0.262102825 | High Hypoxic Risk |
| GSM972454 | -0.200943645 | Low Hypoxic Risk |  | TCGA-AA-3980-01 | -0.12861706 | Low Hypoxic Risk |  | GSM820078 | 0.274922919 | High Hypoxic Risk |
| GSM972455 | -0.140094724 | Low Hypoxic Risk |  | TCGA-AA-3982-01 | -0.08825622 | Low Hypoxic Risk |  | GSM820079 | -0.167040492 | Low Hypoxic Risk |
| GSM972456 | -0.175506154 | Low Hypoxic Risk |  | TCGA-AA-3984-01 | -0.360298916 | Low Hypoxic Risk |  | GSM820080 | -0.232465939 | Low Hypoxic Risk |
| GSM972457 | -0.154168439 | Low Hypoxic Risk |  | TCGA-AA-3986-01 | -0.145169096 | Low Hypoxic Risk |  | GSM820081 | 0.254092316 | High Hypoxic Risk |
| GSM972458 | -0.136331743 | Low Hypoxic Risk |  | TCGA-AA-3989-01 | 0.183357588 | High Hypoxic Risk |  | GSM820082 | 0.088801806 | High Hypoxic Risk |
| GSM972459 | -0.147103128 | Low Hypoxic Risk |  | TCGA-AA-3994-01 | 0.359146975 | High Hypoxic Risk |  | GSM820083 | 0.292943295 | High Hypoxic Risk |
| GSM972460 | -0.143528928 | Low Hypoxic Risk |  | TCGA-AA-A004-01 | 0.755746671 | High Hypoxic Risk |  | GSM820084 | -0.214655857 | Low Hypoxic Risk |
| GSM972461 | -0.197434134 | Low Hypoxic Risk |  | TCGA-AA-A00A-01 | -0.230136079 | Low Hypoxic Risk |  | GSM820085 | -0.021353867 | High Hypoxic Risk |
| GSM972462 | 0.026125227 | High Hypoxic Risk |  | TCGA-AA-A00D-01 | -0.253568896 | Low Hypoxic Risk |  | GSM820086 | 0.238382127 | High Hypoxic Risk |
| GSM972463 | -0.039756683 | High Hypoxic Risk |  | TCGA-AA-A00E-01 | -0.211179859 | Low Hypoxic Risk |  | GSM820087 | -0.066985216 | High Hypoxic Risk |
| GSM972464 | 0.038162593 | High Hypoxic Risk |  | TCGA-AA-A00F-01 | 0.247998482 | High Hypoxic Risk |  | GSM820088 | 0.036689507 | High Hypoxic Risk |
| GSM972465 | -0.042322248 | High Hypoxic Risk |  | TCGA-AA-A00J-01 | -0.085164925 | Low Hypoxic Risk |  | GSM820089 | 0.156001767 | High Hypoxic Risk |
| GSM972466 | -0.251381279 | Low Hypoxic Risk |  | TCGA-AA-A00K-01 | 0.035275716 | High Hypoxic Risk |  | GSM820090 | 0.187454396 | High Hypoxic Risk |
| GSM972467 | 0.27164274 | High Hypoxic Risk |  | TCGA-AA-A00L-01 | 0.102798544 | High Hypoxic Risk |  | GSM820091 | 0.202178791 | High Hypoxic Risk |
| GSM972468 | -0.309595965 | Low Hypoxic Risk |  | TCGA-AA-A00N-01 | -0.02717095 | High Hypoxic Risk |  | GSM820092 | 0.449414476 | High Hypoxic Risk |
| GSM972469 | -0.017234447 | High Hypoxic Risk |  | TCGA-AA-A00O-01 | 0.042663251 | High Hypoxic Risk |  | GSM820093 | 0.429352022 | High Hypoxic Risk |
| GSM972470 | 0.062597364 | High Hypoxic Risk |  | TCGA-AA-A00Q-01 | 0.109789136 | High Hypoxic Risk |  | GSM820094 | 0.257629288 | High Hypoxic Risk |
| GSM972471 | -0.322896675 | Low Hypoxic Risk |  | TCGA-AA-A00R-01 | -0.432702871 | Low Hypoxic Risk |  | GSM820095 | -0.079969607 | High Hypoxic Risk |
| GSM972472 | 0.158987115 | High Hypoxic Risk |  | TCGA-AA-A00U-01 | -0.202259108 | Low Hypoxic Risk |  | GSM820096 | 0.007061768 | High Hypoxic Risk |
| GSM972473 | 0.074550576 | High Hypoxic Risk |  | TCGA-AA-A00W-01 | -0.083353768 | Low Hypoxic Risk |  | GSM820097 | 0.290190155 | High Hypoxic Risk |
| GSM972474 | -0.03808715 | High Hypoxic Risk |  | TCGA-AA-A00Z-01 | -0.239429842 | Low Hypoxic Risk |  | GSM820098 | -0.264192689 | Low Hypoxic Risk |
| GSM972475 | -0.063321824 | High Hypoxic Risk |  | TCGA-AA-A010-01 | -0.050749091 | High Hypoxic Risk |  | GSM820099 | 0.193512248 | High Hypoxic Risk |
| GSM972476 | 0.347945843 | High Hypoxic Risk |  | TCGA-AA-A017-01 | 0.085647386 | High Hypoxic Risk |  | GSM820100 | 0.352261314 | High Hypoxic Risk |
| GSM972477 | 0.273144046 | High Hypoxic Risk |  | TCGA-AA-A01C-01 | 0.29743928 | High Hypoxic Risk |  | GSM820101 | -0.108526517 | Low Hypoxic Risk |
| GSM972478 | -0.110815754 | Low Hypoxic Risk |  | TCGA-AA-A01D-01 | 0.025427863 | High Hypoxic Risk |  | GSM820102 | 0.463691798 | High Hypoxic Risk |
| GSM972479 | 0.514220486 | High Hypoxic Risk |  | TCGA-AA-A01F-01 | 0.445844321 | High Hypoxic Risk |  | GSM820103 | 0.127720947 | High Hypoxic Risk |
| GSM972480 | -0.202898619 | Low Hypoxic Risk |  | TCGA-AA-A01G-01 | -0.178102168 | Low Hypoxic Risk |  | GSM820104 | 0.213221761 | High Hypoxic Risk |
| GSM972481 | 0.285535531 | High Hypoxic Risk |  | TCGA-AA-A01I-01 | -0.212840971 | Low Hypoxic Risk |  | GSM820105 | 0.018518512 | High Hypoxic Risk |
| GSM972482 | 0.16828039 | High Hypoxic Risk |  | TCGA-AA-A01K-01 | 0.005859347 | High Hypoxic Risk |  | GSM820106 | 0.088549516 | High Hypoxic Risk |
| GSM972483 | 0.291353959 | High Hypoxic Risk |  | TCGA-AA-A01Q-01 | 0.072078673 | High Hypoxic Risk |  | GSM820107 | 0.166533075 | High Hypoxic Risk |
| GSM972484 | 0.083680701 | High Hypoxic Risk |  | TCGA-AA-A01R-01 | 0.02466842 | High Hypoxic Risk |  | GSM820108 | 0.207341658 | High Hypoxic Risk |
| GSM972485 | -0.164118875 | Low Hypoxic Risk |  | TCGA-AA-A01S-01 | 0.183887757 | High Hypoxic Risk |  | GSM820109 | 0.273352582 | High Hypoxic Risk |
| GSM972486 | 0.008167349 | High Hypoxic Risk |  | TCGA-AA-A01T-01 | 0.932458754 | High Hypoxic Risk |  | GSM820110 | 0.446309219 | High Hypoxic Risk |
| GSM972487 | -0.06562101 | High Hypoxic Risk |  | TCGA-AA-A01V-01 | 0.052047498 | High Hypoxic Risk |  | GSM820111 | 0.162156945 | High Hypoxic Risk |
| GSM972488 | -0.186626532 | Low Hypoxic Risk |  | TCGA-AA-A022-01 | 0.182066079 | High Hypoxic Risk |  | GSM820112 | 0.206633875 | High Hypoxic Risk |
| GSM972489 | 0.126870653 | High Hypoxic Risk |  | TCGA-AA-A024-01 | 0.25960545 | High Hypoxic Risk |  | GSM820113 | 0.014163409 | High Hypoxic Risk |
| GSM972490 | -0.175329365 | Low Hypoxic Risk |  | TCGA-AA-A029-01 | 0.200935055 | High Hypoxic Risk |  | GSM820114 | -0.056627631 | High Hypoxic Risk |
| GSM972491 | 0.007615483 | High Hypoxic Risk |  | TCGA-AA-A02E-01 | 0.303252717 | High Hypoxic Risk |  | GSM820115 | -0.095678818 | Low Hypoxic Risk |
| GSM972492 | -0.210444923 | Low Hypoxic Risk |  | TCGA-AA-A02F-01 | 0.301750196 | High Hypoxic Risk |  | GSM820116 | 0.112127591 | High Hypoxic Risk |
| GSM972493 | -0.040302395 | High Hypoxic Risk |  | TCGA-AA-A02H-01 | 0.122882835 | High Hypoxic Risk |  | GSM820117 | -0.098138333 | Low Hypoxic Risk |
| GSM972494 | -0.027532696 | High Hypoxic Risk |  | TCGA-AA-A02J-01 | -0.054216495 | High Hypoxic Risk |  | GSM820118 | -0.277233665 | Low Hypoxic Risk |
| GSM972495 | -0.017372205 | High Hypoxic Risk |  | TCGA-AA-A02O-01 | 0.522183645 | High Hypoxic Risk |  | GSM820119 | 0.283165978 | High Hypoxic Risk |
| GSM972496 | -0.473246594 | Low Hypoxic Risk |  | TCGA-AA-A02R-01 | -0.241558951 | Low Hypoxic Risk |  | GSM820120 | -0.067005266 | High Hypoxic Risk |
| GSM972497 | -0.376647806 | Low Hypoxic Risk |  | TCGA-AA-A02W-01 | -0.192528574 | Low Hypoxic Risk |  | GSM820121 | -0.138252445 | Low Hypoxic Risk |
| GSM972498 | -0.165027505 | Low Hypoxic Risk |  | TCGA-AA-A03F-01 | 0.375419506 | High Hypoxic Risk |  | GSM820122 | 0.064992281 | High Hypoxic Risk |
| GSM972499 | -0.560182804 | Low Hypoxic Risk |  | TCGA-AA-A03J-01 | 0.457071688 | High Hypoxic Risk |  | GSM820123 | -0.108842515 | Low Hypoxic Risk |
| GSM972500 | 0.841790707 | High Hypoxic Risk |  | TCGA-AY-4070-01 | 0.075503119 | High Hypoxic Risk |  | GSM820124 | -0.331382257 | Low Hypoxic Risk |
| GSM972501 | 0.363951729 | High Hypoxic Risk |  | TCGA-AY-4071-01 | 0.186487936 | High Hypoxic Risk |  | GSM820125 | 0.116249797 | High Hypoxic Risk |
| GSM972502 | 0.304634769 | High Hypoxic Risk |  | TCGA-AZ-4308-01 | 0.966104644 | High Hypoxic Risk |  | GSM820126 | -0.146215298 | Low Hypoxic Risk |
| GSM972503 | -0.057438036 | High Hypoxic Risk |  | TCGA-AZ-4681-01 | 0.485744393 | High Hypoxic Risk |  | GSM820127 | 0.060938787 | High Hypoxic Risk |
| GSM972504 | 0.194893533 | High Hypoxic Risk |  | TCGA-CM-4746-01 | 0.352168781 | High Hypoxic Risk |  | GSM820128 | -0.112560955 | Low Hypoxic Risk |
| GSM972505 | 0.301370177 | High Hypoxic Risk |  | TCGA-CM-4748-01 | -0.081140154 | High Hypoxic Risk |  | GSM820129 | 0.028931353 | High Hypoxic Risk |
| GSM972506 | 0.295021851 | High Hypoxic Risk |  | TCGA-CM-4750-01 | 0.315050868 | High Hypoxic Risk |  | GSM820130 | -0.018069368 | High Hypoxic Risk |
| GSM972507 | -0.202512038 | Low Hypoxic Risk |  | TCGA-CM-4752-01 | -0.030517195 | High Hypoxic Risk |  | GSM820131 | -0.245661241 | Low Hypoxic Risk |
| GSM972508 | -0.222942909 | Low Hypoxic Risk |  | TCGA-CM-5341-01 | 0.405415289 | High Hypoxic Risk |  | GSM820132 | 0.010431757 | High Hypoxic Risk |
| GSM972509 | -0.246532186 | Low Hypoxic Risk |  | TCGA-AF-2691-01 | 0.061009301 | High Hypoxic Risk |  | GSM820133 | 0.147660534 | High Hypoxic Risk |
| GSM972510 | -0.132846409 | Low Hypoxic Risk |  | TCGA-AF-2692-01 | 0.179415235 | High Hypoxic Risk |  | GSM820134 | 0.020414258 | High Hypoxic Risk |
| GSM972511 | -0.095146169 | Low Hypoxic Risk |  | TCGA-AF-3400-01 | 0.231111828 | High Hypoxic Risk |  | GSM820135 | -0.012515395 | High Hypoxic Risk |
| GSM972512 | -0.201964936 | Low Hypoxic Risk |  | TCGA-AF-3913-01 | 0.06281903 | High Hypoxic Risk |  | GSM820136 | 0.083652841 | High Hypoxic Risk |
| GSM972513 | 0.045507684 | High Hypoxic Risk |  | TCGA-AG-3574-01 | 0.356358909 | High Hypoxic Risk |  | GSM820137 | -0.0991052 | Low Hypoxic Risk |
| GSM972514 | -0.08587315 | Low Hypoxic Risk |  | TCGA-AG-3575-01 | 0.246518767 | High Hypoxic Risk |  | GSM929494 | -0.143147361 | Low Hypoxic Risk |
| GSM972515 | -0.170513297 | Low Hypoxic Risk |  | TCGA-AG-3578-01 | -0.137676794 | Low Hypoxic Risk |  | GSM929495 | -0.411966852 | Low Hypoxic Risk |
| GSM972516 | -0.181474418 | Low Hypoxic Risk |  | TCGA-AG-3580-01 | -0.257045325 | Low Hypoxic Risk |  | GSM929496 | 0.284472213 | High Hypoxic Risk |
| GSM972517 | -0.173801838 | Low Hypoxic Risk |  | TCGA-AG-3581-01 | 0.231692034 | High Hypoxic Risk |  | GSM929497 | -0.276515357 | Low Hypoxic Risk |
| GSM972518 | 0.245647966 | High Hypoxic Risk |  | TCGA-AG-3582-01 | -0.027403553 | High Hypoxic Risk |  | GSM929498 | -0.081907847 | High Hypoxic Risk |
| GSM972519 | 0.570018144 | High Hypoxic Risk |  | TCGA-AG-3583-01 | 0.106145717 | High Hypoxic Risk |  | GSM929499 | -0.090408734 | Low Hypoxic Risk |
| GSM972520 | -0.254916919 | Low Hypoxic Risk |  | TCGA-AG-3584-01 | -0.019045035 | High Hypoxic Risk |  | GSM929500 | 0.143753339 | High Hypoxic Risk |
| GSM972521 | -0.315674386 | Low Hypoxic Risk |  | TCGA-AG-3586-01 | -0.020927635 | High Hypoxic Risk |  | GSM929501 | 0.078829473 | High Hypoxic Risk |
| GSM972522 | -0.005850075 | High Hypoxic Risk |  | TCGA-AG-3587-01 | 0.065695371 | High Hypoxic Risk |  | GSM929502 | -0.138822495 | Low Hypoxic Risk |
|  |  |  |  | TCGA-AG-3593-01 | -0.054295133 | High Hypoxic Risk |  | GSM929503 | 0.247129164 | High Hypoxic Risk |
|  |  |  |  | TCGA-AG-3594-01 | -0.119037036 | Low Hypoxic Risk |  | GSM929504 | 0.177519086 | High Hypoxic Risk |
|  |  |  |  | TCGA-AG-3598-01 | 0.139388092 | High Hypoxic Risk |  | GSM929505 | -0.166260725 | Low Hypoxic Risk |
|  |  |  |  | TCGA-AG-3599-01 | -0.132458192 | Low Hypoxic Risk |  | GSM929506 | -0.279954792 | Low Hypoxic Risk |
|  |  |  |  | TCGA-AG-3600-01 | 0.180089796 | High Hypoxic Risk |  | GSM929507 | 0.056998221 | High Hypoxic Risk |
|  |  |  |  | TCGA-AG-3601-01 | 0.496832757 | High Hypoxic Risk |  | GSM929508 | 0.19304065 | High Hypoxic Risk |
|  |  |  |  | TCGA-AG-3602-01 | 0.646974776 | High Hypoxic Risk |  | GSM929509 | 0.013400751 | High Hypoxic Risk |
|  |  |  |  | TCGA-AG-3605-01 | 0.373134964 | High Hypoxic Risk |  | GSM929510 | -0.129090809 | Low Hypoxic Risk |
|  |  |  |  | TCGA-AG-3608-01 | 0.067298655 | High Hypoxic Risk |  | GSM929511 | -0.181300215 | Low Hypoxic Risk |
|  |  |  |  | TCGA-AG-3609-01 | 0.322960608 | High Hypoxic Risk |  | GSM929512 | 0.215322214 | High Hypoxic Risk |
|  |  |  |  | TCGA-AG-3611-01 | 0.466574778 | High Hypoxic Risk |  | GSM929513 | 0.073530801 | High Hypoxic Risk |
|  |  |  |  | TCGA-AG-3612-01 | 0.198325817 | High Hypoxic Risk |  | GSM929514 | -0.052048713 | High Hypoxic Risk |
|  |  |  |  | TCGA-AG-3726-01 | 0.183332513 | High Hypoxic Risk |  | GSM929515 | -0.038776038 | High Hypoxic Risk |
|  |  |  |  | TCGA-AG-3727-01 | 0.351039211 | High Hypoxic Risk |  | GSM929516 | -0.254346656 | Low Hypoxic Risk |
|  |  |  |  | TCGA-AG-3728-01 | 0.324161203 | High Hypoxic Risk |  | GSM929517 | -0.1086119 | Low Hypoxic Risk |
|  |  |  |  | TCGA-AG-3878-01 | 0.359408597 | High Hypoxic Risk |  | GSM929518 | -0.00296768 | High Hypoxic Risk |
|  |  |  |  | TCGA-AG-3881-01 | 0.926130945 | High Hypoxic Risk |  | GSM929519 | 0.085340292 | High Hypoxic Risk |
|  |  |  |  | TCGA-AG-3882-01 | 0.440974475 | High Hypoxic Risk |  | GSM929520 | 0.098590493 | High Hypoxic Risk |
|  |  |  |  | TCGA-AG-3883-01 | 0.772064913 | High Hypoxic Risk |  | GSM929521 | -0.139366194 | Low Hypoxic Risk |
|  |  |  |  | TCGA-AG-3885-01 | 0.53028254 | High Hypoxic Risk |  | GSM929522 | 0.331093191 | High Hypoxic Risk |
|  |  |  |  | TCGA-AG-3887-01 | 0.179799256 | High Hypoxic Risk |  | GSM929523 | -0.025169915 | High Hypoxic Risk |
|  |  |  |  | TCGA-AG-3890-01 | 0.048740722 | High Hypoxic Risk |  | GSM929524 | 0.094869677 | High Hypoxic Risk |
|  |  |  |  | TCGA-AG-3892-01 | -0.015977558 | High Hypoxic Risk |  | GSM929525 | 0.131882038 | High Hypoxic Risk |
|  |  |  |  | TCGA-AG-3893-01 | 0.08183596 | High Hypoxic Risk |  | GSM929526 | 0.073139912 | High Hypoxic Risk |
|  |  |  |  | TCGA-AG-3894-01 | 0.406051874 | High Hypoxic Risk |  | GSM929527 | 0.202892945 | High Hypoxic Risk |
|  |  |  |  | TCGA-AG-3896-01 | 0.161154455 | High Hypoxic Risk |  | GSM929528 | -0.276966238 | Low Hypoxic Risk |
|  |  |  |  | TCGA-AG-3898-01 | 0.112067269 | High Hypoxic Risk |  | GSM929529 | 0.17470547 | High Hypoxic Risk |
|  |  |  |  | TCGA-AG-3901-01 | -0.074426833 | High Hypoxic Risk |  | GSM929530 | -0.202974068 | Low Hypoxic Risk |
|  |  |  |  | TCGA-AG-3909-01 | 0.072749629 | High Hypoxic Risk |  | GSM929531 | -0.194281877 | Low Hypoxic Risk |
|  |  |  |  | TCGA-AG-3999-01 | 0.338532144 | High Hypoxic Risk |  | GSM929532 | -0.474993304 | Low Hypoxic Risk |
|  |  |  |  | TCGA-AG-4001-01 | 0.120485831 | High Hypoxic Risk |  | GSM929533 | -0.243664382 | Low Hypoxic Risk |
|  |  |  |  | TCGA-AG-4005-01 | 0.16663346 | High Hypoxic Risk |  | GSM929534 | -0.32379942 | Low Hypoxic Risk |
|  |  |  |  | TCGA-AG-4007-01 | 0.301292289 | High Hypoxic Risk |  | GSM929535 | -0.186318062 | Low Hypoxic Risk |
|  |  |  |  | TCGA-AG-4008-01 | 0.115267603 | High Hypoxic Risk |  | GSM929536 | -0.253575546 | Low Hypoxic Risk |
|  |  |  |  | TCGA-AG-4015-01 | 0.133199323 | High Hypoxic Risk |  | GSM929537 | -0.213043792 | Low Hypoxic Risk |
|  |  |  |  | TCGA-AG-A002-01 | -0.052651025 | High Hypoxic Risk |  | GSM929538 | -0.475195087 | Low Hypoxic Risk |
|  |  |  |  | TCGA-AG-A008-01 | -0.213222969 | Low Hypoxic Risk |  | GSM929539 | 0.008819465 | High Hypoxic Risk |
|  |  |  |  | TCGA-AG-A00C-01 | 0.193216395 | High Hypoxic Risk |  | GSM929540 | 0.013889319 | High Hypoxic Risk |
|  |  |  |  | TCGA-AG-A00H-01 | 0.271014567 | High Hypoxic Risk |  | GSM929541 | 0.070536559 | High Hypoxic Risk |
|  |  |  |  | TCGA-AG-A00Y-01 | 0.069945637 | High Hypoxic Risk |  | GSM929542 | -0.30462001 | Low Hypoxic Risk |
|  |  |  |  | TCGA-AG-A011-01 | 0.081729747 | High Hypoxic Risk |  | GSM929543 | -0.046283695 | High Hypoxic Risk |
|  |  |  |  | TCGA-AG-A014-01 | 0.23980758 | High Hypoxic Risk |  | GSM929544 | -0.008467359 | High Hypoxic Risk |
|  |  |  |  | TCGA-AG-A015-01 | 0.193175644 | High Hypoxic Risk |  | GSM929545 | -0.397245199 | Low Hypoxic Risk |
|  |  |  |  | TCGA-AG-A016-01 | 0.151180784 | High Hypoxic Risk |  | GSM929546 | -0.040485305 | High Hypoxic Risk |
|  |  |  |  | TCGA-AG-A01J-01 | 0.114483648 | High Hypoxic Risk |  | GSM929547 | -0.131976591 | Low Hypoxic Risk |
|  |  |  |  | TCGA-AG-A01L-01 | 0.078565116 | High Hypoxic Risk |  | GSM929548 | -0.016804753 | High Hypoxic Risk |
|  |  |  |  | TCGA-AG-A01N-01 | 0.102347305 | High Hypoxic Risk |  | GSM929549 | 0.101928046 | High Hypoxic Risk |
|  |  |  |  | TCGA-AG-A01W-01 | -0.126129404 | Low Hypoxic Risk |  | GSM929550 | -0.079640479 | High Hypoxic Risk |
|  |  |  |  | TCGA-AG-A01Y-01 | -0.280149499 | Low Hypoxic Risk |  | GSM929551 | -0.044257737 | High Hypoxic Risk |
|  |  |  |  | TCGA-AG-A020-01 | -0.025430637 | High Hypoxic Risk |  | GSM929552 | -0.240731015 | Low Hypoxic Risk |
|  |  |  |  | TCGA-AG-A023-01 | 0.139731948 | High Hypoxic Risk |  | GSM929553 | -0.094922275 | Low Hypoxic Risk |
|  |  |  |  | TCGA-AG-A025-01 | 0.006066608 | High Hypoxic Risk |  | GSM929554 | -0.068344825 | High Hypoxic Risk |
|  |  |  |  | TCGA-AG-A026-01 | 0.276251577 | High Hypoxic Risk |  | GSM929555 | 0.108798086 | High Hypoxic Risk |
|  |  |  |  | TCGA-AG-A02G-01 | -0.170588977 | Low Hypoxic Risk |  | GSM929556 | -0.283840667 | Low Hypoxic Risk |
|  |  |  |  | TCGA-AG-A02N-01 | -0.288119022 | Low Hypoxic Risk |  | GSM929557 | -0.365279421 | Low Hypoxic Risk |
|  |  |  |  | TCGA-AG-A02X-01 | -0.220465894 | Low Hypoxic Risk |  | GSM929558 | -0.351576872 | Low Hypoxic Risk |
|  |  |  |  | TCGA-AG-A032-01 | 0.284435579 | High Hypoxic Risk |  | GSM929559 | 0.161048882 | High Hypoxic Risk |
|  |  |  |  | TCGA-AG-A036-01 | -0.217951482 | Low Hypoxic Risk |  | GSM929560 | 0.041628556 | High Hypoxic Risk |
|  |  |  |  |  |  |  |  | GSM929561 | -0.369737137 | Low Hypoxic Risk |
|  |  |  |  |  |  |  |  | GSM929562 | 0.03469422 | High Hypoxic Risk |
|  |  |  |  |  |  |  |  | GSM929563 | -0.048925205 | High Hypoxic Risk |
|  |  |  |  |  |  |  |  | GSM929564 | -0.207840767 | Low Hypoxic Risk |
|  |  |  |  |  |  |  |  | GSM929565 | 0.162252569 | High Hypoxic Risk |
|  |  |  |  |  |  |  |  | GSM929566 | -0.235139534 | Low Hypoxic Risk |
|  |  |  |  |  |  |  |  | GSM929567 | 0.102771465 | High Hypoxic Risk |
|  |  |  |  |  |  |  |  | GSM929568 | -0.08241719 | High Hypoxic Risk |
|  |  |  |  |  |  |  |  | GSM929569 | 0.564089013 | High Hypoxic Risk |
|  |  |  |  |  |  |  |  | GSM929570 | -0.414693556 | Low Hypoxic Risk |
|  |  |  |  |  |  |  |  | GSM929571 | 0.04980047 | High Hypoxic Risk |
|  |  |  |  |  |  |  |  | GSM929572 | 0.619092651 | High Hypoxic Risk |
|  |  |  |  |  |  |  |  | GSM929573 | 0.048174619 | High Hypoxic Risk |
|  |  |  |  |  |  |  |  | GSM929574 | 0.367403472 | High Hypoxic Risk |
|  |  |  |  |  |  |  |  | GSM929575 | 0.594089258 | High Hypoxic Risk |
|  |  |  |  |  |  |  |  | GSM929576 | -0.002031919 | High Hypoxic Risk |
|  |  |  |  |  |  |  |  | GSM929577 | 0.287802209 | High Hypoxic Risk |
|  |  |  |  |  |  |  |  | GSM929578 | 0.522020361 | High Hypoxic Risk |
|  |  |  |  |  |  |  |  | GSM929579 | 0.218801486 | High Hypoxic Risk |
|  |  |  |  |  |  |  |  | GSM929580 | -0.3275674 | Low Hypoxic Risk |
|  |  |  |  |  |  |  |  | GSM929581 | 0.004083412 | High Hypoxic Risk |
|  |  |  |  |  |  |  |  | GSM929582 | 0.250231136 | High Hypoxic Risk |
|  |  |  |  |  |  |  |  | GSM929583 | 0.155762811 | High Hypoxic Risk |
|  |  |  |  |  |  |  |  | GSM929584 | -0.081418206 | High Hypoxic Risk |
|  |  |  |  |  |  |  |  | GSM929585 | 0.479884034 | High Hypoxic Risk |
|  |  |  |  |  |  |  |  | GSM929586 | 0.02140866 | High Hypoxic Risk |
|  |  |  |  |  |  |  |  | GSM929587 | 0.001526383 | High Hypoxic Risk |
|  |  |  |  |  |  |  |  | GSM929588 | -0.060755754 | High Hypoxic Risk |
|  |  |  |  |  |  |  |  | GSM929589 | 0.225378766 | High Hypoxic Risk |
|  |  |  |  |  |  |  |  | GSM929590 | 0.217015366 | High Hypoxic Risk |
|  |  |  |  |  |  |  |  | GSM929591 | 0.009204984 | High Hypoxic Risk |
|  |  |  |  |  |  |  |  | GSM929592 | 0.174474053 | High Hypoxic Risk |
|  |  |  |  |  |  |  |  | GSM929593 | 0.202975077 | High Hypoxic Risk |
|  |  |  |  |  |  |  |  | GSM929594 | 0.318262472 | High Hypoxic Risk |
|  |  |  |  |  |  |  |  | GSM929595 | -0.213687146 | Low Hypoxic Risk |
|  |  |  |  |  |  |  |  | GSM929596 | -0.107364757 | Low Hypoxic Risk |
|  |  |  |  |  |  |  |  | GSM929597 | 0.134023987 | High Hypoxic Risk |
|  |  |  |  |  |  |  |  | GSM929598 | -0.097597076 | Low Hypoxic Risk |
|  |  |  |  |  |  |  |  | GSM929599 | 0.228572745 | High Hypoxic Risk |
|  |  |  |  |  |  |  |  | GSM929600 | 0.098390908 | High Hypoxic Risk |
|  |  |  |  |  |  |  |  | GSM929601 | 0.086459281 | High Hypoxic Risk |
|  |  |  |  |  |  |  |  | GSM929602 | -0.043078009 | High Hypoxic Risk |
|  |  |  |  |  |  |  |  | GSM929603 | 0.271987438 | High Hypoxic Risk |
|  |  |  |  |  |  |  |  | GSM929604 | -0.089330095 | Low Hypoxic Risk |
|  |  |  |  |  |  |  |  | GSM929605 | 0.2192788 | High Hypoxic Risk |
|  |  |  |  |  |  |  |  | GSM929606 | 0.187730055 | High Hypoxic Risk |
|  |  |  |  |  |  |  |  | GSM929607 | -0.278102701 | Low Hypoxic Risk |
|  |  |  |  |  |  |  |  | GSM929608 | 0.187803431 | High Hypoxic Risk |
|  |  |  |  |  |  |  |  | GSM929609 | 0.078787983 | High Hypoxic Risk |
|  |  |  |  |  |  |  |  | GSM929610 | -0.035647024 | High Hypoxic Risk |
|  |  |  |  |  |  |  |  | GSM929611 | -0.146063922 | Low Hypoxic Risk |
|  |  |  |  |  |  |  |  | GSM929612 | 0.059350413 | High Hypoxic Risk |
|  |  |  |  |  |  |  |  | GSM929613 | -0.024333307 | High Hypoxic Risk |
|  |  |  |  |  |  |  |  | GSM929614 | -0.017331556 | High Hypoxic Risk |
|  |  |  |  |  |  |  |  | GSM929615 | -0.438266917 | Low Hypoxic Risk |
|  |  |  |  |  |  |  |  | GSM929616 | 0.261024964 | High Hypoxic Risk |
|  |  |  |  |  |  |  |  | GSM929617 | -0.009908318 | High Hypoxic Risk |
|  |  |  |  |  |  |  |  | GSM929618 | 0.344599984 | High Hypoxic Risk |
|  |  |  |  |  |  |  |  | GSM929619 | 0.030048997 | High Hypoxic Risk |
|  |  |  |  |  |  |  |  | GSM929620 | 0.116764092 | High Hypoxic Risk |
|  |  |  |  |  |  |  |  | GSM929621 | 0.015062923 | High Hypoxic Risk |
|  |  |  |  |  |  |  |  | GSM929622 | -0.006441913 | High Hypoxic Risk |
|  |  |  |  |  |  |  |  | GSM929623 | 0.043385887 | High Hypoxic Risk |
